# Heparin-Binding Protein: A Novel Biomarker Linking Four Different Cardiovascular Diseases

**DOI:** 10.1155/2020/9575373

**Published:** 2020-06-15

**Authors:** Yufeng Cai, Xueyan Zhang, Jie Shen, Boyue Jiang, Dehua Hu, Mingyi Zhao

**Affiliations:** ^1^Department of Pediatrics, The Third Xiangya Hospital, Central South University, Hunan, Changsha, China; ^2^Xiangya School of Life Science, Central South University, Changsha, Hunan, China

## Abstract

Cardiovascular diseases are an important group of diseases that seriously affect quality of life. Thus, their treatment warrants further study. Heparin-binding protein (HBP) is a granulocyte protein derived from neutrophils. When an infection occurs, neutrophils release HBP, which can lead to elevated HBP levels in the blood. Therefore, HBP family members are said to be important indicators of infection. However, basic evidence is still lacking to confirm the possible relationship between HBP and cardiovascular diseases. Using bioinformatics methods, we investigated the role of the HBP network in normal hearts and hearts from patients with cardiovascular disease. First, we used the Open Targets database to obtain a list of HBP-encoding mRNAs related to atherosclerosis, myocarditis, myocardial infarction, and myocardial ischemia. Then, we constructed an HBP gene interaction network map using STRING. Clustering coefficients were calculated using Cytoscape, and MCODE was used for subnet analysis. Finally, the proposed interstitial network of HBPs was established and analyzed by Metascape enrichment analysis of the relevant signaling pathways. The aggregation coefficient of the HBP interaction network was higher among hearts with the four cardiovascular diseases, atherosclerosis (0.496), myocarditis (0.631), myocardial infarction (0.532), and myocardial ischemia (0.551), than in normal hearts. Metascape analysis showed that “NABA_MATRISOME_ASSOCIATED” was a typical pathway with the highest *p* value associated with epithelialization in all four diseases. Moreover, a large number of important HBPs were identified that may be significant for the treatment of these diseases. Therefore, HBPs do have a highly atopic connectivity network in cardiovascular diseases, and specific HBPs or signaling pathways may be used as targets for the development of new treatments for cardiovascular diseases.

## 1. Introduction

As one of the most common diseases, the incidence and mortality of cardiovascular disease in China are on the rise. Cardiovascular diseases are characterized by acute onset, critical condition, and rapid progress. Atherosclerosis, myocarditis, myocardial infarction, and myocardial ischemia are the most common diseases in clinical practice, and most patients die due to misdiagnosis and delayed treatment. Atherosclerosis mainly involves large and medium-sized arteries, with lipid deposition of the intima, focal fibrosis of the intima, and formation of atherosclerotic plaques as the basic lesions. Myocarditis is the limited or diffused inflammatory lesion of the myocardium, in which the myocardium is infiltrated by inflammatory cells, accompanied by denaturation and necrosis of adjacent cardiomyocytes. Myocardial infarction is a disease in which the blood supply to the coronary arteries is interrupted and ischemia in the blood supply leads to extensive myocardial necrosis. Myocardial ischemia is a pathological condition in which the blood perfusion of the heart decreases, leading to the decrease of oxygen supply to the heart and abnormal energy metabolism of the heart. Therefore, it is urgent to find a representative biomarker that can indicate the incidence of the above diseases through its plasma levels and changes in its protein expression levels to improve treatment or even enable prevention. This work is promising for the development of new therapies for clinical application.

HBP is a protein released by activated neutrophils when they adhere to the endothelium or when they are stimulated by circulating bacterial metabolites. HBP can promote the rearrangement of the endothelial cytoskeleton, leading to the destruction of the vascular endothelial barrier, the migration of white blood cells from capillaries to sites of infection, and an increase in vascular permeability [1]. Studies have shown that HBP also plays an important role in the regulation of the inflammatory response. By activating monocytes/macrophages, inflammatory mediators, such as tumor necrosis factor and interferon, are released to amplify the inflammatory response, which is closely related to the occurrence of hypotension and circulatory failure [2].

Abnormal HBP plasma levels have been previously found in patients with acute kidney injury [3], sepsis [4], pancreatic diseases [5], lung injuries [6], immune system diseases [7], spontaneous bacterial peritonitis [8], and other diseases. However, although these basic clinical experiments serve as the auxiliary proof of the role of the HBP, they cannot fully reveal the interaction networks and genetic mechanisms involved. In this study, we used bioinformatics tools, such as Cytoscape and Metascape, to analyze the network of interactions among members of the HBP family in cardiovascular diseases, such as atherosclerosis, myocarditis, myocardial infarction, and myocardial ischemia. The interaction between HBPs is discussed from the perspective of gene and protein networks identified through bioinformatics analyses to elucidate the value of HBPs as novel biomarkers of the severity and prognosis of typical cardiovascular diseases.

## 2. Methods

### 2.1. Building Putative Protein Interaction Networks for Cardiovascular Diseases

#### 2.1.1. Genes and Gene Products Related to Diseases

Proteins and mRNAs related to cardiovascular diseases were obtained using the Open Targets database (https://www.opentargets.org). Open Targets is a public-private initiative to generate evidence on the validity of therapeutic targets based on the analysis of genome-scale experiments [9]. It has recently added data sources for targeting disease associations and new disease annotations. It also contains new network analysis and visualization tools that can search up to 200 targets [10]. The key words “atherosclerosis,” “myocarditis,” “myocardial infarction,” and “myocardial ischemia” were used as search terms. Click “disease” to query related genes and generate a list of genes related to the disease.

#### 2.1.2. Proteins and Gene Lists of HBPs

A total of 435 HBPs were obtained from the appendix table of Ori et al., and this corresponded to 1,003 genes [11]. This table is a combination of a literature collation, data retrieved from the public database, and experimental data generated by affinity proteomics methods to ensure the list of proteins is as complete as possible.

#### 2.1.3. HBP Networks

We compared the list of HBP genes with known disease-related genes to obtain HBPs that may be associated with atherosclerosis, myocarditis, myocardial infarction, and myocardial ischemia, respectively. These genes were mapped using the database resource “Search Tool for the Retrieval of Interacting Genes” (STRING), in Cytoscape, to obtain the putative protein network structure diagram of each disease. The diagrams generated using the STRING plug-in had an associated probability confidence score, which is an estimate of the likelihood of describing the interaction of functional connections between two proteins. A higher score indicates a higher confidence in the association between multiple types of data.

### 2.2. Network Analysis

#### 2.2.1. Analysis of Clustering Coefficient

Cytoscape 3.7.1 is an open-source software platform that visualizes and integrates complex networks using data from any type of attribute. It can be used in a variety of fields, including bioinformatics, social network analysis, and the semantic web. It is the main software that we used to identify candidate genes. The “NetworkAnalyzer” tool in Cytoscape was used to determine the clustering coefficients of the HBP networks formed by each of the four diseases. NetworkAnalyzer is a Java plug-in for Cytoscape that computes specific parameters describing the network topology [12]. The clustering coefficient is a ratio, that is, the ratio of the number of edges between adjacent nodes to the maximum number of possible edges between them. The clustering coefficient of the network is the average of the clustering coefficients of all nodes in the network. The larger the clustering coefficient, the closer the relationship between adjacent nodes.

#### 2.2.2. Random Networks

The heparin-binding hypothetical protein networks in the four diseases were compared with eight random networks to determine whether the network generated by the assumed HBP interactions was random. Random networks were generated using the “Random Networks” plug-in in Cytoscape, which randomizes nodes to produce different clustering coefficients. The clustering coefficients of the four diseases were compared with the average clustering coefficients of the random networks. If the clustering coefficients of the putative HBP networks were significantly higher than those of the corresponding random networks, they were considered to be well connected.

### 2.3. The Search for Candidate Genes

#### 2.3.1. MCODE for Subnet Networks

The Cytoscape plug-in, MCODE, includes a classic clustering algorithm for protein complexes and has the ability to extract and visualize hot spots of interest. Parameters can be set according to the data, and thus, subnetworks of the same cluster can be created of any number and size, as needed, to identify important components of key nodes. Using this plug-in, we obtained several key subnet networks for the future analysis of gene interactions in atherosclerosis, myocarditis, myocardial infarction, and myocardial ischemia.

#### 2.3.2. CytoHubba for Hub Genes

CytoHubba is a Cytoscape plug-in that can sort nodes in a network based on network characteristics. CytoHubba provides 11 different topological analysis methods based on the shortest path, including degree, edge penetration component, maximum neighborhood component, maximum neighborhood component density, and maximum cluster centrality [13]. The advantage of the CytoHubba plug-in is that it can perform all 11 analysis methods in one operation and order the nodes according to their corresponding scores, thus synthesizing the important nodes in the biological network. The results of this topological analysis of subnets provide experimental biologists with new insights into basic regulatory networks and protein drug targets [14].

#### 2.3.3. Metascape for Heat Maps

Metascape is a functional annotation analysis tool that can help apply the currently popular bioinformatics analysis methods to the batch analysis of genes and proteins. Metascape is capable of the BioGrid-based analysis of protein-protein interactions (PPI), enabling interactive visualization of gene ontology (GO) networks and generating enrichment heat maps to enhance our understanding of gene or protein functions. Metascape is an efficient tool for biologists to comprehensively analyze and explain omics studies in the big data era [15].

## 3. Results

### 3.1. Building Putative Protein Interactomes for Atherosclerosis, Myocarditis, Myocardial Infarction, and Myocardial Ischemia

**Figure 1:**
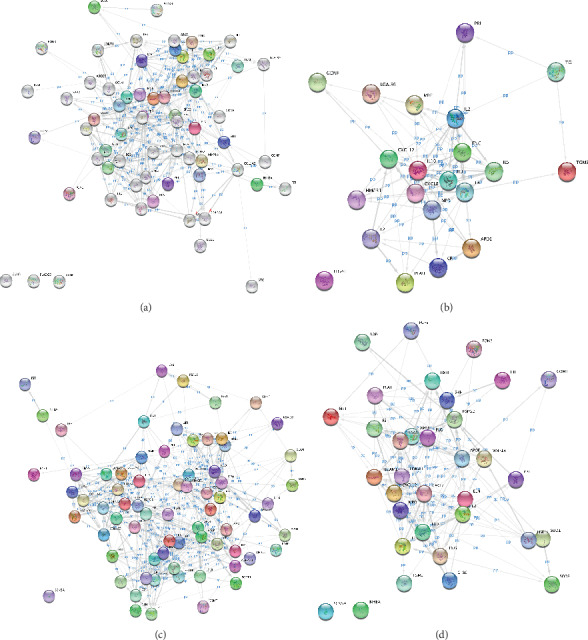
Putative HBP interactomes of atherosclerosis (a), myocarditis (b), myocardial infarction (c), and myocardial ischemia (d).

By comparing the HBP gene list obtained above with the disease-related gene list obtained from Open Targets, we found 66 HBP genes to be related to atherosclerosis, 21 to be related to myocarditis, 71 to be related to myocardial infarction, and 34 to be related to myocardial ischemia. The network generated by Cytoscape is a kind of “putative protein interaction” network because the HBP family data exported were mRNA expression data, and the HBP interaction network was retrieved from the STRING database. It remains uncertain whether the expression of these proteins in actual cultured cells or tissues corresponds to the putative interactions identified. Network analysis is used to quantify the degree to which these interactions are regulated. The list of relevant HBPs is shown in Supplementary Tables S1–S4. The assumed HBP network diagrams generated by the STRING plug-in are shown in Figures 1(a)–1(d).

### 3.2. Analysis of Clustering Coefficients

It remains to be tested whether the HBP interaction network members are more closely related to each other than those of the normal protein interactions and random HBP networks. Therefore, we calculated the clustering coefficients for the HBP interaction network of the normal heart, the HBP interaction networks of the four diseases (Ec_hepint), the non-HBP interaction network (Ec_not hepint), and the random network (Ec_hepint_random) [5] and determined the degree of network connection according to the clustering coefficients. The Ec_not hepint genes are the disease-related genes from Open Targets and do include genes from the HBP family.

**Figure 2:**
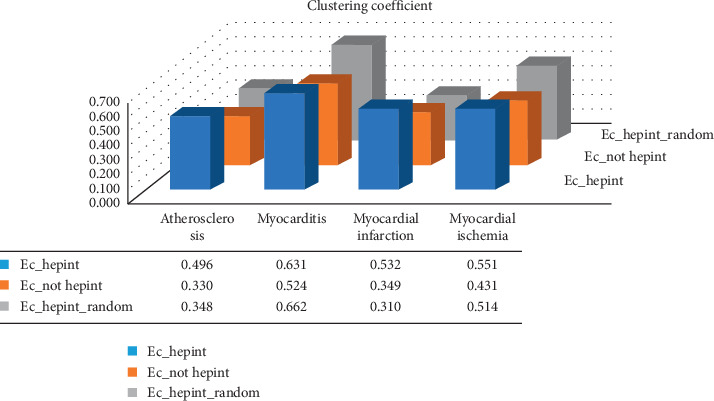
Clustering coefficients of the four different diseases.

According to “NetworkAnalyzer,” the networks in the normal heart have three connected components with highly interconnected HBPs and a clustering coefficient of 0.490. In atherosclerosis, myocarditis, myocardial infarction, and myocardial ischemia, the clustering coefficients of the putative HBP network were 0.496, 0.631, 0.532, and 0.551, respectively. These values were higher than those of the normal cardiac protein networks (Figure 2). Correspondingly, the clustering coefficients of Ec_hepint were greater than those of Ec_not hepint and Ec_hepint_random, with the exception of myocarditis (Figure 2). The clustering coefficients of each random network were normally distributed, and therefore, we averaged the eight random networks to avoid values in the low probability region. For this reason, the correlations between components of the HBP network were higher than those of the corresponding random network and the non-heparin-binding protein network. Thus, the HBP interaction networks of atherosclerosis, myocarditis, myocardial infarction, and myocardial ischemia were highly interconnected modules, indicating the presence of therapeutic targets.

### 3.3. Searching for Critical Genes of HBPs

**Figure 3:**
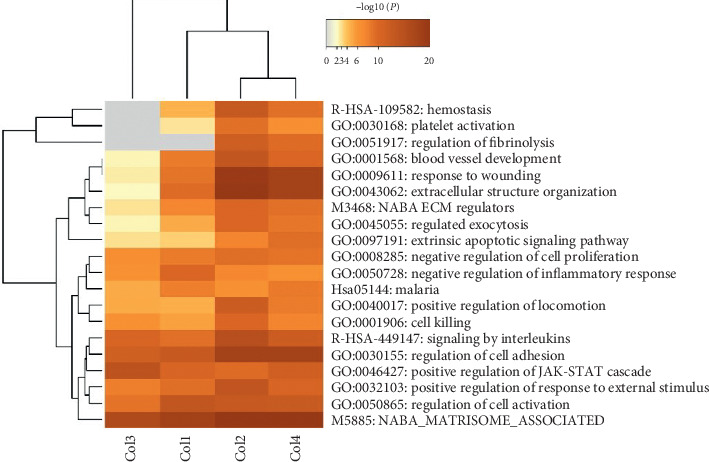
Subnet nodes in cardiovascular diseases ranked by scores.

In the protein interaction networks of each disease, there were tightly linked and loosely linked components. To further analyze the networks, we generated several subnets of the HBP network of each disease through the “MCODE” plug-in. For diseases with more than one subnet, we selected the subnet with the highest analysis score, that is, the most closely connected subnet. By performing a reverse-sequence analysis of the scores of the resulting subnets of the cardiovascular system (Figure 3), some key highly connected genes were identified, including *HP*, *CXCL12*, *COL3A1*, and *PECAM1* (Table 1).

To exemplify, *COL3A1* encodes the fibrillar collagen that is found in extensible connective tissues, such as the vascular system, frequently in association with type I collagen. Mutations in this gene are associated with Ehlers–Danlos syndrome type IV and aortic and arterial aneurysms [16]. The protein encoded by *PECAM1* is found on the surface of platelets, monocytes, and neutrophils and makes up the majority of endothelial cell intercellular junctions. The encoded protein is a member of the immunoglobulin superfamily and is likely involved in leukocyte migration, angiogenesis, and integrin activation [17]. *HP* is known as the gene of a preprotein that processed to produce haptoglobin. It binds to free plasma hemoglobin, bringing the degrading enzymes close to hemoglobin, while preventing iron loss through the kidneys. Furthermore, *CXCL12* encodes chemokine CXCL12, which has a strong chemotactic effect on lymphocytes and plays an important role in cells, including immune monitoring, inflammatory response, tissue homeostasis, tumor growth, and metastasis. These genes encode basic matrix proteins, immune proteins, and chemokines in the cardiovascular system, similar to transport hubs in protein interaction networks, and have implications for the study of cardiovascular disease targets.

### 3.4. Analysis of Hub Genes of HBPs in Each Disease

Many types of biological data can be obtained by analyzing networks, including information regarding gene regulation, signal transduction pathways, and protein-protein interactions [18]. We can determine the importance of each node in the network by how closely it is connected to the other nodes, thus enabling the identification of the central element of the biological network, namely, the hub gene.

According to the results of the CytoHubba analysis, we unexpectedly identified *IL10* as a common hub gene in atherosclerosis, myocarditis, and myocardial ischemia (Table 2). The *F2* gene was found to be the hub gene in myocardial infarction. These results highlight the possibility that if there are clinical research data to support the association of these proteins with patient survival, the levels of these specific HBP proteins may be good indicators of disease treatment progress and prognosis.

### 3.5. Investigating the Relationship between the Cardiovascular Disease Pathway and HBPs

Metascape identified all statistically enriched terms (GO/KEGG terms). Cumulative hypergeometric *p* values and enrichment factors were calculated and used for filtering. The remaining significant terms were then hierarchically clustered into a tree based on kappa-statistical similarities among the gene members. A kappa score of 0.3 was then applied as the threshold to cast the tree into term clusters. The lowest *p* value within each cluster was selected as the representative term displayed in a dendrogram. The heat map cells were colored according to their *p* values, with white cells indicating the lack of enrichment for that term in the corresponding gene list (Figure 3).

By analyzing these GO terms, the HBP-related genes were linked to the signaling pathways that the proteins interacted with. Thus, the specific mechanism of action of the HBP family may be determined from the perspective of the overall analysis. However, the specific functions of the proteins still require experimental verification.

## 4. Discussion

The extracellular matrix (ECM), a complex network of cross-linked proteins, is an important regulator of cell proliferation, survival, differentiation, and migration [19]. To explore whether HBP proteins can be used as a biomarker of cardiovascular disease, we adopted a proteomics strategy to characterize the HBP network in normal hearts and in hearts from patients with atherosclerosis, myocarditis, myocardial infarction, and myocardial ischemia by enriching and analyzing HBPs. Moreover, the bioinformatics analysis tool Cytoscape was used to predict the key disease-related genes, namely, HBPs and related factors. In the results obtained by different methods throughout our study, it can be seen that the HBP family forms a highly connected network of interactions in both normal hearts and hearts from patients with cardiovascular diseases, which, to some extent, defines the role of important protein regulatory modules in the extracellular matrix.

**Figure 4:**
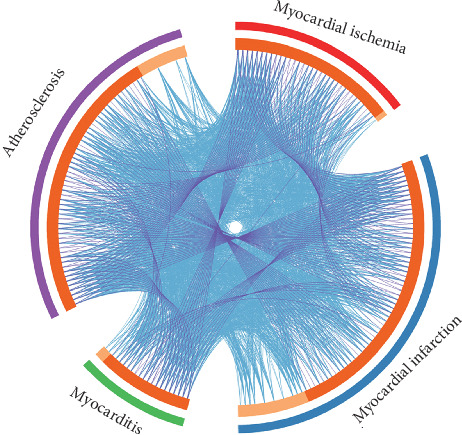
Overlap of genes from the input gene lists.

Through a comparative analysis using Metascape, we found that there were both common and independent genes related to the four heart diseases. The relationship between these genes can be clearly seen in the Circos plot (Figure 4). A greater number of purple links and longer dark orange arcs imply a greater overlap among the input gene lists. The blue links indicate the amount of functional overlap among the input gene lists. On the outside, each arc represents the identity of each gene list. On the inside, each arc represents a gene list, where each gene has a position on the arc. Dark orange color represents the genes that appear in multiple lists, and light orange color represents genes that are unique to that gene list. Purple lines link the same gene that is shared by multiple gene lists. Blue lines link the different genes that fall into the same ontology term (the term must be significantly enriched and with a size no larger than 100).

The reason for the lower number of genes in the generated map compared to the number of input genes is that genes were ignored when their degree of connection was extremely low such that the *p* value score and other indicators were too low. Little direct overlap among studies is a common observation in meta-analyses due to the variations in the biological assays used. However, substantially, more functional overlap is common as these studies are likely to have captured different parts of the same biological processes.

Historically, research on biomarkers and targeted drugs has mostly begun with clinical trials. In addition, drugs targeting a single molecule, developed after a large number of experiments, are less effective at treating the complex pathological mechanisms of inflammatory diseases and cancers. In contrast, we believe that a holistic systems biology approach, based on heparin-interacting factors, has the potential to provide definitive evidence of biomarkers and drug targets in heart-related diseases after sufficient experimental verification.

These conclusions were also supported by an annotated analysis of gene function in “Metascape” as these established cellular signaling pathways and molecular functions are consistent with atherosclerosis, myocarditis, myocardial infarction, and myocardial ischemia. For example, in the enrichment terminology analysis, the GO terms enriched in the HBPs associated with the four diseases were “M5885,” “GO: 0050865,” and “GO: 0046427.” Through the analysis of GO enrichment terms, we found that, in cardiovascular diseases, HBPs were closely related to cell activation, cell movement, cell migration, and cell adhesion (Figure 3) and showed upregulated expression in these signaling pathways [20]. Accordingly, Pagel et al. [21] reported that HBPs can increase the proliferation and viability of osteoblasts and can even promote the formation of a well-developed actin cytoskeleton. Chen et al. demonstrated that the inhibition of HBP transcription can reduce the inflammatory response and is an effective treatment against atherosclerosis [22]. Xing et al. found that the HBP activates M1 macrophages and suppresses TNF-*α* and IL-6 secretion in sepsis [4, 23]. All of these previous findings are consistent with our results. The M5885 (NABA_MATRISOME_ASSOCIATED) gene set contains genes encoding combinations of ECM-related proteins, including ECM affinity proteins, ECM regulatory factors, and secretory factors.

Based on the evidence provided by the HBPs in our disease network model, we were able to generate substantial guidance for the comparative analysis of HBPs using physiological and pathological tissue proteomics techniques.

As we identified some hub genes in the HBP family that were closely related to the development of disease, namely, *IL10* and *F2*, we may reasonably suggest that these HBP family members can be further targeted for the diagnosis and prognosis of cardiovascular diseases in clinical practice. IL10 is a typical anti-inflammatory cytokine that has obvious immunosuppressive and tissue-protective effects and inhibits the secretion of proinflammatory cytokines. In addition, it promotes B cell differentiation and proliferation and inhibits eosinophil survival and antibody-mediated eosinophil inflammation [24]. Aspelund et al. demonstrated that IL10 is increased simultaneously with proinflammatory cytokines to avoid excessive inflammation in the host organism, indicating that HBP and IL10 can be used as biomarkers to predict bronchoalveolar lavage fluid infection [25]. Meanwhile, the F2 gene encodes a protein that maintains vascular integrity. In addition, peptides extracted from the C-terminal region of the protein have antibacterial activity against *Escherichia coli* and *Pseudomonas aeruginosa* [26]. Furthermore, the key genes identified by MCODE analysis, *HP*, *CXCL12*, *COL3A1*, and *PECAM1*, may also serve as candidate targets for cardiovascular disease diagnosis.

These results imply that the HBP family members have an actual correlation with the inflammatory response and vascular integrity, but whether they are also candidate biomarkers for the diagnosis and treatment of other diseases involving inflammation and blood vessels requires further research studies to prove.

## 5. Conclusions

HBPs constitute a highly regulated extracellular subprotein group in the normal heart and in the hearts of patients with atherosclerosis, myocarditis, myocardial infarction, myocardial ischemia, and other diseases, suggesting that they can be used as biomarkers for future clinical diagnoses and treatments. Through big data analysis, we found that HBP family members, such as *HP*, *CXCL12*, *COL3A1*, *PECAM1*, and other highly correlated genes, play a role in heart diseases. Importantly, the HBP gene family members perform a common function in all four diseases, for example, they upregulate the expression of ECM affinity proteins, ECM regulators, and secretory factors. The current study demonstrates the power of system analysis of meta-expressive data, in which predetermined uncertainties and interactions do not limit the ability to generate useful predictions and recommendations.

Although the vital role of HBPs in diseases has been demonstrated clinically, there is still no strong research evidence for their role in the cardiovascular system. This paper is the first complete analysis and prediction of protein interaction networks using bioinformatics simulations. Although we still lack clinical specimens, these will be used to verify our findings through future studies. Eventually, if these simulated effects of HBP proteins are verified experimentally, appropriate treatments can be identified in advance according to different disease-related genes involved. This will have a profound influence on targeted clinical therapy.

## Figures and Tables

**Table 1:** Subnet nodes in cardiac diseases ranked by scores.

Atherosclerosis		Myocarditis		Myocardial infarction		Myocardial ischemia	
HP	3.862012	CXCL12	4.730252	COL3A1	4.227711	PECAM1	3.840842
F2	3.374677	HMGB1	4.069932	COL1A1	3.835219	PLG	3.805241
APOE	3.245926	CXCL8	3.222489	GC	3.614845	APOE	3.245926
CXCL8	3.222489	MPO	2.91836	COL1A2	3.392512	CXCL8	3.222489
VTN	3.120123	IFNG	2.68226	COL4A1	3.37931	APOB	3.119399
MPO	2.91836	IL10	2.649526	COL18A1	3.323899	MPO	2.91836
IL10	2.649526	IL2	2.179924	APOB	3.119399	MMP14	2.805141
PLAU	2.581302	IL4	2.145607	CP	2.941319	IFNG	2.68226
		IL3	1.550841	MMP14	2.805141	IL10	2.649526
		IL5	1.345461	HRG	2.367027	IL2	2.179924
				CPB2	2.131268	IL4	2.145607

**Table 2:** Hub genes in the protein-protein interaction network^*∗*^.

Rank	Atherosclerosis	Myocarditis	Myocardial infarction	Myocardial ischemia
1	IL10	IL10	F2	IL10
2	CXCL8	IL4	FGA	CXCL8
3	IL4	IFNG	FGB	PECAM1
4	IFNG	CXCL8	GC	PLG
5	IL2	IL2	HRG	APOE
6	IL3	IL5	APOB	CXCL12
7	CXCL12	IL3	FETUB	IL4
8	IL7	CXCL12	CPB2	MPO
9	PECAM1	MPO	F12	IFNG
10	IL5	HMGB1	ITIH3	F2

^*∗*^The genes were ranked by scores with CytoHubba, and the top is considered to be the hub gene of the HBP in the disease.

## Data Availability

The lists of genes of HBPs used to support the findings of this study are included within the supplementary information file.

## Supplementary Materials

Supplementary Materials S1 Table: lists of genes of HBPs, myocardial ischemia, myocardial infarction, myocarditis, and atherosclerosis; S2 Table: lists of genes of HBPs associated with myocardial ischemia, myocardial infarction, myocarditis, and atherosclerosis; S3 Table: lists of genes of non-HBPs associated with myocardial ischemia, myocardial infarction, myocarditis, and atherosclerosis; and S4 Table: lists of clustering coefficients of Ec_hepint, Ec_not hepint, and Ec_hepint_random.
