# Supplementary material for: Heparin-Binding Protein: A Novel Biomarker Linking Four Different Cardiovascular Diseases
Source: Cardiol Res Pract. 2020 Jun 15;2020:9575373. doi: 10.1155/2020/9575373 (PMC7312699; doi:10.1155/2020/9575373)
Supplement: Supplementary Materials — S1 Table: lists of genes of HBPs, myocardial ischemia, myocardial infarction, myocarditis, and atherosclerosis; S2 Table: lists of genes of HBPs associated with myocardial ischemia, myocardial infarction, myocarditis, and atherosclerosis; S3 Table: lists of genes of non-HBPs associated with myocardial ischemia, myocardial infarction, myocarditis, and atherosclerosis; and S4 Table: lists of clustering coefficients of Ec_hepint, Ec_not hepint, and Ec_hepint_random. [file 9575373.f1.pdf]

Supplementary Table 1:

Lists of genes of HBPs, myocardial Ischemia, myocardial infarction, myocarditis and atherosclerosis

HBP	Myocardial Ischemia	Myocardial infarction	Myocarditis	Atherosclerosis
1-Oct	HMGCR	PLG	ABCD1	PCSK9
A-152E5.1	SCN5A	P2RY12	ACAT2	ABCA1
A-152E5.2	CACNA1D	F2	ACE	PTGS2
A1BG	CACNA1C	NR3C2	ACE2	NDUFB9
A2M	SLC6A4	DPP4	ACHE	VDR
A2MRAP	CACNA1F	ADRB2	ACLY	PPARA
A4	CACNA1S	ACE	ACP5	PTGS1
AACT	SCN4A	TNF	ADAM17	NDUFV2
AAG4	PTGS2	HMGCR	ADAM9	ACE
AAMP	PTGS1	PPARG	ADAMTS1	HMGCR
AAT	PPARA	PPARA	ADAR	P2RY12
ABCB1	GUCY1B2	PTGS2	ADRB1	ABCG5
ABCB11	GUCY1B1	AGTR1	ADRB2	F2R
ABCB4	ACE	ITGA2B	AFP	NPC1L1
ABCC6	ADRB1	ITGB3	AGTR1	NR3C1
ABCG2	ESR1	PPARD	AHR	NR3C2
ABCG5	ESR2	ADRB1	AIM2	ABCG8
ABCP	PDE5A	SERPINC1	AKT1	CNR1
ABP1	ITGA2B	GLP1R	AKTIP	PTGIR
AD1	ITGB3	F2R	ALB	ADRB2
AD3	PDE7A	PTGS1	ALOX5	VKORC1
ADAMTS1	PDE2A	INSR	ALPK1	PDE3A
ADAMTS11	PDE6G	LRP8	ANG	NDUFC2
ADAMTS3	PDE4B	CSF3R	ANGPT1	MT-ND2
ADAMTS5	PDE3A	ADRA1D	ANPEP	GRIN2A
ADAMTS8	PDE7B	TRPV1	ANXA5	XDH
ADAMTS9	VKORC1	ADRB3	ANXA6	ADRB1
ADCP2	PDE9A	FKBP1A	APEX1	MT-ND5
ADMLX	PDE6D	SLC12A3	APLN	GPD2
ADMP2	PDE1C	GUCY1B1	APLNR	MT-ND1
ADMR	PDE3B	OPRM1	APOE	MT-ND6
ADRA1B	THRB	CACNA1D	AQP4	ADORA3
AE1	PDE6H	CACNA1C	AR	GRIN2B
AGRP	PDE6B	GABRB3	ARSA	NDUFA7
AGRT	PDE6A	GABRQ	ASGR1	NDUFS3
AGT	THRA	TUBB8	ATF4	MT-ND4L
AGTI	PDE1B	TUBB3	ATP2A2	MT-ND4
AGTIL	PDE1A	TUBB	AVP	MT-ND3
AIGF	PDE4D	TUBB4B	B2M	GRIN3A
AIMP1	PDE8B	GABRD	BAG3	NDUFA4
AKR1B1	SLC29A1	KCNK18	BCHE	NDUFA13

ALDR1	PDE10A	GABRA5	BCKDK	NDUFA4L2
AMBP	PDE4C	GABRG3	BCL2L12	NDUFA6
ANG	PDE6C	TUBB6	BCL6	NDUFA12
ANX1	PDE8A	PDE3A	BMP2	NDUFB1
ANX2	PDE4A	KCNK3	BMPR2	NDUFAF3
ANX2L4	FGF21	ADRA1B	BTG3	GRIN1
ANX3	TFPI	KCNK9	C3	NDUFA11
ANX5	GSK3B	HTR3A	C5	PAH
ANX6	MPO	HTR4	C5orf38	NDUFA3
ANXA1	AREG	GABRB1	CA4	ADORA2B
ANXA2	CIRBP	GABRG1	CABIN1	NDUFS5
ANXA3	TNFRSF11B	PTGER1	CADM1	NDUFV1
ANXA5	ALDH2	GABRA2	CALCR	NDUFB8
ANXA6	SAA1	DRD2	CALU	NDUFB6
AOC1	CSF3	GLRA1	CASP1	NDUFS4
APCS	PIK3CG	GABRB2	CASP3	NDUFAF2
APLP1	EPAS1	GABRA6	CASP8	ADORA1
APLP2	HIF1A	TUBB2B	CASP9	GRIN2C
APOA5	TLR2	TUBB2A	CAST	NDUFV3
APOB	TLR4	PTGER2	CAT	NDUFS2
APOE	HMGB1	GUCY1B2	CAV1	NDUFB11
APOH	FADD	ADRA1A	CCL11	NDUFS6
APOJ	FABP3	FAAH	CCL2	NDUFB10
APP	PTEN	GABRG2	CCL20	NDUFA9
APPL2	UNC5B	GABRA4	CCL26	NDUFAF1
APRIL	HMOX1	TUBB4A	CCL27	NDUFB5
AQP1	FGF2	GABRE	CCL3	NDUFA2
ARA	HGF	CACNA1F	CCL4	NDUFA10
ARG1	SELP	TUBB1	CCL5	NDUFA5
ART	GJA1	KCNK10	CCL7	ADORA2A
ARTN	VEGFA	GABRP	CCN1	F10
ASIP	NOS3	KCNK2	CCN2	NDUFA1
ASP	TNF	CACNA1S	CCR1	NDUFAF4
AT3	DPP4	GABRA1	CCR2	NDUFA8
ATA3	LEP	NPC1L1	CCR3	NDUFB3
ATP1A1	MB	GABRA3	CCR4	GRIN3B
ATP1B	CRP	PPIA	CCR5	NDUFS7
ATP1B1	IL10	F10	CCR7	NDUFS8
ATP1B3	PPARG	EPOR	CCR8	NDUFC1
ATP2B1	AGTR2	ADORA2B	CD14	GRIN2D
ATP5A	FGFR1	ADORA3	CD163	NDUFB7
ATP5A1	MMP1	ADORA1	CD19	NDUFB2
ATP5AL2	CXCL12	ADORA2A	CD2	NDUFB4
ATP7B	TNNI3	VKORC1	CD209	NDUFS1
ATPM	RYR2	CELSR2	CD27	NDUFAB1
ATRC2	HRH3	PHACTR1	CD274	TUBB4B
ATRN	CXCR4	IL1R1	CD276	TUBB8

AZU1	PLA2G1B	GUCY1A1	CD28	TUBB3
B2G1	UCN3	REN	CD34	TUBB
B2M	EDN1	CDKN2B	CD38	TUBB6
BACE	APLN	TNNI3	CD3D	TUBB2B
BACE1	CASP3	TNNT2	CD4	TUBB2A
BAL	CCL2	ESR1	CD40	TUBB4A
BCA1	INS	ESR2	CD40LG	TUBB1
BCRP	OLR1	MME	CD44	PLA2G7
BCRP1	PCSK5	C5	CD55	IL1B
BDK	ST3GAL4	VDR	CD68	PDE5A
BEK	EPHB2	THRA	CD69	PPARG
BF	NLRP3	PDE3B	CD70	AGTR1
BFD	STAT3	THRB	CD72	KCNJ11
BGN	TXN	SLC5A2	CD80	ABCC8
BLC	TGFB1	TNNC1	CD83	EDNRA
BMP2	SLC33A1	SLC12A1	CD86	PIK3CG
BMP2A	TNNT2	LPA	CDK9	IPO5
BMP2B	REN	MEF2A	CDKN2A	ZHX2
BMP3	SOAT1	BRAP	CEACAM5	LDLR
BMP3A	PLAT	MIA3	CEBPA	PINX1
BMP4	TET1	KCNE2	CEBPB	PINX1
BMP6	MMP9	LIPA	CENPA	HDAC9
BMP7	CXCL8	CCT7	CFD	SLC17A4
BP2	BECN1	CLCN1	CFLAR	APOE
BRI	PARP1	RNF13	CFP	ESR1
BRI2	JAK2	SMARCA4	CGAS	FGF21
BSEP	SIRT3	ATXN2	CHST15	ATXN2
BTC	TAB1	PSRC1	CHUK	MAPK10
BWR1A	TMSB4X	PDZD2	CIITA	DKK3
BWSCR1A	SELE	IRX1	CISH	CSMD3
C10orf13	TRPV1	C10orf142	CLU	MAPK14
C10orf58	ICAM1	MAT2A	CMA1	CX3CL1
C1IN	BSG	PLPP3	CNOT7	SCARB1
C1NH	ZGLP1	PLCL2	CNP	REN
C1QA	SOD1	AP3D1	CNR1	NLRP3
C1QB	VPS51	MORF4L1	CNR2	CD40
C1QC	ALB	JCAD	CORT	ANGPTL4
C1QG	MTOR	ATP2B1	CP	MAPK11
C2	IL6	WDR12	CPE	CCR2
C20orf182	NPPA	IL6R	CR1	ALOX5
C3	GRK2	YTHDF3	CR2	LRIG1
C4A	NOS2	PCSK9	CRP	SIRT1
C4BP	HTRA2	ZC3HC1	CSDE1	ACE2
C4BPA	S100A6	SMAD3	CSF1	OSBPL10
C4orf31	IL18	COL4A2	CSF1R	ANGPTL8
C5	EPO	APOC1	CSF2	LPA
C6	IFNG	FES	CSF3	IL32

C6orf15	MMP2	SERPINA5	CSRP3	PGR
C6orf25	ITGB2	TENM3	CTF1	ADAMTS4
C7	FGF1	DDI1	CTH	TLR4
C8A	CYCS	SPECC1L	CTLA4	CSF2RA
C8B	NOD2	ZNF536	CTSB	SORBS3
C8G	TFF3	QKI	CTSK	IL37
C9	SELL	EIF4A3	CX3CL1	CSF3
CACH1	CD34	CEACAM6	CX3CR1	LRRTM4
CACN1	AQP1	SAYSD1	CXADR	APOA1
CACNA1S	FXYD1	DHFR	CXCL1	CCN3
CACNL1A3	CAST	DGKB	CXCL10	CNR2
CAL1H	SERPINE1	COL4A1	CXCL12	ALG1
CAT2	P2RX3	SMG6	CXCL13	MAP4K4
CBG	DSE	VEGFA	CXCL8	TREM1
CCC6	ATF6	NPR1	CXCL9	CCDC57
CCDC134	ADAMTS13	DCLK2	CXCR3	CDKN1A
CCDC80	TNC	PRKCD	CXCR5	IGLL1
CCL1	CX3CL1	EDNRA	CYBB	IL6
CCL11	SLC2A1	HHIPL1	CYCS	TNFRSF9
CCL13	MAPK3	BCAS3	CYP11B2	INS
CCL15	TAGLN	CXCR4	DDIT3	CD4
CCL17	EDNRB	MMP1	DDX53	MAP6
CCL19	VDAC1	SELP	DES	RARRES2
CCL2	NOC2L	MMP8	DHX58	OLR1
CCL21	AVPR2	MMP7	DLD	OSMR
CCL22	TLX2	NR3C1	DMD	MACF1
CCL23	P2RX7	OXTR	DNTT	RBP4
CCL24	ITLN1	SLC6A4	DSP	PDCD4
CCL25	F2R	TRIB1	DYSF	CCL2
CCL27	PER2	MMP13	EBI3	STAT3
CCL28	SMC2	ADAMTS7	EDN1	HAMP
CCL3	FCER1G	TRPA1	EDNRA	TRPA1
CCL4	COMT	TTC32	EIF2AK2	SERP2
CCL5	CD180	GP6	EIF4E	WWOX
CCL7	SIRT1	GIP	EIF4EBP1	CORT
CCL8	VWF	CCN2	EMB	PPARGC1A
CCN1	NGF	ACADS	EMP1	ELN
CCN2	ANXA5	FOXL1	EPAS1	TSLP
CD11B	S100A12	ZEB2	EPHA3	TLR7
CD1D	UCN	CSF3	EPHB2	TNFRSF13C
CD26	ASIC2	IL1F10	EPO	ANPEP
CD36	CASP1	MMP12	ESR1	IFNG
CD45	SSB	PAPPA2	ESR2	IKBKB
CD47	F3	SEC24D	F2	TNFSF13B
CDA08	CMA1	APLN	F2R	PAQR8
CDABP0092	CAT	TNFRSF11B	F2RL1	NES
CDH8	PHLPP1	SERPINE1	F3	KLF4

CEL	IGF1	LTA	F8	TFEB
CFB	CNP	ALDH2	FASLG	VWF
CFD	APLNR	KITLG	FGF2	BSG
CFH	FGF4	LGALS3	FGF23	BMP8A
CFI	ADRB2	MSC	FGL2	C10orf113
CFP	GLP1R	UCP3	FIP1L1	ABCA7
CGI-55	ALOX5	FN1	FLNA	CD14
CHIP28	CHI3L1	APOA1	FLT3LG	GPER1
CHRD	XPR1	TIMP1	FN1	FEZ1
CIP1	FN1	CYP2C19	FOS	SELE
CLG4A	DNM1L	MPI	FOXP3	CD47
CLG4B	IL1A	TGFB1	FUT4	NSMCE1
CLI	ANGPT1	LCN2	FUT7	NOS3
CLU	MAP3K5	TXN	FYN	LYST
CMA1	GOT2	NGF	G6PD	SAA1
CO4	CXCR2	CD14	GAPDH	NAMPT
COL11A1	PRH1	GH1	GBP1	ZYG11B
COL11A2	SETD2	FABP3	GDNF	TNF
COL12A1	CNR2	HIF1A	GH1	EGFR
COL12A1L	FABP4	CETP	GJA1	TLR10
COL13A1	VEGFC	DES	GJC1	NEGR1
COL14A1	VEGFB	RARRES2	GLB1	LCN2
COL18A1	SLC9A1	PIK3CG	GOLGA2	PLEKHO1
COL19A1	PGAM5	GCH1	GP5	PTEN
COL1A1	ASIC3	IL6	GPI	ZC3H12A
COL1A2	IL17A	PPARGC1A	GPX1	MTHFD1L
COL23A1	HDAC4	AHSG	GSK3B	CNOT4
COL25A1	MBL2	S100A1	GZMB	TGFB1
COL2A1	TXNIP	CCR7	HAMP	CXCR4
COL3A1	APOE	PIK3CD	HBEGF	MC1R
COL4A1	TTN	ATM	HDAC11	CNNM1
COL4A2	MSC	APOE	HDAC2	PIAS3
COL5A1	SERPINA4	MMP9	HECA	EVPL
COL5A3	SERPINA5	VCAM1	HGF	UTRN
COL6A3	CTSL	PPIF	HIF1A	EFHC1
COL9A1	TYMP	HDAC4	HIST1H4L	MSC
COLEC1	IL1B	INS	HLA-DQB1	LINC00305
COLL6	OSM	VPS51	HMGB1	HMOX1
COLQ	CSF2	MYOCD	HMGCR	PDZD8
COMP	KLK1	ADAMTS13	HMGXB3	PLA2G1B
COMT	NF2	FGF23	HMOX1	SELPLG
CP	PLD1	PAPPA	HNF1A	TLR2
CPAMD1	FASLG	ENG	HPRT1	PLTP
CPAMD2	UCP2	HGF	HRH1	PTH
CPAMD4	LOX	ST13	HSF1	TRAF6
CPAMD5	S100A1	IL33	HSP90B1	NAV2
CPB2	CCN2	GDF15	HSPA5	PNKD

CPD	VASP	PLA2G1B	HTR3A	HMGB1
CPSB	CASC1	PTEN	HTT	RBFOX1
CR3A	SERPINA1	GHRH	HUNK	NR1I2
CRACM1	LPL	TNFSF11	ICAM1	SIRT6
CRISP11	RIPK1	NOS3	ICOS	VIT
CRISPLD2	NOS1	CCL2	ICOSLG	GABRG2
CSF2	SERPING1	CST3	IDO1	RGL1
CTAP3	CAV1	DRAM2	IDS	MPO
CTGF	AKR1B1	LIPG	IFIH1	TANK
CTSB	PPARGC1A	CD34	IFNA1	DUSP1
CTSG	RACK1	GJA1	IFNA2	TGFB2
CX32	AQP4	TLR4	IFNA6	SELP
CX3CL1	MIF	GPI	IFNB1	RETN
CXCL1	KITLG	CRP	IFNG	RILPL2
CXCL10	EIF5A	ATF3	IFNL2	CD36
CXCL11	SMAD3	IDO1	IL10	DST
CXCL12	TRPA1	CYP2J2	IL12RB1	IL13
CXCL12	PF4	ISL1	IL13	TRAM2
CXCL13	CAV3	LEP	IL15	BRCA1
CXCL16	TLR9	HMGB1	IL17A	CETP
CXCL2	CTSD	IGFBP4	IL17D	NTM
CXCL4	HPGDS	SESN2	IL17RA	ACSM3
CXCL4V1	IL33	MBL2	IL18	ASAH2
CXCL6	NPY	MB	IL18BP	CHI3L1
CXCL7	RIPK3	RXRA	IL1A	PPP4R2
CXCL8	AGTR1	ENHO	IL1B	DNMT1
CYC	PI3	FGF2	IL1R1	PKN2
CYCS	IRF9	SOD3	IL1RN	CLU
CYH	ABCC8	SMAD9	IL2	TNIK
CYM	RETN	MPO	IL21	UBAC2
CYPB	CYBB	VWF	IL21R	PPP2R2C
CYR61	LDLR	HMOX1	IL22	ACTL7B
D17S136E	SERPINA12	CD4	IL3	CX3CR1
DAO1	SENP3	CXCL12	IL32	HSD11B1
DCC	KL	CXCL8	IL33	VLDLR
DF	CPB1	SERPINA12	IL37	TNFRSF11B
DHRS8	BNIP3	SDC1	IL4	SMAD3
DI	BDNF	PF4	IL5	ADIPOQ
DPP4	MTHFR	IL10	IL6	TRIM5
DRO1	GP6	CYP2C9	IL9	OPN4
DTR	SLCO6A1	NES	ILK	GOT1
DTS	PTAFR	MTHFR	INS	CNTN3
DUTT1	ANXA1	C3AR1	IRAK1	CXCL12
DVR4	STK11	MFAP4	IRAK4	PTPN1
ECE1	APOA1	MMP3	IRF1	BGLAP
ECM2	NAMPT	CP	IRF3	IL17A
EFL2	HYOU1	CMA1	IRF4	NCAM2

EFNA1	LY86	POSTN	IRF7	ST3GAL4
EFNA3	AKT1	APOH	IRF9	PON1
EFNA5	ENTPD1	SIRT3	ISG15	LRP1
ELA2	MRC1	NR1H3	ITGA1	PCDH8
ELANE	CREG1	PRH1	ITGA6	SLC16A3
ELC	GDF11	HAMP	ITK	FPR2
ELN	ACE2	OSM	IVNS1ABP	MACROD2
EMAP2	SERPINF1	TFPI	JAK1	HABP2
ENO1	CD40LG	UCN3	JAK2	MBTPS1
ENO1L1	EPOR	CXCL16	JAK3	HSPG2
ENPP1	IFI44	F3	JUN	CCDC7
ENPP3	KCNH2	GSTM1	KCNN4	DLGAP2
ENX2	P2RY12	CLU	KLF2	MCTP1
EPB3	ILF2	STC2	KLKB1	NBEA
EPLG1	TXN2	SFRP2	KLRD1	IL16
EPLG3	AVP	ACE2	KLRK1	SLC39A12
EPLG7	PTPRC	AGT	KMO	PELI2
ERBA2L	NTRK1	SIRT1	LAG3	EPB41L4B
ERBB2	P2RX4	IL1B	LCN2	PRKD1
ERP5	TGFB3	HSPB3	LDB3	GLO1
EVN	EEF2	ZGLP1	LDHA	PLAU
F10	AGT	MIF	LEP	PDGFRB
F11	ADM2	NAMPT	LEPR	CD34
F12	HSPD1	PON1	LGALS1	GDF15
F2	SRY	CX3CL1	LGALS3	CD55
F8VWF	SCG2	FGF21	LGALS9	MTOR
F9	WDR26	ARSA	LRPAP1	CD160
FAM55A	HDAC5	TXNL1	LTA	AHSG
FBLN7	FAP	RETN	LY6E	CACNA1C
FBN	SPP1	PINK1	MAEA	NR1H2
FBN1	PPIF	HPR	MALT1	NR4A1
FBN2	PBK	HTRA2	MAPK3	MMP9
FBRS	GZMK	IL37	MATN1	TFPI
FBS	OPHN1	TNC	MB	TAC1
FBS1	VCL	EPO	MBL2	MSANTD3-TMEFF1
FCGRT	LTA	SLC17A5	MBP	TMEFF1
FCRN	PGF	RAPGEF5	MBTPS1	LRBA
FEEL2	PLIN5	PECAM1	MCIDAS	TAF3
FELL	STEAP4	BDNF	MDK	VWA8
FETUB	TFEB	EDN1	MEFV	KERA
FEX2	NFKBIA	CSF2	MIF	NOD2
FGA	MSTN	MMP2	MLYCD	FBP2
FGB	SERPINF2	NOS2	MMP1	OGG1
FGF1	CXCL1	CD151	MMP12	LEP
FGF10	NPR2	ABO	MMP2	EPC2
FGF12	IL6ST	FLRT2	MMP3	SERPINF1

FGF12B	ATF4	PRL	MMP8	LCAT
FGF14	C5AR1	CORIN	MMP9	GRM3
FGF16	PECAM1	SERPINF1	MMRN1	TSHZ3
FGF17	MAPKAPK2	AGTR2	MMUT	PYGB
FGF18	MMP14	GDA	MOG	TXNDC16
FGF2	EIF2S1	UTS2	MPG	PFN1
FGF20	ABO	CILP	MPI	PTPRC
FGF22	VCAM1	GJA5	MPO	ICAM1
FGF3	LIPC	SCARB1	MS4A1	CDKN2A
FGF4	EGLN1	TLR3	MSC	OXR1
FGF5	ARSA	ATXN7L3B	MSN	PTPRG
FGF6	PRDX2	CCR2	MTOR	IMPA2
FGF7	TNFAIP8L2	ELN	MYH6	AKAP12
FGF8	ACR	BSG	MYH7	ITPR2
FGF9	CR1	CHGA	MYL3	GABRA5
FGFA	ZC3H12A	ADM2	MYLK	GABRG3
FGFB	LGALS3	NLRP3	MYOM2	GABRG1
FGFBP	MFN2	CCS	NCAM1	CACNA1D
FGFBP1	HTR2A	SLC2A4	NCR1	GABRA2
FGFBP3	TLR3	TFF3	NELFCD	CSF3R
FGFBR	MMP3	CHIC2	NFAT5	CACNA1F
FGFR1	BRD1	SPP1	NGF	CACNA1S
FGFR2	GRAP2	TNFSF4	NLRP3	GABRA1
FGFR3	NR1H3	IL17A	NMT1	GABRA3
FGFR4	PPARD	PLAT	NOD2	IL33
FGG	NPPC	IGF1	NOS2	HNF4A
FHF1	MT-RNR2	AQP1	NOS3	XRCC4
FHF4	NLRP1	MMD	NPM1	SPOPL
FKN	HSP90AB1	MICA	NPPA	TRIM38
FLG	BMP2	THBD	NPPC	NOXA1
FLK1	CTNNB1	PDZK1	NR3C1	FOXP1
FLT	CLEC7A	GSTA1	NR3C2	VCAM1
FLT1	CASP8	PCYT1A	NTF4	IRGM
FLT2	PIK3CB	GPER1	OGN	SOCS1
FN	PAPPA	STAT3	P2RX7	SPATA13
FN1	GLRX3	ALCAM	PCNA	ABCE1
FNRA	SLC2A4	ANXA5	PDCD1	FRMD4A
FNRB	HSPA12B	VASP	PDCD4	STXBP5L
FRP	LCN2	DBH	PDGFC	AHI1
FRP	JUN	CTF1	PDGFRA	RIMS1
FRP1	PRDX5	MTOR	PDLIM7	PLAUR
FRT	S1PR3	B2M	PI3	HSH2D
FST	OPA1	IL18	PIK3CG	CCL22
FSTL1	HRH2	ICAM1	PKP2	PAPPA
FUR	MEFV	PIK3CA	PLA2G1B	IL1A
FURIN	S1PR2	APEX1	PLAU	NPY
FWP007	EGR1	NPPA	PLP1	GPR137C

G0S19-1	CRYAB	AIDA	PNKD	IQSEC1
G17	KRT1	HTR2A	POR	COG6
G6B	HTR3A	CHI3L1	POSTN	HPCAL1
GC	NDRG2	CREM	PPARA	CCL5
GCP2	ABCA1	CAT	PPARG	P2RY6
GDNF	AGGF1	HP	PPIA	ENG
GGF	JAK1	ETV2	PPP1R15A	CD40LG
GHR	DTNA	SAA1	PPP5C	NFAT5
GIG1	CEBPZ	HFE	PRH1	FGF23
GIG24	NLRX1	TRPV2	PRKCA	MIR6792
GIG25	HSPA8	PSMA6	PRKCB	XAB2
GIG29	TIMP3	FURIN	PRL	CAMSAP3
GJB1	CFTR	S100A12	PROM1	TANC2
GLIF	ANPEP	SELE	PRSS2	CISD1
GLUT2	DNASE1	KDR	PRYP4	RGN
GLVR2	GATA4	MVD	PSMB8	SORT1
GMCSF	F2RL1	INSRR	PSME1	NTN1
GMRP	MBTPS1	GOT2	PSME2	MAF
GOK	CXCR6	TXNIP	PTEN	LUZP2
GP3A	HMGB2	SMAD7	PTGS2	SOST
GP3B	MAPK8	VEGFB	PTPRC	ARID5B
GP4	NOD1	SLC19A1	PVR	AP5Z1
GPNMB	MAMLD1	ALOX5	RAG1	MUTYH
GPR182	PARK7	IL6ST	RAPGEF5	CA6
GPR48	SLC22A4	ADA	RBM38	STC2
GRMP	SOX7	IGFBP1	REN	APOC3
GRO	MAPK7	CYCS	RHD	TET2
GRO1	ALOX15	SULT1E1	RIPK1	PF4
GRO2	ENPEP	CDK9	RIPK3	IL6ST
GROA	RAMP2	KIF6	RNASE3	NF2
GROB	MICU1	PCNA	RNPC3	CRISP2
GSN	GLA	GSK3B	RPL17	SFTPD
HARE	MAPK1	F12	RPTOR	UTS2
HBE245	TRPM7	CASP3	RYR2	DKK1
HBEGF	MAVS	MUC16	S100A8	APOM
HBNF1	CETP	ERCC8	S100A9	CMKLR1
HBP17	POR	MDK	SARS	CD59
HCF2	BCL2A1	LDLR	SCARA3	SLC17A3
HCP	SLC6A2	GRK2	SCD	PLPP2
HCP1	CNPY2	BCL2A1	SELE	P2RX7
HCS24	HSPB2	CPB2	SELL	SPP1
HDCMA22P	CCR2	S100A6	SEMA4A	MMP12
HDGF	TRPM6	SPARC	SEMA7A	MAS1
HEGFL	RIPOR2	PTH	SERPINA5	BGN
HELAD1	HSPB1	ADAM8	SERPINC1	MBL2
HER2	KIF2A	EDNRB	SERPINE1	CST3
HET	MAP2K3	SLC33A1	SGCD	TLR9

HF	GAPDH	CD36	SLC17A5	CPEB2
HF1	POSTN	ALOX5AP	SLC25A10	C6orf118
HF2	PTPN1	MMP19	SLC25A4	ABO
HFE	C5orf38	SUV39H1	SLC33A1	SERPINA12
HGF	YES1	CD59	SLC7A11	PDZK1
HGFIN	ATG3	APOC3	SMAD2	SOAT2
HGL	SIRT5	CFTR	SMAD3	TRIB3
HLAH	NUDT1	ITLN1	SOAT1	LGALS3
HMFN0376	IKBKB	SERPINA1	SOCS1	ZGLP1
HMG1	PLA2G10	THBS4	SOCS3	FABP4
HMG1L2	AHSP	ELANE	SORBS1	ANGPTL2
HMGB1	SPRR3	APP	SPARC	S1PR1
HP	ATF3	DNASE1	SPP1	MCIDAS
HPTA	RIT1	DCN	SPX	SOD2
HRG	OGG1	ADIPOQ	SRSF2	PHACTR1
HRGA	CD40	CD40LG	SSB	AOC3
HSD17B11	CCS	AOC1	SST	KCNJ3
HSD17B12	PCSK7	CDKN2B-AS1	STAT1	PDYN
HSD17B13	DLL1	AZU1	STAT2	CXCL16
HSD17B7	PADI4	MMP14	STAT3	IL10
HSMBPC	NOX1	TP53INP2	STAT4	STAT1
HSPG2	CASP9	PLAU	STAT6	CD44
HST	MME	ANG	SULT1E1	EDA
HST2	PPIA	GLB1	TACR1	FABP3
HSTF1	NGB	TMSB4X	TACR3	EDN1
HSTF2	FDPS	COMT	TBCA	ELANE
HTGL	STOM	ADAM17	TDP2	CBL
HXB	PLA2G7	APOA5	TFPI	ANXA7
HXBL	SMAD4	MBTPS1	TG	GSTM1
IAPP	HTR1B	GPR4	TGFB1	F11R
IBP2	NR1H2	TIMP3	TGM2	MAPK8
IBP3	CDK2	MAPK3	THBS1	IL5
IBP4	AHR	POR	THBS2	CCN1
IBP5	GSDMD	ESM1	TICAM1	CYP1B1
IBP6	MSLN	NFKBIL1	TIMP1	IL18
IF	CTSG	CCN1	TLR2	LRP6
IFNB2	ACHE	CPB1	TLR3	FGF2
IFNG	KCNQ1	GJA4	TLR4	IRF5
IGDCC1	MMRN1	ABCB1	TLR7	AGTR2
IGFBP10	ADRB3	CTSD	TLR9	CASP1
IGFBP2	P2RX2	ALB	TMPRSS2	LECT2
IGFBP3	KLKB1	PDGFRA	TNC	TRPV1
IGFBP4	CYSLTR2	GSN	TNF	TIGIT
IGFBP5	FAM168B	PTPRC	TNFAIP3	IL4
IGFBP6	EDNRA	TTR	TNFRSF11B	DPP4
IGFBP8	NOX4	CFH	TNFRSF14	APOB
IHH	PNPLA2	SLC9A1	TNFRSF18	ADAMTS5

IIGP5	PNOC	CRLS1	TNFRSF1A	ENHO
IL10	STAT6	NTN1	TNFRSF1B	TRPC3
IL12B	STAT5A	S100A8	TNFRSF25	EFNA5
IL2	SGK1	LGALS1	TNFRSF8	ANO3
IL3	KARS	S100B	TNFSF11	NUTM2F
IL4	LPA	BGN	TNFSF4	ERICH1
IL5	TXNL1	PARP1	TNFSF8	RCAN1
IL6	SULT1E1	CA3	TNFSF9	SOCS3
IL7	VIP	CTSS	TNNI3	UMOD
IL8	CDK9	SCUBE1	TNNT2	CRP
ILC	GAST	RNASE3	TOR1A	SMAD2
IMPG2	CPA1	ACSM3	TRAF6	TLR5
IMPT1	SOCS3	HSPB1	TREX1	HGF
INHBA	ATP5IF1	LPL	TRIM21	SLC10A2
INP10	KCNMA1	SERPINF2	TRIM63	SEMA4D
INSR	CPOX	SOAT1	TSPO	FMO3
INT1	TCP1	IFNG	TTN	MTHFR
INT2	ST8SIA4	ITGA2	TTR	NR1H4
IPM200	SLC17A5	FASLG	TXN	MMP10
IRGC	SCD	OLR1	UCHL1	VEGFA
IRGC1	HDGF	SCN5A	VCAM1	ZEB1
ITAC	GPX1	BAG3	VCL	SAT1
ITFG1	CTH	TLR2	VDR	MMP2
ITGA1	PXDN	CTNNB1	VEGFA	MMP8
ITGA5	NCL	ADM	VIM	PRKAA1
ITGAM	SOD3	GNB3	VIP	KL
ITGAV	EFNA1	THBS1	XCL1	PRKCB
ITGB1	TRIM63	PTGIS	XPR1	FAM49A
ITGB3	CA1	CHAT	ZBTB12	ACER1
ITIH3	CALR	FGF1	ZC3H12A	SHH
ITIL	HDAC6	GAP43	ZC3HAV1	ISOC1
ITM	BCLAF1	AOPEP		TNFSF11
ITM2B	PON1	DMD		ALB
JTK2	NKX2-5	BRD1		HMBS
JTK4	SLN	SCD		GPR132
KAL	HPS1	SLC5A5		ADARB2
KAL1	CD151	KLK1		PPARD
KALIG1	DDAH1	F7		SERPINE1
KDR	NFAT5	CCNA2		DCN
KGF	CASR	KLF4		BDNF
KGFLP1	FGF23	GUSB		ATF3
KGFLP2	DAXX	IL18BP		GJA1
KGFR	ARC	TIMP2		ABCG1
KIAA0253	DYSF	CASP1		APOC1
KIAA0366	MASP2	ITGB2		TPT1
KIAA0533	GPI	LEPR		PADI4
KIAA0548	IGFBP4	CYP4F2		TIMP1

KIAA0786	SLPI	IL2		GPX1
KIAA0813	CLU	RYR1		PON2
KIAA1149	SLC5A5	PGF		HSPD1
KIAA1312	TNFRSF1A	HMBS		DLK1
KIAA1346	HSPA5	NOS1		FAM174A
KIAA1419	IGF2	PIM1		SETBP1
KIAA1445	FSTL1	CYP19A1		ZFHX4
KIAA1907	CYP2E1	RIPK3		S100A9
KNG	TICAM1	MRTFA		FABP5
KNG1	PIAS1	CADM1		CD69
KS3	EMP1	MGP		IL17D
KSAM	EPRS	ADAMTS1		TES
KUB1	IL2	OGN		IL2
LACI	KDR	LIPC		CSF2
LAG1	EMD	MRC1		QDPR
LAMA	PRL	APOB		MCPH1
LAMA1	AHSG	ABCC9		CCR5
LAMA2	DIAPH1	CKLF		MAPK7
LAMA3	SLC25A1	ANGPT1		PHLDA1
LAMA4	MPI	BCHE		PLIN2
LAMA5	NR1H4	SFRP5		CTLA4
LAMB2T	IRAK1	DPT		POMC
LAMBR	TNFRSF1B	A2M		VIM
LAMC2	DES	NR1H2		NOTCH1
LAMM	HAMP	SDC4		CXCR6
LAMNA	DNAH5	ALOX15		NPPA
LAMNB2	PBRM1	GSTP1		FN1
LAMR1	SELENOP	KLKB1		SREBF1
LCRISP2	P2RY11	TREM1		HIF1A
LDLR	DST	SOD2		RASEF
LEC1	APAF1	HSPD1		TRPS1
LERK1	PRDX1	LAG3		HIVEP2
LERK3	GPER1	ABCA1		FOLR2
LERK7	GDF15	PFN1		SPON2
LF	CCL5	FLT1		MMP1
LGALS9	TIMP1	CCL11		PARP1
LGR4	F2	CYP11B2		CMA1
LIFR	HSPB3	LRP1		MFGE8
LIPC	ANG	GP1BA		TNC
LIPD	SUCNR1	PI3		TIMP3
LIPG	AKIP1	LPP		JAM3
LIS1	SMPD1	S100A4		HHIP
LNHR	CFB	PRKCA		INF2
LNPEP	RNLS	BRINP3		KIF20B
LPC1	DDIT3	HSPG2		GLRX3
LPC2D	PCBD1	TNFRSF1A		LIPG
LPHH1	CDS1	CCR5		ARHGEF26

LPHN2	GHRL	IL11		APOD
LPL	PLG	HSPB8		IRS2
LRPAP1	FGFR3	NEIL3		SMPD3
LST3	NTS	RFLNA		OVOL2
LTBP1	NFE2L2	MAZ		CDH10
LTF	PSMA2	MAPKAPK5		AIF1
LTN	CCR5	TF		IGF1
LYAM1	SESN2	F8		GJB5
M6S1	SULT4A1	CYBA		MMRN2
MAPT	PKN1	CAST		MMP16
MAPTL	TPO	PDE5A		NPVF
MBL	MAP2K1	DEGS1		CBLN1
MBL2	OPN1LW	GHSR		ADAM8
MBP	LPO	PLA2G4A		IGF2
MBPB1	SELPLG	LGALS2		APOA4
MCP1	IRAK4	ENPEP		APP
MCP2	MRTFA	IL4		PON3
MCP3	F8	NPY		CFP
MCP4	CTSB	CYP1A2		LIPC
MCT1	FGF19	OGA		ST13
MCT8	SKIL	CNR2		LTA
MDC	ANO1	HRG		DGAT1
MDCR	NDRG4	CENPJ		FOXP3
MDF2	KLF5	F2RL1		VEGFC
MDK	TSPO	HPGDS		ALOX15
MDR1	PVR	CCDC88A		RYR3
MDR3	UCP3	ATP2A2		LPAR1
MDS	NUP43	C4B		RHOF
MDU1	OGA	PROCR		TNFRSF1A
MEGF4	S100A4	AQP4		ESR2
MER6	NTRK2	CD40		ADAM17
MET	CCN1	FABP2		NISCH
METH1	FGF5	ADD1		SCD
METH2	CASZ1	ANXA1		VNN1
MGCA	CDKN1A	IRF3		GJA5
MGSA	HIF3A	TIMP4		COG2
MIF	TNNI3K	LBP		CD163
MIP1A	STAT1	CX3CR1		LIPE
MIP1B	VDR	UGT1A1		CYP7A1
MIP2A	ST13	ALAS2		ELAVL1
MIP3	SIRT6	ERAL1		PTPN22
MIP3B	LRP1	MTR		SHBG
MIP5	SLC22A12	MYL3		CCR6
MK1	ADI1	SOCS3		HSP90AB1
MLC2	G6PD	P2RX7		SLC33A1
MLN19	SETD7	AIRE		EZH2
MMIF	S100A8	IL15		NTRK2

MMP14	NES	PLAUR		KDM5D
MMP2	TWSG1	GATA4		NOS2
MMP7	GHSR	MTTP		CD80
MMP9	SERPINC1	CR1		AQP1
MPB1	ACVR2B	IL1RN		FCGR2B
MPIF1	SPARC	ITGB1BP2		CCL15
MPIF2	PON2	RBP1		GOT2
MPO	ANGPT2	PLA2G7		GSK3A
MPSL1	PSEN1	VCAN		PLPP3
MRLC1	TNS1	CREG1		KDR
MRP6	SIRT2	FST		SERPIND1
MSK12	FOS	GZMB		CSF1
MSK8	CLEC6A	STS		VAMP8
MTBT1	TGM2	SCG2		APLN
MXR	C1QTNF1	DDAH1		FASLG
MYL9	ELOVL6	ADI1		NAXE
MYRL2	STOML2	IL23R		TERT
NACP	MANF	EPHB2		IL9
NAT1	FHL2	TET1		CORIN
NAT3	TRPV4	ENPP1		BMP4
NAV2	SP4	KL		LTB4R
NBC	ABCC6	FTO		HPSE
NBC1	LRP6	LIF		APLNR
NBCE1	RALA	BID		RAPGEF5
NCAM	SULT1A3	DENR		TNFSF4
NCAM1	CPSF4	IGF2		LOX
NCC1	ADM	CAV1		CTSS
NCC3	MRRF	CXCL10		GPBAR1
NCSTN	CDKN2A	CTSB		MS4A1
NDF	DKK1	F5		CYP2C9
NEGF1	SULT2A1	NDNF		MMP13
NEGF2	STS	NR0B1		CAV1
NEU	MAEA	FCGR2A		NOX1
NGL	SLC25A3	FAIM2		MAPK9
NKSF2	S1PR1	P2RY2		PPIA
NLT	STH	KNG1		ALDH2
NMB	FZD2	HDAC5		MAFB
NOG	PIK3CD	CEACAM5		STAT4
NPPS	CRH	KCNE1		SLC17A5
NRG1	TBCD	NFKB1		GJA4
NRP	IL18BP	TP53		OGN
NRP1	GSC	ARG1		S100A12
NRTN	EGLN3	PTPN1		ARG2
NT5	RNF146	SERPINA4		MTHFD2
NT5E	CNR1	FKBP1B		ITLN1
NTCP	CCL7	BBOX1		GPI
NTE	AIF1	BRCA1		KLK1

NTN1	CCL19	PCBD1		PRL
NTN1L	RRM1	LRG1		FLT1
NTT	KLF15	CDH2		KLF2
OAT2	IHH	CD69		AEBP1
OCIF	NOTCH1	LOX		ACKR3
OCLN	MCF2L	NRG1		SLC9A1
ODZ1	SMAD7	F11		CYBA
OP1	FGFR2	VAMP8		BMP7
OPG	TIMP2	FGF9		PNPLA2
ORAI1	PCYT1A	CYP2C8		IDO1
ORCTL2	CCL18	ERBB4		GLP1R
OSF2	HBD	NPPC		TAGLN
OTASE	ULK1	UCN		SELL
OZF	AGXT	UMOD		S1PR2
P4HB	PRKCB	CCL21		XPR1
P5	RUNX1	LYST		P4HB
PACE	RTCA	LPAR3		ACSL1
PACE4	RAF1	IL13		CD68
PAFAH1B1	AOC3	DYRK1A		ADK
PAFAHA	INHBA	CSF1		SERPINA5
PAI1	CD274	JUN		ADAMTS7
PAIRBP1	CRBN	BTN2A1		PTK2
PALB	RHEB	STC1		LEPR
PAN1B	PTN	ANGPT2		ESAM
PARK1	TJP1	CNR1		PLAT
PBP	MLYCD	ACR		XBP1
PC1	HGS	LTA4H		LPL
PC5	DMD	NPY4R		TIMP2
PC6	WT1	ENO3		PGF
PCFT	BDKRB2	CDKN2A		CDH13
PCI	STC1	NNT		SORL1
PCOLCE2	NCAM1	TGFB3		CDK9
PCPE2	HRC	RECQL5		TXN
PCSK3	SH3BP4	ADAMTS4		SYK
PCSK5	BMP4	EFNA1		APOA2
PCSK6	MC3R	HBD		ROCK1
PDCD5	MPG	CIITA		P2RY13
PDGF1	MCAT	ABCG2		PLIN1
PDGF2	TP53INP2	FABP4		KLRK1
PDGFA	ACADVL	E2F1		CYP2J2
PDGFB	NTN1	NOX5		OSM
PDI	VIM	IGFBP3		TXNL1
PDIA1	RNH1	EDA		PECAM1
PDIA6	CD47	GPR17		COMT
PDNP1	ADA	MCIDAS		SGMS2
PDNP3	EPHB4	PLK3		SOAT1
PEBP	SPHK1	LAIR1		LBP

PEBP1	COMMD1	STIM1		LILRB4
PECAM1	ADORA2B	HMGB2		LGMN
PEDF	MVD	NRDC		THBD
PF4	BCL2L11	PON2		DDX58
PF4V1	TMEFF2	AKT1		SMAD7
PFC	TPT1	DCTD		ADM2
PFD	GFAP	HCN4		CRH
PGF	ITPR2	SORBS3		CD248
PGFL	TF	C3		POSTN
PGY1	NOTCH3	MMP28		NOS1
PGY3	ELANE	IRAK3		CXCR2
PI	CDH5	AMY1C		KCNN4
PI7	SMAD2	TFAM		PCSK7
PIG35	ITGAM	HSD11B1		MMRN1
PIGR	STIM1	ZNF627		ICOS
PIT2	ASGR2	HSPA12B		TNNT2
PLA2B	SST	CD180		APOA5
PLA2G2A	PTH	PEAR1		LDB2
PLA2G5	AOPEP	POU2F3		IL22
PLA2L	KRAS	DPYSL2		APOL1
PLANH1	STIL	MYDGF		F3
PLANH3	PLA2G4A	CD163		GSTO1
PLAT	MMD	CYP3A4		CHUK
PLAU	TNNT1	SGCA		CCL11
PLBD1	HMOX2	SERPING1		ST6GAL1
PLG	BAMBI	SERPINA6		TNFAIP8L2
PLGF	PTHLH	MTAP		FUT7
PMCA1	EIF2AK1	MAPK7		HCAR2
PN1	RAPGEF3	APOD		MYOCD
PO4DB	NTHL1	SERPINH1		FCGR2A
POMFIL2	FOXP3	EGFR		CTSK
PON	ANK1	TTL		NOX4
PON1	GH1	GAPDH		IFNB1
PON2	CHUK	VTN		CD27
PON3	MT-ND2	TBXA2R		NAT2
POSTN	ANXA6	JAK2		EPHB2
PP4	NPY2R	SYT1		TF
PPBP	CADM1	CYBB		RPS3A
PPIB	PLD6	SH2B1		CD38
PRDX4	BCL2	CLEC3B		ATG5
PRELP	FPR2	SPHK1		HRH1
PRG2	GLB1	CXCL9		LIPA
PRIP	GUSB	CNP		CD6
PRL	PTK2	SETD2		MVP
PRNP	PRKAA2	IL9		PTAFR
PRO0309	AIFM1	IMMT		FURIN
PRO0684	AK3	PPY		IGF1R

PRO1400	TP53	HSPA5		JUP
PRO2061	ASGR1	F11R		TWIST1
PRO2209	ESD	TNFSF18		TRPV4
PRO2290	TCHP	ADRA2B		AHSP
PROC	CD36	FSTL1		TRAF1
PROCI	UBQLN1	FGB		ADAMTS1
PRP	CYP2J2	KLK3		CCL17
PRSS1	FGL2	TNFRSF10B		CLEC5A
PS1	TCF21	FLNA		ADAM10
PSEC0029	ETV3	GSTCD		CXCL8
PSEC0101	PLEK	XRCC3		GSK3B
PSEC0139	TACR1	KBTBD7		S100A8
PSEN1	CCL20	ADIPOR2		EMP1
PSNL1	IL4	CDKN1A		IGFBP3
PTCH	OPRM1	MAEA		TICAM1
PTCH1	LPCAT3	MMRN1		JAK2
PTN	CREB3	NOX4		CD28
PTPRC	RARRES2	CD28		PCYT1B
PTX2	CASP2	GCLM		MEFV
PUMP1	MET	BECN1		IL19
PWD	UBR5	SDC2		PRH1
PWTSR	CABIN1	FADS2		GZMB
RAINB1	CD59	SGK1		LGALS1
RAP3	APOB	HPS1		SMPD1
RASF-A	SYT1	APOA4		CDKN2B
RNASE5	CALCRL	RRS1		CLSTN2
ROBO1	F7	MARCKS		MMP14
RPL22	EIF2AK2	P3H3		CD180
RPL29	ELN	PTK2		ITGA5
RPSA	GRN	SULT2A1		PRDX4
RSPO1	TBXA2R	UCN2		RPL17
RSPO2	CD38	TNNT1		SRY
RSPO3	AK2	GCLC		CCL4
RSPO4	VCP	EPHX1		CPB2
SAA1	NUCB2	MAP2K1		RGCC
SAA2	GCH1	TCP1		CASP3
SARP2	IL37	CCL5		GH1
SCDR9	CXCL17	PRDX2		CD86
SCN5A	DRD2	THPO		IGFBP1
SCYA1	APP	ACKR3		ENPEP
SCYA10	CXCL16	FBLN2		ARSA
SCYA11	MYC	WIF1		TNFRSF25
SCYA13	PGAM2	DKK3		SPARC
SCYA15	CBFA2T2	AZIN2		ECE1
SCYA17	SRC	NTS		STEAP4
SCYA19	SAT1	NOC2L		FTO
SCYA2	LTB4R	AK1		ANGPTL3

SCYA21	NR3C2	SLCO1B1		BSCL2
SCYA22	MYOCD	ST3GAL4		MAP3K5
SCYA23	TTR	TXNL4B		MAEA
SCYA24	PGRMC1	MSTN		GHSR
SCYA25	ADORA1	TCFL5		INSR
SCYA27	ANGPTL3	SERPIND1		LPCAT3
SCYA28	XBP1	CCL4		CAT
SCYA3	SYNE1	SHBG		SREBF2
SCYA4	TLX1NB	LGALS9		PSRC1
SCYA5	CLCN2	OTOR		PXDN
SCYA6	TRPC1	XIAP		CADM1
SCYA7	SHH	SCAI		PSD
SCYA8	HSPG2	PDE4D		MYRF
SCYB1	HSF1	SUMO1		MTTP
SCYB10	TNS3	ELAVL1		AHRR
SCYB11	IFIT2	IL1A		MAPK3
SCYB13	SHD	MATN1		EDNRB
SCYB16	TPR	SORT1		S100A7
SCYB2	CFI	ACP5		IRS1
SCYB4	FGFR4	SLC22A4		IRF2BP2
SCYB4V1	TRPM4	RAMP1		CCL19
SCYB6	HSPB8	SARS		C1QL3
SCYB7	CMKLR1	IL32		NCEH1
SCYB9B	MFN1	SRF		CP
SCYC1	ILK	CCL16		GSS
SCYD1	S100B	ABCD1		PGLYRP1
SCYE1	GSN	ITGB1		F2
SDF1	ARID3A	PHGDH		ADAM33
SDF1A	BCHE	ARC		CCR7
SDF1B	MAPK14	DEFA1		MMP3
SELL	TFAM	BGLAP		SH2B3
SELP	LDHB	TLR9		ZNF202
SEMA5A	CD4	IL17D		CTSL
SEMA5B	PDC	FBXO32		LRP2
SEMAF	PICALM	CCL3		IL23R
SEMAG		BMP2		CD70
SERBP1		TLR7		DDR1
SERPINA1		OSTN		TBXAS1
SERPINA10		SST		NOD1
SERPINA3		TRPC4		GNMT
SERPINA5		GC		GATA4
SERPINA6		CAV3		SLC25A1
SERPINA8		CCL20		CES1
SERPINC1		SLC25A3		IL17RA
SERPIND1		DCD		MGLL
SERPINE1		ILF3		SERPINC1
SERPINE2		DCC		TCF7L2

SERPINF1		SLC25A10		ANXA5
SERPING1		LPO		CD99
SFRP1		IRS1		MIF
SHH		KCNMA1		CYP27A1
SIAT1		MAP3K3		RELN
SIS		STAT1		PSMA7
SLC10A1		FOXP3		CAPN10
SLC12A9		BMP10		HBEGF
SLC16A1		HUNK		TLR6
SLC16A2		ARRB1		CXCR3
SLC20A2		GOSR2		FGF1
SLC22A1		CSF1R		CYP19A1
SLC22A18		CCR1		PRLR
SLC22A1L		MAPKAPK2		CDH5
SLC22A7		SRI		CXCL1
SLC2A2		TNFAIP6		CISH
SLC30A1		CASP8		IGHD
SLC38A3		LCAT		ITCH
SLC38A4		NFATC3		B2M
SLC39A4		FLT3		ACR
SLC3A2		CD2		ADAMTS13
SLC46A1		ADRA2A		NFE2L2
SLC4A1		FETUB		TFAP2A
SLC4A4		FXYD1		GET4
SLC7A2		HABP2		CXCL10
SLCO1A2		SPRR3		APOH
SLIL1		KRT75		LRP5
SLIL3		CIART		TEK
SLIT1		CELF1		F11
SLIT2		TNNI3K		ABCA2
SLPI		PIK3C2A		SCAI
SLRR1A		GHRL		ZG16B
SLRR2A		HSF1		NCF1
SMDF		CHP1		USF1
SN1		PRKD1		SERPINA1
SNAT3		XPR1		TNFRSF13B
SNAT4		SELL		ECSCR
SNCA		KRT76		NEXN
SOD1		MAF		JAML
SOD3		ADAM15		HSD11B2
SORT1		GRAP2		TNFSF8
SOST		CXCR3		NR0B2
SRPSOX		IGF1R		LSS
ST6GAL1		VLDLR		GPBP1
STAB2		APOM		CYP26B1
STAMP2		FAP		STK11
STEAP4		KLRK1		NR1H3

STEERIN2		SLC22A5		NR2F2
STG		SMTN		RGS1
STIM1		BRD4		CYP2C8
SYNGR1		SLPI		CCL21
TALL2		KDM1A		CIC
TARC		CTSK		LGALS3BP
TAU		BTG3		JAZF1
TECK		CYP2D6		IRF3
TF		CD68		CCL24
TFAR19		ILK		PLA2G15
TFPI		CYP4A11		CRY1
TFPI1		CSAD		MAPKAPK2
TFR2		ACVR1B		IL15
TFRC		ENTPD1		GP6
TG		PSD4		P2RY2
TGB1		HRH3		AKT1
TGFB		KCNQ1		NPC1
TGFB1		GP5		PREX1
TGFB2		CD27		RIPK3
TGFBR3		RNLS		TGFB1I1
TGM2		MTHFD1		ANGPTL6
THBGB1		HSP90AB1		BECN1
THBS1		ABCG1		SEC14L2
THBS2		TTN		SSB
THBS3		ACHE		HDAC3
THBS4		MAP3K5		F2RL1
THSD2		TRIM63		HSPA1A
TIMP3		ANXA13		RNF5
TIP		ADH1C		SCUBE2
TKF		WT1		CREB3L3
TM14		SMAD2		PNPLA3
TMEM142A		CPOX		GYPC
TNC		DRD1		HAVCR1
TNF		ALPI		AHR
TNFA		PEPD		CD81
TNFAIP4		AMPD1		TNFRSF8
TNFAIP6		CCL22		PRDX6
TNFAIP9		HCN2		CYP1A1
TNFRSF11B		ADAMTS2		ROCK2
TNFSF13		ANPEP		GUSB
TNFSF2		PDCD4		EEF2K
TNM1		NOD2		SERPINF2
TNX		BCL2L11		C5AR1
TNXB		PXDN		TTC39B
TNXB1		NUCB2		MGP
TNXB2		RYR2		GPX4
TPS1		GCGR		CBS

TPS2		CD63		PTHLH
TPS2		PDCD1		AOPEP
TPSAB1		CCR4		PKM
TPSB1		CXCR2		BCHE
TPSB2		AXL		EPHB1
TRDX		SLC6A18		FLNA
TRP1		ABCG5		FUT4
TRX		KCNN4		GPR55
TRX1		TYMP		VEGFD
TRY1		APOA2		MAZ
TRYP1		ATF6		TFPI2
TSG6		SSB		HSPA5
TSP		EGLN1		PRDX2
TSP1		PCSK5		STS
TSP2		AVP		CEBPB
TSP3		ANGPTL3		TAP1
TSP4		AGER		TFF3
TSSC5		SORBS2		LTA4H
TTR		QDPR		COPS5
TXN		INSIG2		GRK5
TXNDC7		GLI1		NOS1AP
UND		PKM		EPO
UNQ161/PRO187		MET		HFE
UNQ1725/PRO9925		NPAT		AR
UNQ1840/PRO3566		NTRK2		LPAR4
UNQ1874/PRO4317		IL19		CYP2C19
UNQ207/PRO233		FPR1		ADD1
UNQ217/PRO243		SLC8A1		SGCA
UNQ250/PRO287		CHRNA5		TNFRSF18
UNQ2500/PRO5800		CDA		TSPYL2
UNQ2563/PRO6243		DGKZ		ERN2
UNQ2748/PRO6487		PRDX5		GPLD1
UNQ2759/PRO6714		CTSA		SIGLEC1
UNQ2914/PRO1156/PRO9783		C5orf38		CTF1
UNQ2976/PRO7455/PRO7476		ICOS		PLG
UNQ383/PRO715		RUNX1		CDH1
UNQ387/PRO719		KLF2		MTRR
UNQ411/PRO773		PTHLH		GCH1
UNQ420/PRO856		NTRK1		PRDX1
UNQ497/PRO1014		NAAA		HP
UNQ5867/PRO34001		GJC1		DES
UNQ611/PRO1198		MAMLD1		LMNA
UNQ707/PRO1358		MAP2K3		CCL7
UNQ784/PRO1600		LTB		TNFAIP6
UNQ9384/PRO34209		SEMA3A		HRH2
URB		ADIPOR1		ATM
VEGF		PCTP		ABCB1

VEGF165R		TOLLIP		ADAM15
VEGFA		AMELX		COL15A1
VEGFB		SERPINB2		F8
VGR		PYCARD		PCNA
VNRA		IRAK1		BMP2
VRF		HAVCR1		USP20
VTN		MASP2		SULT1E1
VWF		UCP2		SASH1
WAP4		ECM1		RPS6KB1
WC1		CRYAB		ALOX5AP
WFDC4		TGFBR1		NEU1
WND		GSK3A		AOC2
WNT1		HPSE		MDK
XB		SMPD2		APOF
XCL1		AREG		DNASE1
XDH		MRPS6		MAP4K5
XDHA		SLC5A3		TFAM
XPCT		IL12B		CFTR
ZIP4		THBS2		ANG
ZNF146		PRKN		LPIN1
ZNT1		COL1A1		CEL
ZPI		TBXAS1		PPL
ZTNF2		SREBF2		TLR3
		ANXA3		BMPR2
		PZP		IL1F10
		SRY		ENPP1
		LCT		PALLD
		ROCK1		PCYT1A
		PDX1		ITGA2B
		ANK2		STAB1
		P2RY11		GNB3
		GFAP		ANGPT2
		HRC		SLC25A22
		SNX17		ST14
		MCF2L		TGFB3
		IL22		RNLS
		RHOJ		CR1
		TYR		SELENOS
		PROKR1		FST
		S1PR2		CD276
		IRAK4		ADA
		CYP2R1		RSAD2
		BMP1		IFIH1
		TSC22D3		TRAF5
		RASA1		SPDEF
		SELPLG		CXCL13
		NR1H4		ITGB3

		CXCR1		KITLG
		TM6SF2		CARD16
		SMAD4		CERS5
		DST		OGA
		CSRP3		EPHX2
		GPR65		SERPINA10
		LAMA3		COL14A1
		KLF10		LIAS
		IL4I1		CLEC7A
		SLC25A4		AGT
		ATG7		LIF
		FLOT2		SOD3
		GPX1		TRPC1
		GJB1		UCP2
		TES		CCS
		APCS		LTB
		NMI		MSR1
		MAP4K5		SFRP5
		KCNJ11		CYBB
		CLEC6A		G6PD
		NELFCD		ETV3
		BMP7		CXCL5
		GPKOW		AIM2
		CD274		FFAR4
		SKAP2		CHST11
		PDLIM7		TPP2
		SIRT2		TMEM98
		S100P		VEGFB
		SIRT5		HPGDS
		CPNE3		VTN
		TAPBP		ADRB3
		UGCG		MLKL
		APOL1		GCK
		CDH1		TTR
		RBP4		LSR
		P2RY4		MTX1
		PPIP5K1		RBM45
		CHD1L		DHCR7
		CALD1		UTS2R
		PTGR1		NEXN-AS1
		TRAF3IP2		MMP17
		STAB1		SERPINA9
		SCN4A		NQO1
		DSE		MYLIP
		TYRO3		SMPD2
		DDIT3		RNF213
		GNLY		NLRP1

		TBPL1		ITGB2
		TBX1		TYMP
		SOX7		GLA
		CYP7A1		RORA
		RCAN1		FHL2
		TNS3		AKR1B1
		CD70		FGF19
		DVL1		SQSTM1
		SGSM3		HACD4
		ITCH		AMOT
		PTK2B		ATF2
		HK1		CNN2
		STK11		LTBR
		CIC		IL3
		DPYS		JAK1
		TLX1NB		PCSK5
		CCL18		SETD2
		GPX3		PIK3CD
		CPT1B		PEBP1
		RGS7		THBS2
		P2RY6		PRMT3
		KRT74		LGALS9
		SMPD1		LMOD1
		CPT2		IVD
		JPH2		CR2
		LIMD1		KDM4A
		NIBAN2		CCL20
		SAA2		A2M
		DPP6		CCL27
		RHBDF2		MAOA
		SLC25A16		LPP
		CORO1A		CYP11B2
		SIRT6		LTF
		GIPR		RIPK2
		FCN2		CCR1
		DDAH2		PVR
		AGPAT1		TNFRSF1B
		ARSI		ANXA1
		LCK		CHGA
		ITIH3		ARNTL
		UCHL1		PI3
		HMGA1		SPN
		CDKN1C		SENP2
		PKN1		PRKG1
		ALMS1		DHRS2
		UNC5B		JUN
		PRORP		MME

		HDAC9		GP1BA
		NCF1		C3
		SOD1		GIP
		ACTA2		INPP5K
		FCAR		GRAP2
		TXNRD2		GC
		TRAF6		POU2F1
		RBPJ		TSPO
		CXCL1		CCR3
		LRP5		AGXT
		CANX		TAS1R3
		JAG1		HTR2A
		RNH1		ADAM9
		CYP3A5		OSBPL8
		MAP9		CEBPA
		ARID3A		TTPA
		DNAJC5		FDFT1
		EGF		LPO
		CD93		YBX1
		CORT		LPXN
		ATP6AP2		HSF1
		EMC10		MNDA
		PCSK2		RGS5
		IL17F		PODXL
		HNRNPUL1		ORMDL3
		SF3A2		KLF5
		DOT1L		MALT1
		CLEC16A		CCL3
		SEMA3F		PDE4D
		KAT8		HDAC1
		PNOC		THBS1
		ACTB		TCFL5
		MVP		CWC22
		SELENOP		QKI
		DDRGK1		CPB1
		PELI1		PPP1R3B
		NPY2R		CREG1
		GREM2		ABCD2
		CITED4		EGF
		CEBPB		CAST
		AR		GDF2
		COL3A1		RHBDF2
		PTGDR		HDLBP
		DIS3L		TBXA2R
		PRDX3		PTGDS
		GYG1		CEACAM5
		PDPK1		APEX1

		MAP2K5		SIRPA
		KMT2A		HRAS
		NFE2L2		PSMB8
		KRT18		ADCY9
		LGALS3BP		ABCA5
		OXT		CARHSP1
		NRP1		PTGES
		ST6GALNAC1		MMP11
		SULT4A1		ERCC8
		PAEP		MYD88
		TSN		IRF8
		ADCY10		RTN3
		SMS		MT2A
		PLK4		CCN2
		FEV		FASN
		FOXG1		S1PR3
		CASP9		INVS
		SMC3		TCF21
		CFB		HPX
		ITGA1		HNF1A
		ACTG1		PCOLCE2
		GPR132		NPR2
		MYEOV		SEMA7A
		MTNR1A		FA2H
		KLK6		NINJ2
		TKT		CNP
		TTPA		GATA2
		TPH1		GCKR
		CFP		TET1
		IL10RA		ALOX15B
		DKK1		TXNL4B
		CUL7		FOS
		CACNA2D2		FABP2
		CAMKK1		SLC16A8
		PARK7		TRPC6
		FLT4		SDC1
		S100A9		CD209
		PTGIR		ARG1
		BNIP3		CTH
		EMP1		PTPN11
		NANOS1		NCOR1
		P2RY13		TGM2
		MRS2		CCL28
		SFRP4		HTRA2
		STXBP2		C1QTNF5
		ROS1		IL34
		CCAR1		MFN2

		NEDD4L		BCL2A1
		APLNR		ADAM12
		TPO		CD274
		PTS		MSTN
		EGLN3		ANGPT1
		P4HB		CYCS
		ATF7IP		CTSB
		LPAL2		STIM1
		TBC1D10C		NME1
		KRT1		PTPN2
		SLC22A3		SAMD1
		RPL17		SLPI
		CFI		MBTPS2
		SERHL		SIRT3
		DSG3		APOC2
		AKIP1		NOX5
		FABP1		CTSD
		TAS2R50		NAP1L4
		RNASE1		ACTG1
		SH3BP4		NR1I3
		NFKBIA		PYY
		TERT		ID3
		HGS		PLCB3
		S1PR1		SRD5A1
		PLTP		SRGN
		HNF1A		FOXO3
		FZD1		SMS
		NR4A2		GALNT3
		HM13		ILK
		SLC5A1		PPP2R2A
		REG1A		F12
		IFNB1		MCF2L
		TBP		EFNB1
		CBS		LRG1
		JDP2		GUCY1A1
		TLX2		MUC16
		GAD1		KISS1R
		GLA		MB
		CDH5		CLIC1
		F13A1		CDKN1C
		MPG		NR5A2
		COG2		SRF
		PML		ERCC1
		GPR35		MAPK1
		NRF1		MMP7
		GSDMD		SERPINE2
		CD44		CARD10

		NF2		PHGDH
		CCK		FBLN1
		CD19		IRAK1
		AHSP		RBPJ
		CLEC7A		RP9
		SFTPB		EPHA1
		G6PD		CD63
		BDKRB1		DOCK4
		VIP		IGFBP2
		AMH		TYROBP
		SPESP1		NEFL
		FAM111B		NPPC
		ACOX1		CCAR1
		AIFM1		CFD
		DBT		DDIT3
		SAT1		CYGB
		PDC		FADS1
		RASA4		CHIT1
		CA1		NECTIN1
		DCTN3		RHOA
		MTRR		RALBP1
		GAST		SPRTN
		ERFE		PDLIM7
		C1QTNF3		ADM
		XRCC1		CCL23
		CCL19		POR
		AOC2		SERPINA4
		OGG1		TP53
		CREB3L1		TNFSF10
		CARD8		CTSG
		GDF11		DDAH1
		CDH13		PEMT
		LMNA		NEIL3
		FNDC5		CCDC80
		SORD		IRF1
		IGF2R		TNNI3
		MANF		IL1RL1
		CFD		ACKR1
		AGRN		GDNF
		NPR3		GHRL
		ACD		CAMP
		MTX1		RB1
		FCGR3A		FBXO3
		TLR1		IL21R
		FCGR3B		STAT6
		FCGR1A		IL7
		TCF7L2		S100A6

		KLRG1		ENPP2
		TRPC3		CYLD
		FCGR2B		FLCN
		PTPRU		TWSG1
		SH2B3		MT-CYB
		TRIT1		MDM2
		IL5		AAMP
		TCN1		SPHK1
		ACADM		FADS2
		ETV3		NAAA
		TPI1		NFATC1
		AGXT		MIA3
		NAP1L4		C1QTNF3
		CARD17		NPR3
		DIPK2A		PNLIP
		C1QTNF1		IL21
		IGFBP7		ENTPD1
		FADS1		IL1R1
		EFNB2		AVP
		ECD		MICA
		LYVE1		CSTB
		AVPR1A		PLN
		VIM		SYNPO2
		ECE1		FABP1
		ENO2		PROX1
		PSMA7		SUMO1
		FAIM		MRC1
		AGXT2		THBS4
		SS18L1		AOC1
		BDKRB2		DEFA1
		IRF5		SLCO6A1
		MAP6		CALR
		ACPP		SELENOP
		SLCO6A1		FADD
		MT-ND2		SERPING1
		HIGD1A		IRAK3
		HSPB7		RHD
		IHH		FOSL1
		TRIM54		SCARF1
		UBD		IARS
		FGA		SPRY1
		ASGR1		RTN4
		HTR1B		CARD8
		CASR		SLC12A3
		CLSTN2		VIP
		PTN		DCD
		DMRTA1		CRLF2

		VEGFD		GAS6
		TBC1D4		CLEC4C
		ARNTL		ARRB2
		CA6		INTS6
		CUEDC2		NGF
		TNRC6B		BCR
		FGFR1		ABL1
		LRP6		NCOA6
		C4BPA		AQP7
		CD55		ADGRE5
		ADCY3		BCL3
		URI1		NPR1
		MYL2		SERPINB2
		CCN4		APOBR
		TIGAR		KALRN
		EPRS		TLX2
		C4A		DOCK7
		NKX2-5		ZGPAT
		ANGPTL4		ROR2
		MAT2B		BDKRB2
		VEGFC		TNFSF13
		PGAM5		ASCL1
		CCL28		CPE
		PLN		SALL1
		CARD9		FTL
		TNFRSF4		MYLK
		PCSK7		PDE3B
		PDIA6		CD9
		SLC11A1		FMOD
		RIPK2		SLC2A1
		KRT3		PARK7
		ALPK1		PANX1
		GDF10		TARDBP
		EDN3		FCN2
		WLS		ICOSLG
		JUP		TRIP12
		PPARGC1B		ADHFE1
		HDGF		UNK
		PALLD		ADGRE2
		NDP		DSTN
		PTPN22		TMSB10
		SERPINA7		CFH
		DUSP4		TXNIP
		EMD		PCBD1
		ABCC8		SGMS1
		FGF16		UCN3
		SREBF1		PARP14

		BIN1		LEF1
		NPY6R		INO80D
		PPP1R10		NNT
		OR13G1		LILRB1
		IVL		SAMHD1
		BTG2		HS3ST1
		TMEM123		ADTRP
		PHLDA1		RAG2
		DNMT3A		IL1RN
		MEFV		EIF2S1
		GATA3		NUCB2
		MAG		DDR2
		ALG1		WRN
		CASP12		APH1B
		MT-CYB		MERTK
		IFIT1		MTRNR2L8
		SSTR2		CIDEC
		JMJD1C		NBL1
		FGG		SLC5A10
		DDR2		ABCA12
		OGT		MASP1
		CTSL		MASP2
		PRDM2		HSPB3
		CRX		NNMT
		TRIB3		IRAK4
		ELOVL5		SH3BP4
		PGLYRP1		RBPMS
		SERPINA3		PLXNB2
		STH		CPSF4
		SMAD1		SOX6
		EGR1		VAMP3
		PRSS27		CUL3
		DRAM1		IL1R2
		LPXN		CTNNB1
		CTSG		GRK2
		PLD1		CLEC6A
		GRK5		FCGR3A
		COL1A2		SYNM
		PLA2R1		CCR9
		TLN1		CSGALNACT2
		DAO		DFFB
		GYS1		TPM4
		PIAS1		AIRE
		PER2		ADIPOR1
		SERPINB6		CIART
		SFRP1		ITGAD
		P2RY1		SORD

		NCAM1		DSC1
		LDHB		ZFP36
		APOC2		IDE
		LDHA		SCP2
		SH3BP5		DSE
		APOBEC3G		P3H3
		IRF9		PITRM1
		IGHM		METRN
		MT-CO1		AHCY
		C5AR1		SH2D3C
		ZGPAT		ACHE
		KRT77		CHI3L2
		DPPA3		RUNX3
		PMCH		ADIPOR2
		RBM10		CPN1
		IFNL1		TLR1
		SYNM		NR1D2
		NR1D2		ACVRL1
		GLRX		DDAH2
		NPTX1		UCP1
		ALK		CASR
		MRAP		MUC5AC
		AQP3		GP5
		ATP7A		PATE1
		CDS1		GSTA4
		SQSTM1		SERPINA6
		DFFA		PMPCA
		GJD2		RETNLB
		UBE2Z		NIFK
		DDX4		ARHGAP18
		CGN		ADCYAP1
		ESD		NEUROD4
		ETV6		ZMYM2
		OPN1SW		LYPLA1
		AVPR2		WIPF1
		MYOG		VIPR1
		IVNS1ABP		SELENOK
		GHRHR		NBN
		COPE		EPN1
		NBN		IDS
		CBY1		NR0B1
		CPA1		CD74
		CD5L		PDE1C
		MXD1		SFRP4
		TKTL1		YME1L1
		ERBB2		TGFBR2
		PRKCB		GAD1

		ITPRID2		MVD
		CCND2		ABCG4
		FBN1		NELFCD
		IL1RL1		COL4A1
		CCL7		C5AR2
		GLRA2		ALOX12
		GCFC2		HLA-DMB
		CYP1A1		EIF2AK1
		NF1		QPCTL
		BNIP3L		MLXIPL
		CD38		PROCR
		SLURP1		CA1
		BNC1		NR6A1
		GPNMB		PRKCA
		EPHX2		LYVE1
		DGUOK		COLEC12
		PSD		HDAC6
		NPR2		LUM
		MFGE8		BIRC2
		INPP5K		CLCN3
		MAPK8		ANK1
		ABCC6		NANOS1
		CHGB		PCTP
		LTF		CASP9
		DBI		MIA
		BAX		DLAT
		TNFRSF1B		RARA
		TRIM72		ACP1
		CAVIN4		UGCG
		ITGA5		PSME3
		SMC2		PDGFRA
		SFTPD		CENPJ
		VNN3		MATN1
		AKR1B1		CCN4
		TXNDC9		RNASE3
		BMP4		EGR1
		RIPK1		DMD
		TMEFF1		SAA2
		HBEGF		PDGFD
		FDFT1		SGK1
		PTPN11		CYP1A2
		CRIP1		LRIT1
		ZBTB12		PIAS1
		NOS1AP		C5orf38
		TRH		SMAD1
		SLN		TBP
		ANK3		CARD9

		DLAT		CDK5
		DOCK11		JAG1
		MDM2		HYOU1
		GDF1		RTCA
		EDN2		MAPK6
		MYRF		PITX2
		CACYBP		SMTN
		PDGFRB		SPRR3
		IKBKB		ABR
		RASL12		REG1A
		SENP1		TNFRSF6B
		RCN1		SOX18
		GOPC		BTC
		GRN		CCAR2
		E2F2		EGLN3
		PTCRA		GATA1
		MLKL		GTPBP1
		RRM1		GDF10
		ARIH1		NR4A3
		AGL		PDC
		PIGF		MTAP
		HYOU1		ANXA2
		TNFSF10		TYRO3
		CD80		CBFA2T2
		UTP25		SEPHS1
		SKP1		TP53INP2
		ST8SIA4		HOXA11
		SLC29A1		MMD
		TNFSF13B		HPS1
		SEC14L2		NF1
		BLVRB		CCL8
		TNS1		KCNE2
		UBE2K		MT-RNR2
		ICAM3		CKLF
		EVC		HHIPL1
		LMCD1		SRR
		BCLAF1		BTG3
		DPEP1		STRIP2
		CFLAR		LAG3
		IFNGR2		CDC42
		OPN1MW		ACADS
		ERAS		CD5L
		GAS6		KLF10
		MMP10		TGFBI
		NGB		CEBPZ
		CRH		TRPC5
		SLC5A6		IFI44

		ADAM10		NR2C2
		DTNA		JCAD
		RET		INPPL1
		TFG		STC1
		CNPY2		TNFRSF14
		NEU1		FDX1
		GRK6		BLVRB
		EPHB4		PTPRU
		CNTN2		RAPGEF3
		PENK		PPBP
		THAP5		MUC1
		CA5B		PLA2G2A
		MRRF		PEG10
		ARNT		IGFBP7
		CAPN10		PRSS27
		MMP20		NFKBIA
		MYBL2		PAGR1
		SLC4A4		MT-CO1
		DGKA		DDX41
		PGRMC1		CAV3
		MLXIPL		AKTIP
		CYP17A1		NGB
		FGFR2		ATP7A
		RNF182		EBF1
		ADCY5		KLF15
		TNXB		PRMT2
		FADD		OGT
		CXCR5		DBT
		DKK2		PTK2B
		RGS5		SRM
		TBCD		HAL
		NPFF		MEF2C
		ACVRL1		SLC25A3
		GSC		ESM1
		RAF1		TXNRD2
		DNMT1		MAP2K1
		PLD2		ACP5
		PODXL		SLC2A10
		EZH1		CD151
		NOD1		SERPINB9
		SHD		RGS3
		TLE5		TOR1A
		OAZ1		PTN
		HAS3		PDCD5
		TAB1		CXCL9
		HDAC6		MYH11
		DGCR2		MPI

		TBC1D1		ZC3HC1
		VCL		OMP
		CGAS		ACLY
		EXOSC4		GDF11
		MUC5AC		COL4A2
		LRRK2		INHBA
		PRKG1		SERPINA3
		FZD2		SERHL
		HTR1D		GREM1
		GBA		FBLN2
		CCR9		TIMP4
		MFF		SLC39A8
		NDUFS4		NOC2L
		PTH1R		VCL
		ADK		PLA2G3
		OBSCN		NFIA
		RPIA		FGB
		MERTK		DCTN3
		GPR180		CXCL11
		AMIGO2		FOXO1
		SLCO1C1		E2F1
		SKIL		DPP9
		PAPOLG		ARRB1
		CTSC		SLC2A4RG
		STAG2		HDAC5
		PITX1		DNM1
		MATK		AURKA
		AGGF1		SLAMF7
		SLC2A4RG		AGER
		EGLN2		MRPS6
		MIA		ADCY8
		APELA		IFNAR1
		ASIC3		RPA2
		ONECUT3		FGFR1
		NMS		CEBPD
		OPA1		AMD1
		EXOSC10		CSHL1
		LIMS1		FAM168B
		EVPL		CCL18
		ZNF202		IGFBP4
		MCU		SFTPB
		SMPD4		PLXNB1
		ADAMTS8		PAK1
		MASP1		EDN3
		GRK4		BAX
		INHBA		CEACAM6
		RIPOR2		C1QTNF1

		MAP2K6		ADCY10
		EZH2		UBE2K
		AKT2		PLA2G10
		NDRG1		CELSR2
		SLC25A1		NANOS3
		LRP2		FBN1
		LIMS2		DPEP1
		ITIH4		KIF6
		LAP3		C4BPA
		ATP10D		IL6R
		SRC		TRPM6
		S1PR3		MT1F
		CHUK		NXPH4
		KRT10		STAP2
		CHRM2		DEFB103B
		TBCA		EFNA1
		TACR3		PDPN
		TEF		NR4A2
		HK2		GPR84
		SPAG8		NECTIN2
		ATP8A2		RNASE1
		AKAP12		CHKA
		SHC2		SKAP2
		TST		TBCA
		TRAP1		RPE
		SFTPA1		PLD2
		ONECUT2		YY1
		TCF21		MC4R
		MLYCD		ANXA13
		CD209		AXL
		GCKR		C1QTNF12
		AFM		KCNN3
		P4HA2		TNFAIP3
		SCT		ARSI
		IFNGR1		FGG
		TRPM2		PPM1D
		CD86		VPS51
		LTC4S		TMF1
		C6orf15		APAF1
		KRTAP1-1		EPAS1
		PSMG1		HECA
		DDX41		GNA11
		HCAR2		ATP2B1
		TALDO1		SCT
		TPPP		FYN
		INPP5D		SST
		CKB		NTRK1

		CLMN		IL25
		CTLA4		HAS2
		S100A11		IGFBP5
		CXADR		TRIM63
		PTGES		PLA1A
		PDGFC		ATF4
		MEN1		RPIA
		FLNC		UCN2
		LRRFIP1		CDKN2A-DT
		SRGN		OCLN
		LAX1		GYPE
		SLC2A1		PTMA
		TPSG1		KRT14
		EPHA4		FGA
		FHL2		ITGAM
		CDKN1B		UCN
		IL7		APPL1
		LIPE		CNNM2
		NOTCH3		CYP17A1
		CD6		GNA12
		CLK1		WDR12
		KDM5D		APCS
		PAX6		CAAP1
		NR2E3		RGS4
		CCL27		RASD1
		ATP10A		ABCC1
		TRIM39		MADCAM1
		MUC1		CYP26A1
		CCR3		ITGA6
		TSPAN10		NT5C2
		MYOZ2		SMG6
		FOS		LY75
		RNASE2		FLOT2
		BMPER		ABCC6
		FOXH1		DFFA
		MRAS		CALU
		COMMD7		MCAT
		TMEFF2		CSF1R
		XCL1		PDGFC
		VHL		MYC
		NR4A1		MTFMT
		NPPB		CABIN1
		ODC1		COMP
		POMC		COL1A2
		HPX		CNMD
		ALOX12		MEF2A
		CKM		GRN

		NINL		TFRC
		ABL1		PLA2G5
		MLN		GLG1
		FCN1		ADI1
		LXN		APOBEC3G
		HMMR		PLAC1
		PAF1		PTPRN2
		SPON1		MPZL2
		CNTF		PCSK6
		KIR3DL3		HDC
		ORM2		FBLN5
		MYBPHL		G0S2
		LAT		MMP19
		MT-RNR2		MPL
		RACK1		BIRC6
		NCR3		SPIN1
		HSPA1A		RPGRIP1L
		HLA-DMA		DNAJC5
		CES1		CPA1
		GFPT1		PSEN1
		POU3F4		PLXND1
		IARS		IL18BP
		H2AFX		NT5C1A
		NANOS3		ADAMTS18
		1-Mar		STAB2
		NRG3		NDC80
		PRMT3		RNF2
		ANXA2		GCLC
		LRRC3B		PSMG1
		AURKB		GIPR
		OPLAH		ALX1
		FLII		PODN
		NECTIN3		ITGA2
		RAG2		LRP8
		ADCY6		TMEM18
		CD7		TCHP
		CALB2		NUP43
		BCL2		F7
		SGCD		FLT4
		NRG4		APEH
		AC010330.1		SLC11A2
		BMI1		TPO
		SPINT2		MLN
		CRK		CDK2
		MARS		BTNL2
		CHRFAM7A		STH
		AGBL2		BACE1

		TP53INP1		FEV
		CDCA7L		HMGA1
		TBXT		MFAP4
		CAMP		BMPER
		CXCL3		RSU1
		SLAMF6		PLCB2
		CPSF4		PRCP
		PDXK		EGLN1
		PIP		UFM1
		FGF18		GNRHR
		ART3		ACOT7
		CD96		CXCL2
		MCHR2		HSPB1
		FOXO1		NPS
		TAOK2		S100A10
		PAK1		ABCD1
		ZNF185		GPC1
		MMUT		GCLM
		FARS2		CD24
		BTF3		SLC2A4
		LHFPL2		CLCF1
		POU2F1		ATG13
		PGD		PSTPIP2
		IRF8		CHAC1
		PCSK6		PDE1A
		TCHP		IL11
		HNRNPD		RAF1
		STAT4		TNNT1
		DTNB		FPR1
		ANGPTL2		IL24
		MDFIC		ACD
		KLRC1		ACTB
		KLRD1		ACOX1
		ECHDC3		CTSA
		RSAD2		IL17RB
		ROCK2		CCL26
		ADAMDEC1		GJC2
		CKMT2		NANOS2
		AOC3		RAG1
		GFPT2		HNRNPK
		KIF3A		BRINP3
		RGN		SCUBE1
		TIMM8A		AIFM1
		OPRL1		GSN
		PEX6		HMBOX1
		EPX		GPR119
		EPCAM		SRSF1

		PPL		CSN2
		CASC1		PRMT1
		TACR1		MTR
		MAPKAPK3		QSOX1
		C9		FGF12
		LIFR		KAT8
		RASGRF2		PNN
		ARSB		ITIH4
		IK		CDH8
		MED23		ARSB
		FBXL4		CASP8
		ASF1A		OSCAR
		KLRB1		IDH2
		CD5		CIITA
		DNTT		PACS2
		RAPGEF1		RRS1
		MYL7		MTSS1
		CALB1		GLB1
		ZDHHC2		PLA2G2F
		WFDC1		MSN
		PARD6A		MMUT
		CELF4		KLK3
		IL2RB		CBLIF
		FKBP5		SESN2
		TBC1D2		PRDX5
		ITGA6		OPRL1
		AFP		GADD45A
		YBX1		TBX18
		SLAMF7		GHRHR
		NISCH		PYCARD
		TENM1		TLR8
		IL27		SPTLC1
		EPHA2		ITGA8
		TPM1		PRKCH
		ANXA7		IKBKE
		GLUL		INSIG2
		ZMYM2		MT-RNR1
		PRMT5		HDAC2
		REM1		TNFSF15
		CEACAM1		PRNP
		ERBB3		EGLN2
		MAP4		VDAC1
		AQP2		TNFRSF4
		CBFA2T2		LRP1B
		PIPOX		HPS3
		TNFRSF13C		TGM7
		RAPGEF3		HPD

		PHLPP2		PLK4
		ACSL1		DENR
		ACKR2		FGFBP1
		SLC6A3		RNF112
		BBS9		VASP
		HSPA8		SLC25A19
		KRT25		EFNB2
		CD47		ECD
		RHCE		NID1
		DAOA		ZAP70
		KRT28		TRIM14
		HARS		PLIN3
		KRT86		BAMBI
		AMN		ATG16L1
		PPRC1		YBX3
		AMT		ACPP
		HEMGN		BRAP
		KRT85		DISC1
		ALDH1A2		IFNGR2
		E2F3		NEDD9
		CBX1		DUSP10
		BMF		SCG3
		EEF1A2		CALD1
		TBX21		CEP152
		IL2RA		FXYD1
		PIR		SULF2
		MALT1		PGP
		FRZB		ESRRA
		HMCN1		SCARA5
		TFAP2A		C1QTNF6
		ABCA7		GPS2
		MYBPC3		SIGLEC9
		DDC		HMGXB3
		OXCT1		GAPDH
		IFNLR1		ZMPSTE24
		COL18A1		LMCD1
		GDNF		KMT2E
		TGFBI		SCAF1
		XBP1		WNT5A
		PBRM1		CCL25
		RYR3		CD2
		MYD88		SPON1
		CRY2		C4A
		MBNL1		SFTA3
		DIAPH1		MT1DP
		TRPM4		LSAMP
		DLG1		DEFB4B

		NLRP2		SMPDL3A
		RAD51D		KISS1
		CALR		KRT8
		PRKAR2A		ALCAM
		FBLN1		HSP90B1
		BCR		NDRG2
		PRNP		AGGF1
		HCRT		PBRM1
		EIF2S1		NRG1
		RAD50		SAA4
		CPM		KCNMB1
		POFUT1		SETD7
		DNM1L		VAV1
		NRGN		PPM1B
		FMN1		CLIP1
		SLC12A8		TNFSF14
		KRT14		TRERF1
		HSP90B1		IL17C
		FANCC		DHCR24
		CACNA1B		DBN1
		NBEAL1		SMOC2
		EMILIN1		HSPA8
		DSP		GOSR2
		MYH7		CUBN
		KCNH2		NDRG1
		DSG2		HAS3
		TNFRSF9		HM13
		ZFP90		TRPM7
		ASAH2		CAPN6
		GNRH1		NSF
		MANSC1		RASGRP2
		WTAP		TRO
		CEBPZ		EIF2AK2
		CRHR2		STAT2
		PNPLA3		AMH
		CALCR		PDGFB
		DNAH11		CD19
		SRA1		APOBEC1
		CFL1		CD5
		EEF2		UGT1A1
		MC4R		HYLS1
		PITX2		LSM1
		CDCP1		CTSF
		DISC1		PNOC
		ASRGL1		LAIR1
		PLK2		OR2D2
		ZFHX3		CLEC3B

		PIK3C2G		HCRT
		NAB1		S100A1
		CDC73		EIF4E
		TPT1		TMX1
		RAN		SLCO2B1
		SCAF1		LDHA
		NFKBIB		ITGB4
		ANAPC5		AVPR2
		RGS1		STK25
		TMEM38B		CCT4
		AKAP1		EHHADH
		GSR		AGXT2
		PTPRD		DUSP16
		CA4		FOLR1
		ACSL6		HPN
		DAB2IP		OIP5
		CEPT1		CELSR1
		IVD		DPP8
		GRIN2B		NOTCH3
		AXDND1		IKBKG
		HNF1B		FCGR1A
		PDGFD		OPN1SW
		ADGB		NFKB1
		SHF		CCR4
		SKAP1		OSBP
		WNT4		CDC27
		DGKQ		HPR
		SP7		MYH9
		SPN		GPIHBP1
		PRTN3		CCL16
		NEB		KLRC2
		TNFRSF10C		TRPV2
		MTSS1		CCL13
		BRAF		TMEM11
		NOTCH1		COMMD1
		FBLN5		NPTX1
		TRPC6		CUL5
		MYH10		MRAS
		QPCT		PPARGC1B
		PNN		NUS1
		MCAT		NCAM1
		KCNE4		TERF1
		DAP		SCML4
		SDK1		OSGIN1
		CCDC60		MED28
		TFEB		RGS2
		BOK		CAMKK2

		CACNB3		LRP2BP
		ILKAP		TGFBR1
		COL5A1		BMF
		FOXE1		MLYCD
		MEX3B		CTCF
		PBX2P1		RTRAF
		SLCO3A1		LXN
		CDKAL1		MED17
		C4BPB		BCLAF1
		NTNG2		DLL4
		SPATA7		TNFSF18
		KIAA1217		ACTA2
		MLANA		KLKB1
		ATP1B1		TLX1NB
		CMIP		CYP21A2
		PICALM		TRIM39
		FARP2		SUCNR1
		GFOD2		TACR3
		IRF6		CHAF1A
		CDK5RAP1		VCP
		IL26		NAPEPLD
		HLA-DRA		FGFR4
		HLA-DQB1		PAM
		B3GNT2		RFX5
		CDH12		PML
		EPHA1		HNRNPD
		SIK3		DUSP5
		CHRDL1		POU2F3
		PCIF1		CTSV
		TOPBP1		MTHFS
		MST1		TRIM21
		ANTXR2		SLC5A7
		ZNF433		UNC5B
		SLC1A7		PTOV1
		CCDC63		PSMA6
		KCTD2		CDC6
		LARP6		DNM2
		BMP6		AFM
		WDR64		CCN5
		CNTN4		MAMLD1
		PRKAR2B		THSD7A
		IL1RAP		CFLAR
		TUSC1		TNFRSF10A
		HIP1		DHX58
		EDC4		SLC22A4
		BICRA		HIST1H4L
		SPEF2		GCGR

		SLC28A1		CHIC1
		ATXN1		PRTN3
		AKAP6		SLC18A3
		GAK		CHP1
		SP140		ENPP7
				DAPK3
				S100A11
				CPT2
				BCL2L11
				UBC
				ATP10D
				DAB2IP
				CDK4
				EHF
				HILPDA
				DDC
				PTGIS
				AMPD1
				PRRX1
				ABCB6
				BCL6
				GHR
				GMNN
				ENO2
				CDKN1B
				TCIRG1
				LGI1
				BMPR1A
				ACSL4
				PHPT1
				H6PD
				EPHA3
				CALCR
				TNFSF12
				DHFR
				ATP10A
				TMSB4X
				SAMD9
				PLCG2
				ADARB1
				PRELP
				SRSF10
				FPR3
				P2RY4
				INSIG1
				PDIA2
				ANKS1B

				RGS7
				ARL6IP4
				CCR8
				PELI3
				HOXD4
				RNASE2
				CHRFAM7A
				CLEC1B
				RAD21
				SLC2A2
				IFI16
				CLDN1
				SRSF2
				ZFYVE9
				HMGA2
				SLC25A25
				ECE2
				CD53
				GATA6
				ABHD2
				CSAD
				ITGB7
				PPCDC
				LCP1
				TSPAN2
				VHL
				RAC2
				OMD
				CRHR1
				GNA13
				RCN2
				PIK3R3
				PLA2G2D
				ITGA4
				LMNB1
				SLC17A2
				BACH2
				CYP27B1
				HGFAC
				PDLIM1
				ERCC2
				CD99L2
				LPIN2
				BRD1
				MSH2
				XRN2
				ITGB5

				RARB
				IGF2BP2
				NEDD4
				SPI1
				TAB2
				SOD1
				SSTR2
				S100B
				SLC30A8
				SNCA
				APOC4
				GALNT4
				RTL1
				TCTN1
				HSPA1B
				F5
				PDGFA
				ANXA4
				S100A4
				LIPI
				PLA2G2E
				IFNA2
				SECISBP2
				CCK
				MAPT
				RXRA
				SP1
				HGS
				OSBP2
				PROS1
				LIN9
				GABRR3
				SLC25A10
				AP2A2
				TH
				ZBTB7A
				MST1
				RELA
				THEMIS
				KRT27
				CAVIN3
				AKAP13
				SEMA3E
				CDK1
				IL7R
				MARS
				RPL27A

				SGPL1
				BRD7
				IL31RA
				HPGD
				MAP9
				CADPS
				CXCR1
				CDC42EP3
				SSTR5
				FDPS
				SNF8
				UBE2Z
				HTR5A
				DBI
				KCTD15
				HHEX
				SPARCL1
				DCLRE1C
				BAG3
				PTS
				DRD2
				IL2RG
				GPR6
				CDKAL1
				CXCL14
				MYH15
				TMEFF2
				NAT8
				RHOB
				GUK1
				RORC
				RPTOR
				ZFHX3
				CELA1
				KLRG1
				SCARB2
				ATIC
				RDX
				YAP1
				LRRC32
				EDAR
				KLRD1
				YWHAQ
				GSC
				LPIN3
				SSTR4
				COL5A1

				UBAC1
				EDN2
				THRA
				RUNX2
				ATF1
				OPTN
				CHST3
				POLK
				PIK3CA
				IAPP
				SNX19
				PRG4
				NPHS2
				SOS1
				ODC1
				IL17F
				MVK
				CPT1A
				CTSC
				MPP2
				CPD
				SORCS1
				MICU1
				CREB3
				BLVRA
				NRF1
				PHF14
				CAV2
				SIGLEC5
				CD79A
				AKT2
				CD37
				SQLE
				ZDHHC2
				SLC35A2
				OPN1LW
				NOP56
				MTHFD1
				SLC5A1
				PATZ1
				EZR
				NLRC4
				USP48
				LYZ
				MAPKAPK5
				DOCK9
				TOLLIP

				NFKB2
				PAX2
				TCF3
				GPKOW
				HDAC4
				CALCRL
				PPP1R12A
				MYOC
				FHL1
				ZNF263
				ITGAL
				PDK4
				HOXC4
				SIRT7
				HOXC5
				GEM
				CYP3A4
				PDE9A
				BRD4
				ERBB2
				SPX
				REEP5
				HOXC11
				TJP1
				PHEX
				BDKRB1
				HOXA9
				TNFRSF12A
				FOXO6
				PTPN6
				UBE2D1
				CHRNA5
				TIMD4
				CDIPT
				MXD1
				GPX3
				TMEM134
				CDS1
				BMP10
				EBI3
				FUS
				XRCC1
				MMEL1
				HIST1H2BJ
				SP2
				HSPB8
				SLC14A1

				EMD
				PAEP
				SARS
				PARG
				BPIFB4
				SMC2
				HTR1B
				F13A1
				GSR
				BID
				HLA-DRA
				RXRG
				KLHDC3
				ARC
				PCSK1
				SLC25A32
				SLC25A14
				CHRNA3
				SRI
				NKAPL
				SNTB2
				MSX1
				NPPB
				CHRNB4
				P2RX1
				BIRC5
				CHIC2
				MBD2
				IFI30
				UCP3
				TPM1
				SAG
				EREG
				SSBP1
				JAK3
				PGRMC1
				SLC25A43
				LYN
				SERGEF
				SIRT5
				BTK
				CHM
				TNFSF9
				AGL
				DIRAS3
				FANCC
				ADAMTS12

				BRCA2
				QPCT
				TFG
				CASP5
				MAP3K7
				MATN2
				KDM5B
				PCDH9
				SPTLC3
				AS3MT
				ERC2
				IGF2R
				IRF7
				RBM10
				GYPA
				CCNA2
				SLC27A2
				IL15RA
				CCNT1
				TCP1
				PLA2G4A
				POLR2I
				PIAS4
				HMOX2
				PQBP1
				IL2RB
				SHC1
				VSTM2B
				HOXA10
				TUBA4B
				FHIT
				ZNF609
				GPR35
				SUMO4
				MGMT
				P2RY1
				RNASE6
				TBX20
				NPHS1
				GDF6
				G3BP1
				PIK3R1
				CCDC62
				TUBA4A
				SERPINA7
				GJA3
				HOXA6

				USF2
				LACTB
				WNT8B
				MYOM2
				IL23A
				CYP3A5
				PPM1A
				NGFR
				CXCR5
				TBC1D30
				BMP1
				ATP2B4
				ANKS1A
				TSBP1
				STIM2
				ALPK1
				HSD17B6
				ATG7
				NTF3
				AC010330.1
				MECP2
				SAMSN1
				XCL1
				ITGAX
				PDX1
				CCNG2
				CCNA1
				CD93
				TNFRSF10B
				UBR5
				CERK
				PES1
				IL4R
				SCN5A
				CCDC88B
				SLC2A9
				CSMD1
				CDKN2B-AS1
				DYRK1A
				TTC3
				APELA
				IL10RB
				TRIM31
				FCAR
				GBP6
				ABRA
				ATF7IP

				IFT122
				MORC3
				CBR1
				TMEM50B
				HSPA12B
				PF4V1
				RELB
				TTN
				HLA-DRB1
				PRRC2A
				NBPF3
				TYR
				MLANA
				GPC5
				FGFR2
				DOCK3
				C1GALT1
				ABCB11
				G6PC2
				ERN1
				THADA
				LBH
				RYR2
				TMPRSS6
				NRDE2
				CTSH
				BIN2
				PRIM1
				1-Mar
				SPESP1
				LPAR3
				GJB1
				DMTN
				KCNMA1
				TRPM8
				SNRPG
				POLG
				SKIL
				TTLL5
				ABCB4
				EXTL2
				BTD
				TNFRSF11A
				EPDR1
				ADGRL3
				REST
				RFTN1

				DSG1
				IL26
				PRKG2
				SLC28A1
				POLR1D
				CDH12
				KCNQ1
				ANTXR2
				GLIS3
				RGS6
				BAG6
				SF3B4
				CRACR2A
				SLC45A2
				CHEK2
				SLC46A3
				NUBPL
				RTEL1
				BCL11A
				POP1
				MIR137
				PZP
				ZNF433
				SLC6A1
				OCM2
				SDK2
				TSEN15
				HLA-DOA
				HLA-DQB1
				ZFP64
				CDKL1
				NHEJ1
				DLEU7
				SLC14A2
				MROH2A
				LMX1B
				TTLL7
				ATL1
				ARL5B
				HMG20A
				SLC22A11
				TP53INP1
				DGKD
				FRMD6
				CLEC16A
				ADGRL1
				C15orf32

				CENPC
				JMJD1C
				CEP85L
				NLRP11
				PRB2
				STARD3NL
				PBX2P1
				OR5H2
				SLC22A1
				TNPO3
				NUP205
				MPC2
				RNU7-62P
				CMIP
				MTCH2
				ELMO1
				DCC
				ZNF330
				CACNB2
				KCNC4
				SOCS6
				C1QTNF7
				ILKAP
				BARD1
				CDC42BPB
				TCHHL1
				KRT18P32
				POLR2A
				C5orf56
				TAB1
				DCP1B
				PKNOX2
				DUSP14
				TNRC6B
				FADS3
				IKZF2
				TIMM23
				CMTM8
				IRX4
				ANKLE1
				SHISA9
				ACSM1
				RAB37
				SEMA3C
				RBM38
				CDCA7L
				CASC15

				MAP2K5
				MCCC1
				TGM5
				SLTM
				CTNND2
				ADH7
				CD58
				IL12A
				MICAL3
				LINGO1
				KLHDC8B
				HLA-DQA2
				MSH5
				AK4
				CHL1
				CRTC1
				CDK10
				UNC13C
				SYT17
				TFF1
				API5
				ZNF385D
				CPVL
				GLYATL3
				OSBPL1A
				KIRREL3
				MYH7B
				TNNT3
				HNF1B
				HTR4
				XPO7
				ADAMTS20
				KCTD2
				MICU3
				ANKRD55
				MTMR3
				BAZ1B
				BTBD9
				BCAP29
				OR2M7
				HLA-A
				DPP6
				ZNF536
				LMO4
				PHF21A
				SLC22A12
				TAGAP

				PRC1
				BEST1
				PDE2A
				BCO1
				CEP112
				DCSTAMP
				HLA-C
				MPPED2
				CGNL1
				ZNF462
				RFC3
				IBSP

Supplementary Table 2:

Lists of genes that HBPs associated with myocardial Ischemia, myocardial infarction, myocarditis and atherosclerosis

Myocardial Ischemia	Myocardial infarction	Myocarditis	Atherosclerosis
TIMP3	VTN	TGM2	VTN
TGM2	TIMP3	TG	TIMP3
SOD3	SORT1	PRL	TGM2
SOD1	SOD3	PLAU	TGFB2
SHH	SOD1	MPO	TFRC
SCN5A	SCN5A	MBP	SORT1
SAA1	SAA2	LGALS9	SOD3
PRL	SAA1	ITGA1	SHH
PON2	PRL	IL5	SAA2
PLG	PON2	IL4	SAA1
PLAT	PLG	IL3	PRL
PECAM1	PLAU	IL2	PRDX4
MPO	PLAT	IL10	PON3
MMP14	PECAM1	IFNG	PON2
MET	MPO	HMGB1	PLG
LDLR	MMP14	GDNF	PLAU
INHBA	MET	F2	PLAT
IL4	LIFR	CXCL8	PLA2G5
IL2	LGALS9	CXCL12	PECAM1
IL10	LDLR	CP	OCLN
IHH	LAMA3	APOE	MPO
IFNG	ITIH3		MMP14
HSPG2	ITGA1		LGALS9
HMGB1	INSR		LDLR
GSN	INHBA		INSR
FGF5	IL7		INHBA
F2	IL5		IL7
ELN	IL4		IL5
CXCL8	IL2		IL4
CXCL12	IL10		IL3
CTSG	IHH		IL2
COMT	IFNG		IL10
APOE	HSPG2		IFNG
APOB	HRG		HSPG2
	HP		HP
	HMGB1		HMGB1
	GSN		GSN
	GDNF		GDNF
	GC		GC
	FST		FST

	FGF9		FGB
	FGF16		FGA
	FGB		F2
	FGA		F12
	FETUB		F11
	F2		F10
	F12		ELN
	F11		ECE1
	F10		CXCL8
	ELN		CXCL12
	ECE1		CTSG
	CXCL8		CPB2
	CXCL12		CP
	CTSG		COMT
	CPB2		COMP
	CP		COL4A2
	COMT		COL4A1
	COL5A1		COL1A2
	COL4A2		CDH8
	COL4A1		BTC
	COL3A1		ARG1
	COL1A2		APOE
	COL1A1		APOB
	COL18A1		ABCG5
	C9		AAMP
	AZU1		
	ARG1		
	APOE		
	APOB		
	ADRA1B		
	ABCG5		

Supplementary Table 3:

Lists of genes of non-HBPs associated with myocardial Ischemia, myocardial infarction, myocarditis and atherosclerosis

Myocardial Ischemia	Myocardial infarction	Myocarditis	Atherosclerosis
ABCA1	1-Mar	ABCD1	1-Mar
ABCC6	A2M	ACAT2	A2M
ABCC8	ABCA1	ACE	ABCA1
ABO	ABCA7	ACE2	ABCA12
ACADVL	ABCB1	ACHE	ABCA2
ACE	ABCC6	ACLY	ABCA5
ACE2	ABCC8	ACP5	ABCA7
ACHE	ABCC9	ADAM17	ABCB1
ACR	ABCD1	ADAM9	ABCB11
ACVR2B	ABCG1	ADAMTS1	ABCB4
ADA	ABCG2	ADAR	ABCB6
ADAMTS13	ABL1	ADRB1	ABCC1
ADI1	ABO	ADRB2	ABCC6
ADM	AC010330.1	AFP	ABCC8
ADM2	ACADM	AGTR1	ABCD1
ADORA1	ACADS	AHR	ABCD2
ADORA2B	ACD	AIM2	ABCE1
ADRB1	ACE	AKT1	ABCG1
ADRB2	ACE2	AKTIP	ABCG4
ADRB3	ACHE	ALB	ABCG8
AGGF1	ACKR2	ALOX5	ABHD2
AGT	ACKR3	ALPK1	ABL1
AGTR1	ACOX1	ANG	ABO
AGTR2	ACP5	ANGPT1	ABR
AGXT	ACPP	ANPEP	ABRA
AHR	ACR	ANXA5	AC010330.1
AHSG	ACSL1	ANXA6	ACADS
AHSP	ACSL6	APEX1	ACD
AIF1	ACSM3	APLN	ACE
AIFM1	ACTA2	APLNR	ACE2
AK2	ACTB	AQP4	ACER1
AK3	ACTG1	AR	ACHE
AKIP1	ACVR1B	ARSA	ACKR1
AKR1B1	ACVRL1	ASGR1	ACKR3
AKT1	ADA	ATF4	ACLY
ALB	ADAM10	ATP2A2	ACOT7
ALDH2	ADAM15	AVP	ACOX1
ALOX15	ADAM17	B2M	ACP1
ALOX5	ADAM8	BAG3	ACP5
ANG	ADAMDEC1	BCHE	ACPP
ANGPT1	ADAMTS1	BCKDK	ACR
ANGPT2	ADAMTS13	BCL2L12	ACSL1

ANGPTL3	ADAMTS2	BCL6	ACSL4
ANK1	ADAMTS4	BMP2	ACSM1
ANO1	ADAMTS7	BMPR2	ACSM3
ANPEP	ADAMTS8	BTG3	ACTA2
ANXA1	ADCY10	C3	ACTB
ANXA5	ADCY3	C5	ACTG1
ANXA6	ADCY5	C5orf38	ACTL7B
AOC3	ADCY6	CA4	ACVRL1
AOPEP	ADD1	CABIN1	ADA
APAF1	ADGB	CADM1	ADAM10
APLN	ADH1C	CALCR	ADAM12
APLNR	ADI1	CALU	ADAM15
APOA1	ADIPOQ	CASP1	ADAM17
APP	ADIPOR1	CASP3	ADAM33
AQP1	ADIPOR2	CASP8	ADAM8
AQP4	ADK	CASP9	ADAM9
ARC	ADM	CAST	ADAMTS1
AREG	ADM2	CAT	ADAMTS12
ARID3A	ADORA1	CAV1	ADAMTS13
ARSA	ADORA2A	CCL11	ADAMTS18
ASGR1	ADORA2B	CCL2	ADAMTS20
ASGR2	ADORA3	CCL20	ADAMTS4
ASIC2	ADRA1A	CCL26	ADAMTS5
ASIC3	ADRA1D	CCL27	ADAMTS7
ATF3	ADRA2A	CCL3	ADARB1
ATF4	ADRA2B	CCL4	ADARB2
ATF6	ADRB1	CCL5	ADCY10
ATG3	ADRB2	CCL7	ADCY8
ATP5IF1	ADRB3	CCN1	ADCY9
AVP	AFM	CCN2	ADCYAP1
AVPR2	AFP	CCR1	ADD1
BAMBI	AGBL2	CCR2	ADGRE2
BCHE	AGER	CCR3	ADGRE5
BCL2	AGGF1	CCR4	ADGRL1
BCL2A1	AGL	CCR5	ADGRL3
BCL2L11	AGPAT1	CCR7	ADH7
BCLAF1	AGRN	CCR8	ADHFE1
BDKRB2	AGT	CD14	ADI1
BDNF	AGTR1	CD163	ADIPOQ
BECN1	AGTR2	CD19	ADIPOR1
BMP2	AGXT	CD2	ADIPOR2
BMP4	AGXT2	CD209	ADK
BNIP3	AHSG	CD27	ADM
BRD1	AHSP	CD274	ADM2
BSG	AIDA	CD276	ADORA1
C1QTNF1	AIFM1	CD28	ADORA2A
C5AR1	AIRE	CD34	ADORA2B

C5orf38	AK1	CD38	ADORA3
CA1	AKAP1	CD3D	ADRB1
CABIN1	AKAP12	CD4	ADRB2
CACNA1C	AKAP6	CD40	ADRB3
CACNA1D	AKIP1	CD40LG	ADTRP
CACNA1F	AKR1B1	CD44	AEBP1
CACNA1S	AKT1	CD55	AFM
CADM1	AKT2	CD68	AGER
CALCRL	ALAS2	CD69	AGGF1
CALR	ALB	CD70	AGL
CASC1	ALCAM	CD72	AGT
CASP1	ALDH1A2	CD80	AGTR1
CASP2	ALDH2	CD83	AGTR2
CASP3	ALG1	CD86	AGXT
CASP8	ALK	CDK9	AGXT2
CASP9	ALMS1	CDKN2A	AHCY
CASR	ALOX12	CEACAM5	AHI1
CAST	ALOX15	CEBPA	AHR
CASZ1	ALOX5	CEBPB	AHRR
CAT	ALOX5AP	CENPA	AHSG
CAV1	ALPI	CFD	AHSP
CAV3	ALPK1	CFLAR	AIF1
CBFA2T2	AMELX	CFP	AIFM1
CCL18	AMH	CGAS	AIM2
CCL19	AMIGO2	CHST15	AIRE
CCL2	AMN	CHUK	AK4
CCL20	AMPD1	CIITA	AKAP12
CCL5	AMT	CISH	AKAP13
CCL7	AMY1C	CLU	AKR1B1
CCN1	ANAPC5	CMA1	AKT1
CCN2	ANG	CNOT7	AKT2
CCR2	ANGPT1	CNP	AKTIP
CCR5	ANGPT2	CNR1	ALB
CCS	ANGPTL2	CNR2	ALCAM
CD151	ANGPTL3	CORT	ALDH2
CD180	ANGPTL4	CPE	ALG1
CD274	ANK2	CR1	ALOX12
CD34	ANK3	CR2	ALOX15
CD36	ANPEP	CRP	ALOX15B
CD38	ANTXR2	CSDE1	ALOX5
CD4	ANXA1	CSF1	ALOX5AP
CD40	ANXA13	CSF1R	ALPK1
CD40LG	ANXA2	CSF2	ALX1
CD47	ANXA3	CSF3	AMD1
CD59	ANXA5	CSRP3	AMH
CDH5	ANXA7	CTF1	AMOT
CDK2	AOC1	CTH	AMPD1

CDK9	AOC2	CTLA4	ANG
CDKN1A	AOC3	CTSB	ANGPT1
CDKN2A	AOPEP	CTSK	ANGPT2
CDS1	AP3D1	CX3CL1	ANGPTL2
CEBPZ	APCS	CX3CR1	ANGPTL3
CETP	APELA	CXADR	ANGPTL4
CFB	APEX1	CXCL1	ANGPTL6
CFI	APLN	CXCL10	ANGPTL8
CFTR	APLNR	CXCL13	ANK1
CHI3L1	APOA1	CXCL9	ANKLE1
CHUK	APOA2	CXCR3	ANKRD55
CIRBP	APOA4	CXCR5	ANKS1A
CLCN2	APOA5	CYBB	ANKS1B
CLEC6A	APOBEC3G	CYCS	ANO3
CLEC7A	APOC1	CYP11B2	ANPEP
CLU	APOC2	DDIT3	ANTXR2
CMA1	APOC3	DDX53	ANXA1
CMKLR1	APOD	DES	ANXA13
CNP	APOH	DHX58	ANXA2
CNPY2	APOL1	DLD	ANXA4
CNR1	APOM	DMD	ANXA5
CNR2	APP	DNTT	ANXA7
COMMD1	AQP1	DSP	AOC1
CPA1	AQP2	DYSF	AOC2
CPB1	AQP3	EBI3	AOC3
CPOX	AQP4	EDN1	AOPEP
CPSF4	AR	EDNRA	AP2A2
CR1	ARC	EIF2AK2	AP5Z1
CRBN	AREG	EIF4E	APAF1
CREB3	ARID3A	EIF4EBP1	APCS
CREG1	ARIH1	EMB	APEH
CRH	ARNT	EMP1	APELA
CRP	ARNTL	EPAS1	APEX1
CRYAB	ARRB1	EPHA3	APH1B
CSF2	ARSA	EPHB2	API5
CSF3	ARSB	EPO	APLN
CTH	ARSI	ESR1	APLNR
CTNNB1	ART3	ESR2	APOA1
CTSB	ASAH2	F2R	APOA2
CTSD	ASF1A	F2RL1	APOA4
CTSL	ASGR1	F3	APOA5
CX3CL1	ASIC3	F8	APOBEC1
CXCL1	ASRGL1	FASLG	APOBEC3G
CXCL16	ATF3	FGF2	APOBR
CXCL17	ATF6	FGF23	APOC1
CXCR2	ATF7IP	FGL2	APOC2
CXCR4	ATG7	FIP1L1	APOC3

CXCR6	ATM	FLNA	APOC4
CYBB	ATP10A	FLT3LG	APOD
CYCS	ATP10D	FN1	APOF
CYP2E1	ATP1B1	FOS	APOH
CYP2J2	ATP2A2	FOXP3	APOL1
CYSLTR2	ATP2B1	FUT4	APOM
DAXX	ATP6AP2	FUT7	APP
DDAH1	ATP7A	FYN	APPL1
DDIT3	ATP8A2	G6PD	AQP1
DES	ATXN1	GAPDH	AQP7
DIAPH1	ATXN2	GBP1	AR
DKK1	ATXN7L3B	GH1	ARC
DLL1	AURKB	GJA1	ARG2
DMD	AVP	GJC1	ARHGAP18
DNAH5	AVPR1A	GLB1	ARHGEF26
DNASE1	AVPR2	GOLGA2	ARID5B
DNM1L	AXDND1	GP5	ARL5B
DPP4	AXL	GPI	ARL6IP4
DRD2	AZIN2	GPX1	ARNTL
DSE	B2M	GSK3B	ARRB1
DST	B3GNT2	GZMB	ARRB2
DTNA	BAG3	HAMP	ARSA
DYSF	BAX	HBEGF	ARSB
EDN1	BBOX1	HDAC11	ARSI
EDNRA	BBS9	HDAC2	AS3MT
EDNRB	BCAS3	HECA	ASAH2
EEF2	BCHE	HGF	ASCL1
EFNA1	BCL2	HIF1A	ATF1
EGLN1	BCL2A1	HIST1H4L	ATF2
EGLN3	BCL2L11	HLA-DQB1	ATF3
EGR1	BCLAF1	HMGCR	ATF4
EIF2AK1	BCR	HMGXB3	ATF7IP
EIF2AK2	BDKRB1	HMOX1	ATG13
EIF2S1	BDKRB2	HNF1A	ATG16L1
EIF5A	BDNF	HPRT1	ATG5
ELANE	BECN1	HRH1	ATG7
ELOVL6	BGLAP	HSF1	ATIC
EMD	BGN	HSP90B1	ATL1
EMP1	BICRA	HSPA5	ATM
ENPEP	BID	HTR3A	ATP10A
ENTPD1	BIN1	HTT	ATP10D
EPAS1	BLVRB	HUNK	ATP2B1
EPHB2	BMF	ICAM1	ATP2B4
EPHB4	BMI1	ICOS	ATP7A
EPO	BMP1	ICOSLG	ATXN2
EPOR	BMP10	IDO1	AURKA
EPRS	BMP2	IDS	AVP

ESD	BMP4	IFIH1	AVPR2
ESR1	BMP6	IFNA1	AXL
ESR2	BMP7	IFNA2	B2M
ETV3	BMPER	IFNA6	BACE1
F2R	BNC1	IFNB1	BACH2
F2RL1	BNIP3	IFNL2	BAG3
F3	BNIP3L	IL12RB1	BAG6
F7	BOK	IL13	BAMBI
F8	BRAF	IL15	BARD1
FABP3	BRAP	IL17A	BAX
FABP4	BRCA1	IL17D	BAZ1B
FADD	BRD1	IL17RA	BCAP29
FAM168B	BRD4	IL18	BCHE
FAP	BRINP3	IL18BP	BCL11A
FASLG	BSG	IL1A	BCL2A1
FCER1G	BTF3	IL1B	BCL2L11
FDPS	BTG2	IL1R1	BCL3
FGF1	BTG3	IL1RN	BCL6
FGF19	BTN2A1	IL21	BCLAF1
FGF2	C10orf142	IL21R	BCO1
FGF21	C1QTNF1	IL22	BCR
FGF23	C1QTNF3	IL32	BDKRB1
FGF4	C3	IL33	BDKRB2
FGFR1	C3AR1	IL37	BDNF
FGFR2	C4A	IL6	BECN1
FGFR3	C4B	IL9	BEST1
FGFR4	C4BPA	ILK	BGLAP
FGL2	C4BPB	INS	BGN
FHL2	C5	IRAK1	BID
FN1	C5AR1	IRAK4	BIN2
FOS	C5orf38	IRF1	BIRC2
FOXP3	C6orf15	IRF3	BIRC5
FPR2	CA1	IRF4	BIRC6
FSTL1	CA3	IRF7	BLVRA
FXYD1	CA4	IRF9	BLVRB
FZD2	CA5B	ISG15	BMF
G6PD	CA6	ITGA6	BMP1
GAPDH	CACNA1B	ITK	BMP10
GAST	CACNA1C	IVNS1ABP	BMP2
GATA4	CACNA1D	JAK1	BMP4
GCH1	CACNA1F	JAK2	BMP7
GDF11	CACNA1S	JAK3	BMP8A
GDF15	CACNA2D2	JUN	BMPER
GFAP	CACNB3	KCNN4	BMPR1A
GH1	CACYBP	KLF2	BMPR2
GHRL	CADM1	KLKB1	BPIFB4
GHSR	CALB1	KLRD1	BRAP

GJA1	CALB2	KLRK1	BRCA1
GLA	CALCR	KMO	BRCA2
GLB1	CALD1	LAG3	BRD1
GLP1R	CALR	LCN2	BRD4
GLRX3	CAMKK1	LDB3	BRD7
GOT2	CAMP	LDHA	BRINP3
GP6	CANX	LEP	BSCL2
GPER1	CAPN10	LEPR	BSG
GPI	CARD17	LGALS1	BTBD9
GPX1	CARD8	LGALS3	BTD
GRAP2	CARD9	LRPAP1	BTG3
GRK2	CASC1	LTA	BTK
GRN	CASP1	LY6E	BTNL2
GSC	CASP12	MAEA	C10orf113
GSDMD	CASP3	MALT1	C15orf32
GSK3B	CASP8	MAPK3	C1GALT1
GUCY1B1	CASP9	MATN1	C1QL3
GUCY1B2	CASR	MB	C1QTNF1
GUSB	CAST	MBL2	C1QTNF12
GZMK	CAT	MBTPS1	C1QTNF3
HAMP	CAV1	MCIDAS	C1QTNF5
HBD	CAV3	MDK	C1QTNF6
HDAC4	CAVIN4	MEFV	C1QTNF7
HDAC5	CBFA2T2	MIF	C3
HDAC6	CBS	MLYCD	C4A
HDGF	CBX1	MMP1	C4BPA
HGF	CBY1	MMP12	C5AR1
HGS	CCAR1	MMP2	C5AR2
HIF1A	CCDC60	MMP3	C5orf38
HIF3A	CCDC63	MMP8	C5orf56
HMGB2	CCDC88A	MMP9	C6orf118
HMGCR	CCK	MMRN1	CA1
HMOX1	CCL11	MMUT	CA6
HMOX2	CCL16	MOG	CAAP1
HPGDS	CCL18	MPG	CABIN1
HPS1	CCL19	MPI	CACNA1C
HRC	CCL2	MS4A1	CACNA1D
HRH2	CCL20	MSC	CACNA1F
HRH3	CCL21	MSN	CACNA1S
HSF1	CCL22	MTOR	CACNB2
HSP90AB1	CCL27	MYH6	CADM1
HSPA12B	CCL28	MYH7	CADPS
HSPA5	CCL3	MYL3	CALCR
HSPA8	CCL4	MYLK	CALCRL
HSPB1	CCL5	MYOM2	CALD1
HSPB2	CCL7	NCAM1	CALR
HSPB3	CCN1	NCR1	CALU

HSPB8	CCN2	NELFCD	CAMKK2
HSPD1	CCN4	NFAT5	CAMP
HTR1B	CCNA2	NGF	CAMSAP3
HTR2A	CCND2	NLRP3	CAPN10
HTR3A	CCR1	NMT1	CAPN6
HTRA2	CCR2	NOD2	CARD10
HYOU1	CCR3	NOS2	CARD16
ICAM1	CCR4	NOS3	CARD8
IFI44	CCR5	NPM1	CARD9
IFIT2	CCR7	NPPA	CARHSP1
IGF1	CCR9	NPPC	CASC15
IGF2	CCS	NR3C1	CASP1
IGFBP4	CCT7	NR3C2	CASP3
IKBKB	CD14	NTF4	CASP5
IL17A	CD151	OGN	CASP8
IL18	CD163	P2RX7	CASP9
IL18BP	CD180	PCNA	CASR
IL1A	CD19	PDCD1	CAST
IL1B	CD2	PDCD4	CAT
IL33	CD209	PDGFC	CAV1
IL37	CD27	PDGFRA	CAV2
IL6	CD274	PDLIM7	CAV3
IL6ST	CD28	PI3	CAVIN3
ILF2	CD34	PIK3CG	CBFA2T2
ILK	CD36	PKP2	CBL
INS	CD38	PLA2G1B	CBLIF
IRAK1	CD4	PLP1	CBLN1
IRAK4	CD40	PNKD	CBR1
IRF9	CD40LG	POR	CBS
ITGA2B	CD44	POSTN	CCAR1
ITGAM	CD47	PPARA	CCAR2
ITGB2	CD5	PPARG	CCDC57
ITGB3	CD55	PPIA	CCDC62
ITLN1	CD59	PPP1R15A	CCDC7
ITPR2	CD5L	PPP5C	CCDC80
JAK1	CD6	PRH1	CCDC88B
JAK2	CD63	PRKCA	CCK
JUN	CD68	PRKCB	CCL11
KARS	CD69	PROM1	CCL13
KCNH2	CD7	PRSS2	CCL15
KCNMA1	CD70	PRYP4	CCL16
KCNQ1	CD80	PSMB8	CCL17
KDR	CD86	PSME1	CCL18
KIF2A	CD93	PSME2	CCL19
KITLG	CD96	PTEN	CCL2
KL	CDA	PTGS2	CCL20
KLF15	CDC73	PTPRC	CCL21

KLF5	CDCA7L	PVR	CCL22
KLK1	CDCP1	RAG1	CCL23
KLKB1	CDH1	RAPGEF5	CCL24
KRAS	CDH12	RBM38	CCL25
KRT1	CDH13	REN	CCL26
LCN2	CDH2	RHD	CCL27
LDHB	CDH5	RIPK1	CCL28
LEP	CDK5RAP1	RIPK3	CCL3
LGALS3	CDK9	RNASE3	CCL4
LIPC	CDKAL1	RNPC3	CCL5
LOX	CDKN1A	RPL17	CCL7
LPA	CDKN1B	RPTOR	CCL8
LPCAT3	CDKN1C	RYR2	CCN1
LPL	CDKN2A	S100A8	CCN2
LPO	CDKN2B	S100A9	CCN3
LRP1	CDKN2B-AS1	SARS	CCN4
LRP6	CDS1	SCARA3	CCN5
LTA	CEACAM1	SCD	CCNA1
LTB4R	CEACAM5	SELE	CCNA2
LY86	CEACAM6	SELL	CCNG2
MAEA	CEBPB	SEMA4A	CCNT1
MAMLD1	CEBPZ	SEMA7A	CCR1
MANF	CELF1	SERPINA5	CCR2
MAP2K1	CELF4	SERPINC1	CCR3
MAP2K3	CELSR2	SERPINE1	CCR4
MAP3K5	CENPJ	SGCD	CCR5
MAPK1	CEPT1	SLC17A5	CCR6
MAPK14	CES1	SLC25A10	CCR7
MAPK3	CETP	SLC25A4	CCR8
MAPK7	CFB	SLC33A1	CCR9
MAPK8	CFD	SLC7A11	CCS
MAPKAPK2	CFH	SMAD2	CCT4
MASP2	CFI	SMAD3	CD14
MAVS	CFL1	SOAT1	CD151
MB	CFLAR	SOCS1	CD160
MBL2	CFP	SOCS3	CD163
MBTPS1	CFTR	SORBS1	CD180
MC3R	CGAS	SPARC	CD19
MCAT	CGN	SPP1	CD2
MCF2L	CHAT	SPX	CD209
MEFV	CHD1L	SRSF2	CD24
MFN1	CHGA	SSB	CD248
MFN2	CHGB	SST	CD27
MICU1	CHI3L1	STAT1	CD274
MIF	CHIC2	STAT2	CD276
MLYCD	CHP1	STAT3	CD28
MMD	CHRDL1	STAT4	CD34

MME	CHRFAM7A	STAT6	CD36
MMP1	CHRM2	SULT1E1	CD37
MMP2	CHRNA5	TACR1	CD38
MMP3	CHUK	TACR3	CD4
MMP9	CIART	TBCA	CD40
MMRN1	CIC	TDP2	CD40LG
MPG	CIITA	TFPI	CD44
MPI	CILP	TGFB1	CD47
MRC1	CITED4	THBS1	CD5
MRRF	CKB	THBS2	CD53
MRTFA	CKLF	TICAM1	CD55
MSC	CKM	TIMP1	CD58
MSLN	CKMT2	TLR2	CD59
MSTN	CLCN1	TLR3	CD5L
MTHFR	CLEC16A	TLR4	CD6
MT-ND2	CLEC3B	TLR7	CD63
MTOR	CLEC6A	TLR9	CD68
MT-RNR2	CLEC7A	TMPRSS2	CD69
MVD	CLK1	TNC	CD70
MYC	CLMN	TNF	CD74
MYOCD	CLSTN2	TNFAIP3	CD79A
NAMPT	CLU	TNFRSF11B	CD80
NCAM1	CMA1	TNFRSF14	CD81
NCL	CMIP	TNFRSF18	CD86
NDRG2	CNP	TNFRSF1A	CD9
NDRG4	CNPY2	TNFRSF1B	CD93
NES	CNR1	TNFRSF25	CD99
NF2	CNR2	TNFRSF8	CD99L2
NFAT5	CNTF	TNFSF11	CDC27
NFE2L2	CNTN2	TNFSF4	CDC42
NFKBIA	CNTN4	TNFSF8	CDC42BPB
NGB	COG2	TNFSF9	CDC42EP3
NGF	COMMD7	TNNI3	CDC6
NKX2-5	COPE	TNNT2	CDCA7L
NLRP1	CORIN	TOR1A	CDH1
NLRP3	CORO1A	TRAF6	CDH10
NLRX1	CORT	TREX1	CDH12
NOC2L	CPA1	TRIM21	CDH13
NOD1	CPB1	TRIM63	CDH5
NOD2	CPM	TSPO	CDIPT
NOS1	CPNE3	TTN	CDK1
NOS2	CPOX	TTR	CDK10
NOS3	CPSF4	TXN	CDK2
NOTCH1	CPT1B	UCHL1	CDK4
NOTCH3	CPT2	VCAM1	CDK5
NOX1	CR1	VCL	CDK9
NOX4	CREB3L1	VDR	CDKAL1

NPPA	CREG1	VEGFA	CDKL1
NPPC	CREM	VIM	CDKN1A
NPR2	CRH	VIP	CDKN1B
NPY	CRHR2	XCL1	CDKN1C
NPY2R	CRIP1	XPR1	CDKN2A
NR1H2	CRK	ZBTB12	CDKN2A-DT
NR1H3	CRLS1	ZC3H12A	CDKN2B
NR1H4	CRP	ZC3HAV1	CDKN2B-AS1
NR3C2	CRX		CDS1
NTHL1	CRY2		CEACAM5
NTN1	CRYAB		CEACAM6
NTRK1	CSAD		CEBPA
NTRK2	CSF1		CEBPB
NTS	CSF1R		CEBPD
NUCB2	CSF2		CEBPZ
NUDT1	CSF3		CEL
NUP43	CSF3R		CELA1
OGA	CSRP3		CELSR1
OGG1	CST3		CELSR2
OLR1	CTF1		CENPC
OPA1	CTLA4		CENPJ
OPHN1	CTNNB1		CEP112
OPN1LW	CTSA		CEP152
OPRM1	CTSB		CEP85L
OSM	CTSC		CERK
P2RX2	CTSD		CERS5
P2RX3	CTSK		CES1
P2RX4	CTSL		CETP
P2RX7	CTSS		CFD
P2RY11	CUEDC2		CFH
P2RY12	CUL7		CFLAR
PADI4	CX3CL1		CFP
PAPPA	CX3CR1		CFTR
PARK7	CXADR		CGNL1
PARP1	CXCL1		CHAC1
PBK	CXCL10		CHAF1A
PBRM1	CXCL16		CHEK2
PCBD1	CXCL3		CHGA
PCSK5	CXCL9		CHI3L1
PCSK7	CXCR1		CHI3L2
PCYT1A	CXCR2		CHIC1
PDC	CXCR3		CHIC2
PDE10A	CXCR4		CHIT1
PDE1A	CXCR5		CHKA
PDE1B	CYBA		CHL1
PDE1C	CYBB		CHM
PDE2A	CYCS		CHP1

PDE3A	CYP11B2		CHRFAM7A
PDE3B	CYP17A1		CHRNA3
PDE4A	CYP19A1		CHRNA5
PDE4B	CYP1A1		CHRNB4
PDE4C	CYP1A2		CHST11
PDE4D	CYP2C19		CHST3
PDE5A	CYP2C8		CHUK
PDE6A	CYP2C9		CIART
PDE6B	CYP2D6		CIC
PDE6C	CYP2J2		CIDEC
PDE6D	CYP2R1		CIITA
PDE6G	CYP3A4		CISD1
PDE6H	CYP3A5		CISH
PDE7A	CYP4A11		CKLF
PDE7B	CYP4F2		CLCF1
PDE8A	CYP7A1		CLCN3
PDE8B	DAB2IP		CLDN1
PDE9A	DAO		CLEC16A
PER2	DAOA		CLEC1B
PF4	DAP		CLEC3B
PGAM2	DBH		CLEC4C
PGAM5	DBI		CLEC5A
PGF	DBT		CLEC6A
PGRMC1	DCC		CLEC7A
PHLPP1	DCD		CLIC1
PI3	DCLK2		CLIP1
PIAS1	DCN		CLSTN2
PICALM	DCTD		CLU
PIK3CB	DCTN3		CMA1
PIK3CD	DDAH1		CMIP
PIK3CG	DDAH2		CMKLR1
PKN1	DDC		CMTM8
PLA2G10	DDI1		CNMD
PLA2G1B	DDIT3		CNN2
PLA2G4A	DDR2		CNNM1
PLA2G7	DDRGK1		CNNM2
PLD1	DDX4		CNOT4
PLD6	DDX41		CNP
PLEK	DEFA1		CNR1
PLIN5	DEGS1		CNR2
PNOC	DENR		CNTN3
PNPLA2	DES		COG2
PON1	DFFA		COG6
POR	DGCR2		COL14A1
POSTN	DGKA		COL15A1
PPARA	DGKB		COL5A1
PPARD	DGKQ		COLEC12

PPARG	DGKZ		COMMD1
PPARGC1A	DGUOK		COPS5
PPIA	DHFR		CORIN
PPIF	DIAPH1		CORT
PRDX1	DIPK2A		CPA1
PRDX2	DIS3L		CPB1
PRDX5	DISC1		CPD
PRH1	DKK1		CPE
PRKAA2	DKK2		CPEB2
PRKCB	DKK3		CPN1
PSEN1	DLAT		CPSF4
PSMA2	DLG1		CPT1A
PTAFR	DMD		CPT2
PTEN	DMRTA1		CPVL
PTGS1	DNAH11		CR1
PTGS2	DNAJC5		CR2
PTH	DNASE1		CRACR2A
PTHLH	DNM1L		CREB3
PTK2	DNMT1		CREB3L3
PTN	DNMT3A		CREG1
PTPN1	DNTT		CRH
PTPRC	DOCK11		CRHR1
PVR	DOT1L		CRISP2
PXDN	DPEP1		CRLF2
RACK1	DPP4		CRP
RAF1	DPP6		CRTC1
RALA	DPPA3		CRY1
RAMP2	DPT		CSAD
RAPGEF3	DPYS		CSF1
RARRES2	DPYSL2		CSF1R
REN	DRAM1		CSF2
RETN	DRAM2		CSF2RA
RHEB	DRD1		CSF3
RIPK1	DRD2		CSF3R
RIPK3	DSE		CSGALNACT2
RIPOR2	DSG2		CSHL1
RIT1	DSG3		CSMD1
RNF146	DSP		CSMD3
RNH1	DST		CSN2
RNLS	DTNA		CST3
RRM1	DTNB		CSTB
RTCA	DUSP4		CTCF
RUNX1	DVL1		CTF1
RYR2	DYRK1A		CTH
S100A1	E2F1		CTLA4
S100A12	E2F2		CTNNB1
S100A4	E2F3		CTNND2

S100A6	ECD		CTSA
S100A8	ECHDC3		CTSB
S100B	ECM1		CTSC
S1PR1	EDA		CTSD
S1PR2	EDC4		CTSF
S1PR3	EDN1		CTSH
SAT1	EDN2		CTSK
SCD	EDN3		CTSL
SCG2	EDNRA		CTSS
SCN4A	EDNRB		CTSV
SELE	EEF1A2		CUBN
SELENOP	EEF2		CUL3
SELL	EFNA1		CUL5
SELP	EFNB2		CWC22
SELPLG	EGF		CX3CL1
SENP3	EGFR		CX3CR1
SERPINA1	EGLN1		CXCL1
SERPINA12	EGLN2		CXCL10
SERPINA4	EGLN3		CXCL11
SERPINA5	EGR1		CXCL13
SERPINC1	EIF2S1		CXCL14
SERPINE1	EIF4A3		CXCL16
SERPINF1	ELANE		CXCL2
SERPINF2	ELAVL1		CXCL5
SERPING1	ELOVL5		CXCL9
SESN2	EMC10		CXCR1
SETD2	EMD		CXCR2
SETD7	EMILIN1		CXCR3
SGK1	EMP1		CXCR4
SH3BP4	ENG		CXCR5
SHD	ENHO		CXCR6
SIRT1	ENO2		CYBA
SIRT2	ENO3		CYBB
SIRT3	ENPEP		CYCS
SIRT5	ENPP1		CYGB
SIRT6	ENTPD1		CYLD
SKIL	EPCAM		CYP11B2
SLC17A5	EPHA1		CYP17A1
SLC22A12	EPHA2		CYP19A1
SLC22A4	EPHA4		CYP1A1
SLC25A1	EPHB2		CYP1A2
SLC25A3	EPHB4		CYP1B1
SLC29A1	EPHX1		CYP21A2
SLC2A1	EPHX2		CYP26A1
SLC2A4	EPO		CYP26B1
SLC33A1	EPOR		CYP27A1
SLC5A5	EPRS		CYP27B1

SLC6A2	EPX		CYP2C19
SLC6A4	ERAL1		CYP2C8
SLC9A1	ERAS		CYP2C9
SLCO6A1	ERBB2		CYP2J2
SLN	ERBB3		CYP3A4
SLPI	ERBB4		CYP3A5
SMAD2	ERCC8		CYP7A1
SMAD3	ERFE		DAB2IP
SMAD4	ESD		DAPK3
SMAD7	ESM1		DBI
SMC2	ESR1		DBN1
SMPD1	ESR2		DBT
SOAT1	ETV2		DCC
SOCS3	ETV3		DCD
SOX7	ETV6		DCLRE1C
SP4	EVC		DCN
SPARC	EVPL		DCP1B
SPHK1	EXOSC10		DCSTAMP
SPP1	EXOSC4		DCTN3
SPRR3	EZH1		DDAH1
SRC	EZH2		DDAH2
SRY	F11R		DDC
SSB	F13A1		DDIT3
SST	F2R		DDR1
ST13	F2RL1		DDR2
ST3GAL4	F3		DDX41
ST8SIA4	F5		DDX58
STAT1	F7		DEFA1
STAT3	F8		DEFB103B
STAT5A	FAAH		DEFB4B
STAT6	FABP1		DENR
STC1	FABP2		DES
STEAP4	FABP3		DFFA
STH	FABP4		DFFB
STIL	FADD		DGAT1
STIM1	FADS1		DGKD
STK11	FADS2		DHCR24
STOM	FAIM		DHCR7
STOML2	FAIM2		DHFR
STS	FAM111B		DHRS2
SUCNR1	FANCC		DHX58
SULT1A3	FAP		DIRAS3
SULT1E1	FARP2		DISC1
SULT2A1	FARS2		DKK1
SULT4A1	FASLG		DKK3
SYNE1	FBLN1		DLAT
SYT1	FBLN2		DLEU7

TAB1	FBLN5		DLGAP2
TACR1	FBN1		DLK1
TAGLN	FBXL4		DLL4
TBCD	FBXO32		DMD
TBXA2R	FCAR		DMTN
TCF21	FCGR1A		DNAJC5
TCHP	FCGR2A		DNASE1
TCP1	FCGR2B		DNM1
TET1	FCGR3A		DNM2
TF	FCGR3B		DNMT1
TFAM	FCN1		DOCK3
TFEB	FCN2		DOCK4
TFF3	FDFT1		DOCK7
TFPI	FES		DOCK9
TGFB1	FEV		DPEP1
TGFB3	FGF1		DPP4
THRA	FGF18		DPP6
THRB	FGF2		DPP8
TICAM1	FGF21		DPP9
TIMP1	FGF23		DRD2
TIMP2	FGFR1		DSC1
TJP1	FGFR2		DSE
TLR2	FGG		DSG1
TLR3	FHL2		DST
TLR4	FKBP1A		DSTN
TLR9	FKBP1B		DUSP1
TLX1NB	FKBP5		DUSP10
TLX2	FLII		DUSP14
TMEFF2	FLNA		DUSP16
TMSB4X	FLNC		DUSP5
TNC	FLOT2		DYRK1A
TNF	FLRT2		E2F1
TNFAIP8L2	FLT1		EBF1
TNFRSF11B	FLT3		EBI3
TNFRSF1A	FLT4		ECD
TNFRSF1B	FMN1		ECE2
TNNI3	FN1		ECSCR
TNNI3K	FNDC5		EDA
TNNT1	FOS		EDAR
TNNT2	FOXE1		EDN1
TNS1	FOXG1		EDN2
TNS3	FOXH1		EDN3
TP53	FOXL1		EDNRA
TP53INP2	FOXO1		EDNRB
TPO	FOXP3		EEF2K
TPR	FPR1		EFHC1
TPT1	FRZB		EFNA1

TRIM63	FSTL1		EFNA5
TRPA1	FTO		EFNB1
TRPC1	FURIN		EFNB2
TRPM4	FXYD1		EGF
TRPM6	FZD1		EGFR
TRPM7	FZD2		EGLN1
TRPV1	G6PD		EGLN2
TRPV4	GABRA1		EGLN3
TSPO	GABRA2		EGR1
TTN	GABRA3		EHF
TTR	GABRA4		EHHADH
TWSG1	GABRA5		EIF2AK1
TXN	GABRA6		EIF2AK2
TXN2	GABRB1		EIF2S1
TXNIP	GABRB2		EIF4E
TXNL1	GABRB3		ELANE
TYMP	GABRD		ELAVL1
UBQLN1	GABRE		ELMO1
UBR5	GABRG1		EMD
UCN	GABRG2		EMP1
UCN3	GABRG3		ENG
UCP2	GABRP		ENHO
UCP3	GABRQ		ENO2
ULK1	GAD1		ENPEP
UNC5B	GAK		ENPP1
VASP	GAP43		ENPP2
VCAM1	GAPDH		ENPP7
VCL	GAS6		ENTPD1
VCP	GAST		EPAS1
VDAC1	GATA3		EPB41L4B
VDR	GATA4		EPC2
VEGFA	GBA		EPDR1
VEGFB	GCFC2		EPHA1
VEGFC	GCGR		EPHA3
VIM	GCH1		EPHB1
VIP	GCKR		EPHB2
VKORC1	GCLC		EPHX2
VPS51	GCLM		EPN1
VWF	GDA		EPO
WDR26	GDF1		ERBB2
WT1	GDF10		ERC2
XBP1	GDF11		ERCC1
XPR1	GDF15		ERCC2
YES1	GFAP		ERCC8
ZC3H12A	GFOD2		EREG
ZGLP1	GFPT1		ERICH1
	GFPT2		ERN1

	GH1		ERN2
	GHRH		ESAM
	GHRHR		ESM1
	GHRL		ESR1
	GHSR		ESR2
	GIP		ESRRA
	GIPR		ETV3
	GJA1		EVPL
	GJA4		EXTL2
	GJA5		EZH2
	GJB1		EZR
	GJC1		F11R
	GJD2		F13A1
	GLA		F2R
	GLB1		F2RL1
	GLI1		F3
	GLP1R		F5
	GLRA1		F7
	GLRA2		F8
	GLRX		FA2H
	GLUL		FABP1
	GNB3		FABP2
	GNLY		FABP3
	GNRH1		FABP4
	GOPC		FABP5
	GOSR2		FADD
	GOT2		FADS1
	GP1BA		FADS2
	GP5		FADS3
	GP6		FAM168B
	GPER1		FAM174A
	GPI		FAM49A
	GPKOW		FANCC
	GPNMB		FASLG
	GPR132		FASN
	GPR17		FBLN1
	GPR180		FBLN2
	GPR35		FBLN5
	GPR4		FBN1
	GPR65		FBP2
	GPX1		FBXO3
	GPX3		FCAR
	GRAP2		FCGR1A
	GREM2		FCGR2A
	GRIN2B		FCGR2B
	GRK2		FCGR3A
	GRK4		FCN2

	GRK5		FDFT1
	GRK6		FDPS
	GRN		FDX1
	GSC		FEV
	GSDMD		FEZ1
	GSK3A		FFAR4
	GSK3B		FGF1
	GSR		FGF12
	GSTA1		FGF19
	GSTCD		FGF2
	GSTM1		FGF21
	GSTP1		FGF23
	GUCY1A1		FGFBP1
	GUCY1B1		FGFR1
	GUCY1B2		FGFR2
	GUSB		FGFR4
	GYG1		FGG
	GYS1		FHIT
	GZMB		FHL1
	H2AFX		FHL2
	HABP2		FLCN
	HAMP		FLNA
	HARS		FLOT2
	HAS3		FLT1
	HAVCR1		FLT4
	HBD		FMO3
	HBEGF		FMOD
	HCAR2		FN1
	HCN2		FOLR1
	HCN4		FOLR2
	HCRT		FOS
	HDAC4		FOSL1
	HDAC5		FOXO1
	HDAC6		FOXO3
	HDAC9		FOXO6
	HDGF		FOXP1
	HEMGN		FOXP3
	HFE		FPR1
	HGF		FPR2
	HGS		FPR3
	HHIPL1		FRMD4A
	HIF1A		FRMD6
	HIGD1A		FTL
	HIP1		FTO
	HK1		FURIN
	HK2		FUS
	HLA-DMA		FUT4

	HLA-DQB1		FUT7
	HLA-DRA		FXYD1
	HM13		FYN
	HMBS		G0S2
	HMCN1		G3BP1
	HMGA1		G6PC2
	HMGB2		G6PD
	HMGCR		GABRA1
	HMMR		GABRA2
	HMOX1		GABRA3
	HNF1A		GABRA5
	HNF1B		GABRG1
	HNRNPD		GABRG2
	HNRNPUL1		GABRG3
	HPGDS		GABRR3
	HPR		GAD1
	HPS1		GADD45A
	HPSE		GALNT3
	HPX		GALNT4
	HRC		GAPDH
	HRH3		GAS6
	HSD11B1		GATA1
	HSF1		GATA2
	HSP90AB1		GATA4
	HSP90B1		GATA6
	HSPA12B		GBP6
	HSPA1A		GCGR
	HSPA5		GCH1
	HSPA8		GCK
	HSPB1		GCKR
	HSPB3		GCLC
	HSPB7		GCLM
	HSPB8		GDF10
	HSPD1		GDF11
	HTR1B		GDF15
	HTR1D		GDF2
	HTR2A		GDF6
	HTR3A		GEM
	HTR4		GET4
	HTRA2		GH1
	HUNK		GHR
	HYOU1		GHRHR
	IARS		GHRL
	ICAM1		GHSR
	ICAM3		GIP
	ICOS		GIPR
	IDO1		GJA1

	IFIT1		GJA3
	IFNB1		GJA4
	IFNGR1		GJA5
	IFNGR2		GJB1
	IFNL1		GJB5
	IFNLR1		GJC2
	IGF1		GLA
	IGF1R		GLB1
	IGF2		GLG1
	IGF2R		GLIS3
	IGFBP1		GLO1
	IGFBP3		GLP1R
	IGFBP4		GLRX3
	IGFBP7		GLYATL3
	IGHM		GMNN
	IK		GNA11
	IKBKB		GNA12
	IL10RA		GNA13
	IL11		GNB3
	IL12B		GNMT
	IL13		GNRHR
	IL15		GOSR2
	IL17A		GOT1
	IL17D		GOT2
	IL17F		GP1BA
	IL18		GP5
	IL18BP		GP6
	IL19		GPBAR1
	IL1A		GPBP1
	IL1B		GPC1
	IL1F10		GPC5
	IL1R1		GPD2
	IL1RAP		GPER1
	IL1RL1		GPI
	IL1RN		GPIHBP1
	IL22		GPKOW
	IL23R		GPLD1
	IL26		GPR119
	IL27		GPR132
	IL2RA		GPR137C
	IL2RB		GPR35
	IL32		GPR55
	IL33		GPR6
	IL37		GPR84
	IL4I1		GPS2
	IL6		GPX1
	IL6R		GPX3

	IL6ST		GPX4
	IL9		GRAP2
	ILF3		GREM1
	ILK		GRIN1
	ILKAP		GRIN2A
	IMMT		GRIN2B
	INPP5D		GRIN2C
	INPP5K		GRIN2D
	INS		GRIN3A
	INSIG2		GRIN3B
	INSRR		GRK2
	IRAK1		GRK5
	IRAK3		GRM3
	IRAK4		GRN
	IRF3		GSC
	IRF5		GSK3A
	IRF6		GSK3B
	IRF8		GSR
	IRF9		GSS
	IRS1		GSTA4
	IRX1		GSTM1
	ISL1		GSTO1
	ITCH		GTPBP1
	ITGA2		GUCY1A1
	ITGA2B		GUK1
	ITGA5		GUSB
	ITGA6		GYPA
	ITGB1		GYPC
	ITGB1BP2		GYPE
	ITGB2		GZMB
	ITGB3		H6PD
	ITIH4		HABP2
	ITLN1		HACD4
	ITPRID2		HAL
	IVD		HAMP
	IVL		HAS2
	IVNS1ABP		HAS3
	JAG1		HAVCR1
	JAK2		HBEGF
	JCAD		HCAR2
	JDP2		HCRT
	JMJD1C		HDAC1
	JPH2		HDAC2
	JUN		HDAC3
	JUP		HDAC4
	KAT8		HDAC5
	KBTBD7		HDAC6

	KCNE1		HDAC9
	KCNE2		HDC
	KCNE4		HDLBP
	KCNH2		HECA
	KCNJ11		HFE
	KCNK10		HGF
	KCNK18		HGFAC
	KCNK2		HGS
	KCNK3		HHEX
	KCNK9		HHIP
	KCNMA1		HHIPL1
	KCNN4		HIF1A
	KCNQ1		HILPDA
	KCTD2		HIST1H2BJ
	KDM1A		HIST1H4L
	KDM5D		HIVEP2
	KDR		HLA-A
	KIAA1217		HLA-C
	KIF3A		HLA-DMB
	KIF6		HLA-DOA
	KIR3DL3		HLA-DQA2
	KITLG		HLA-DQB1
	KL		HLA-DRA
	KLF10		HLA-DRB1
	KLF2		HM13
	KLF4		HMBOX1
	KLK1		HMBS
	KLK3		HMG20A
	KLK6		HMGA1
	KLKB1		HMGA2
	KLRB1		HMGCR
	KLRC1		HMGXB3
	KLRD1		HMOX1
	KLRG1		HMOX2
	KLRK1		HNF1A
	KMT2A		HNF1B
	KNG1		HNF4A
	KRT1		HNRNPD
	KRT10		HNRNPK
	KRT14		HOXA10
	KRT18		HOXA11
	KRT25		HOXA6
	KRT28		HOXA9
	KRT3		HOXC11
	KRT74		HOXC4
	KRT75		HOXC5
	KRT76		HOXD4

	KRT77		HPCAL1
	KRT85		HPD
	KRT86		HPGD
	KRTAP1-1		HPGDS
	LAG3		HPN
	LAIR1		HPR
	LAP3		HPS1
	LARP6		HPS3
	LAT		HPSE
	LAX1		HPX
	LBP		HRAS
	LCAT		HRH1
	LCK		HRH2
	LCN2		HS3ST1
	LCT		HSD11B1
	LDHA		HSD11B2
	LDHB		HSD17B6
	LEP		HSF1
	LEPR		HSH2D
	LGALS1		HSP90AB1
	LGALS2		HSP90B1
	LGALS3		HSPA12B
	LGALS3BP		HSPA1A
	LHFPL2		HSPA1B
	LIF		HSPA5
	LIMD1		HSPA8
	LIMS1		HSPB1
	LIMS2		HSPB3
	LIPA		HSPB8
	LIPC		HSPD1
	LIPE		HTR1B
	LIPG		HTR2A
	LMCD1		HTR4
	LMNA		HTR5A
	LOX		HTRA2
	LPA		HYLS1
	LPAL2		HYOU1
	LPAR3		IAPP
	LPL		IARS
	LPO		IBSP
	LPP		ICAM1
	LPXN		ICOS
	LRG1		ICOSLG
	LRP1		ID3
	LRP2		IDE
	LRP5		IDH2
	LRP6		IDO1

	LRP8		IDS
	LRRC3B		IFI16
	LRRFIP1		IFI30
	LRRK2		IFI44
	LTA		IFIH1
	LTA4H		IFNA2
	LTB		IFNAR1
	LTC4S		IFNB1
	LTF		IFNGR2
	LXN		IFT122
	LYST		IGF1
	LYVE1		IGF1R
	MAEA		IGF2
	MAF		IGF2BP2
	MAG		IGF2R
	MALT1		IGFBP1
	MAMLD1		IGFBP2
	MANF		IGFBP3
	MANSC1		IGFBP4
	MAP2K1		IGFBP5
	MAP2K3		IGFBP7
	MAP2K5		IGHD
	MAP2K6		IGLL1
	MAP3K3		IKBKB
	MAP3K5		IKBKE
	MAP4		IKBKG
	MAP4K5		IKZF2
	MAP6		IL10RB
	MAP9		IL11
	MAPK3		IL12A
	MAPK7		IL13
	MAPK8		IL15
	MAPKAPK2		IL15RA
	MAPKAPK3		IL16
	MAPKAPK5		IL17A
	MARCKS		IL17C
	MARS		IL17D
	MASP1		IL17F
	MASP2		IL17RA
	MAT2A		IL17RB
	MAT2B		IL18
	MATK		IL18BP
	MATN1		IL19
	MAZ		IL1A
	MB		IL1B
	MBL2		IL1F10
	MBNL1		IL1R1

	MBTPS1		IL1R2
	MC4R		IL1RL1
	MCAT		IL1RN
	MCF2L		IL21
	MCHR2		IL21R
	MCIDAS		IL22
	MCU		IL23A
	MDFIC		IL23R
	MDK		IL24
	MDM2		IL25
	MED23		IL26
	MEF2A		IL2RB
	MEFV		IL2RG
	MEN1		IL31RA
	MERTK		IL32
	MEX3B		IL33
	MFAP4		IL34
	MFF		IL37
	MFGE8		IL4R
	MGP		IL6
	MIA		IL6R
	MIA3		IL6ST
	MICA		IL7R
	MIF		IL9
	MLANA		ILK
	MLKL		ILKAP
	MLN		IMPA2
	MLXIPL		INF2
	MLYCD		INO80D
	MMD		INPP5K
	MME		INPPL1
	MMP1		INS
	MMP10		INSIG1
	MMP12		INSIG2
	MMP13		INTS6
	MMP19		INVS
	MMP2		IPO5
	MMP20		IQSEC1
	MMP28		IRAK1
	MMP3		IRAK3
	MMP7		IRAK4
	MMP8		IRF1
	MMP9		IRF2BP2
	MMRN1		IRF3
	MMUT		IRF5
	MORF4L1		IRF7
	MPG		IRF8

	MPI		IRGM
	MRAP		IRS1
	MRAS		IRS2
	MRC1		IRX4
	MRPS6		ISOC1
	MRRF		ITCH
	MRS2		ITGA2
	MRTFA		ITGA2B
	MSC		ITGA4
	MST1		ITGA5
	MSTN		ITGA6
	MTAP		ITGA8
	MT-CO1		ITGAD
	MT-CYB		ITGAL
	MTHFD1		ITGAM
	MTHFR		ITGAX
	MT-ND2		ITGB2
	MTNR1A		ITGB3
	MTOR		ITGB4
	MTR		ITGB5
	MT-RNR2		ITGB7
	MTRR		ITIH4
	MTSS1		ITLN1
	MTTP		ITPR2
	MTX1		IVD
	MUC1		JAG1
	MUC16		JAK1
	MUC5AC		JAK2
	MVD		JAK3
	MVP		JAM3
	MXD1		JAML
	MYBL2		JAZF1
	MYBPC3		JCAD
	MYBPHL		JMJD1C
	MYD88		JUN
	MYDGF		JUP
	MYEOV		KALRN
	MYH10		KAT8
	MYH7		KCNC4
	MYL2		KCNE2
	MYL3		KCNJ11
	MYL7		KCNJ3
	MYOCD		KCNMA1
	MYOG		KCNMB1
	MYOZ2		KCNN3
	MYRF		KCNN4
	NAAA		KCNQ1

	NAB1		KCTD15
	NAMPT		KCTD2
	NANOS1		KDM4A
	NANOS3		KDM5B
	NAP1L4		KDM5D
	NBEAL1		KDR
	NBN		KERA
	NCAM1		KIF20B
	NCF1		KIF6
	NCR3		KIRREL3
	NDNF		KISS1
	NDP		KISS1R
	NDRG1		KITLG
	NDUFS4		KL
	NEB		KLF10
	NECTIN3		KLF15
	NEDD4L		KLF2
	NEIL3		KLF4
	NELFCD		KLF5
	NES		KLHDC3
	NEU1		KLHDC8B
	NF1		KLK1
	NF2		KLK3
	NFATC3		KLKB1
	NFE2L2		KLRC2
	NFKB1		KLRD1
	NFKBIA		KLRG1
	NFKBIB		KLRK1
	NFKBIL1		KMT2E
	NGB		KRT14
	NGF		KRT18P32
	NIBAN2		KRT27
	NINL		KRT8
	NISCH		LACTB
	NKX2-5		LAG3
	NLRP2		LAIR1
	NLRP3		LBH
	NMI		LBP
	NMS		LCAT
	NNT		LCN2
	NOC2L		LCP1
	NOD1		LDB2
	NOD2		LDHA
	NOS1		LECT2
	NOS1AP		LEF1
	NOS2		LEP
	NOS3		LEPR

	NOTCH1		LGALS1
	NOTCH3		LGALS3
	NOX4		LGALS3BP
	NOX5		LGI1
	NPAT		LGMN
	NPC1L1		LIAS
	NPFF		LIF
	NPPA		LILRB1
	NPPB		LILRB4
	NPPC		LIN9
	NPR1		LINC00305
	NPR2		LINGO1
	NPR3		LIPA
	NPTX1		LIPC
	NPY		LIPE
	NPY2R		LIPG
	NPY4R		LIPI
	NPY6R		LMCD1
	NR0B1		LMNA
	NR1D2		LMNB1
	NR1H2		LMO4
	NR1H3		LMOD1
	NR1H4		LMX1B
	NR2E3		LOX
	NR3C1		LPA
	NR3C2		LPAR1
	NR4A1		LPAR3
	NR4A2		LPAR4
	NRDC		LPCAT3
	NRF1		LPIN1
	NRG1		LPIN2
	NRG3		LPIN3
	NRG4		LPL
	NRGN		LPO
	NRP1		LPP
	NTN1		LPXN
	NTNG2		LRBA
	NTRK1		LRG1
	NTRK2		LRIG1
	NTS		LRIT1
	NUCB2		LRP1
	OAZ1		LRP1B
	OBSCN		LRP2
	ODC1		LRP2BP
	OGA		LRP5
	OGG1		LRP6
	OGN		LRP8

	OGT		LRRC32
	OLR1		LRRTM4
	ONECUT2		LSAMP
	ONECUT3		LSM1
	OPA1		LSR
	OPLAH		LSS
	OPN1MW		LTA
	OPN1SW		LTA4H
	OPRL1		LTB
	OPRM1		LTB4R
	OR13G1		LTBR
	ORM2		LTF
	OSM		LUM
	OSTN		LUZP2
	OTOR		LXN
	OXCT1		LY75
	OXT		LYN
	OXTR		LYPLA1
	P2RX7		LYST
	P2RY1		LYVE1
	P2RY11		LYZ
	P2RY12		MACF1
	P2RY13		MACROD2
	P2RY2		MADCAM1
	P2RY4		MAEA
	P2RY6		MAF
	P3H3		MAFB
	P4HA2		MALT1
	P4HB		MAMLD1
	PAEP		MAOA
	PAF1		MAP2K1
	PAK1		MAP2K5
	PALLD		MAP3K5
	PAPOLG		MAP3K7
	PAPPA		MAP4K4
	PAPPA2		MAP4K5
	PARD6A		MAP6
	PARK7		MAP9
	PARP1		MAPK1
	PAX6		MAPK10
	PBRM1		MAPK11
	PBX2P1		MAPK14
	PCBD1		MAPK3
	PCIF1		MAPK6
	PCNA		MAPK7
	PCSK2		MAPK8
	PCSK5		MAPK9

	PCSK6		MAPKAPK2
	PCSK7		MAPKAPK5
	PCSK9		MAPT
	PCTP		MARS
	PCYT1A		MAS1
	PDC		MASP1
	PDCD1		MASP2
	PDCD4		MATN1
	PDE3A		MATN2
	PDE3B		MAZ
	PDE4D		MB
	PDE5A		MBD2
	PDGFC		MBL2
	PDGFD		MBTPS1
	PDGFRA		MBTPS2
	PDGFRB		MC1R
	PDIA6		MC4R
	PDLIM7		MCAT
	PDPK1		MCCC1
	PDX1		MCF2L
	PDXK		MCIDAS
	PDZD2		MCPH1
	PDZK1		MCTP1
	PEAR1		MDK
	PELI1		MDM2
	PENK		MECP2
	PEPD		MED17
	PER2		MED28
	PEX6		MEF2A
	PF4		MEF2C
	PFN1		MEFV
	PGAM5		MERTK
	PGD		METRN
	PGF		MFAP4
	PGLYRP1		MFGE8
	PGRMC1		MFN2
	PHACTR1		MGLL
	PHGDH		MGMT
	PHLDA1		MGP
	PHLPP2		MIA
	PI3		MIA3
	PIAS1		MICA
	PICALM		MICAL3
	PIGF		MICU1
	PIK3C2A		MICU3
	PIK3C2G		MIF
	PIK3CA		MIR137

	PIK3CD		MIR6792
	PIK3CG		MLANA
	PIM1		MLKL
	PINK1		MLN
	PIP		MLXIPL
	PIPOX		MLYCD
	PIR		MMD
	PITX1		MME
	PITX2		MMEL1
	PKM		MMP1
	PKN1		MMP10
	PLA2G1B		MMP11
	PLA2G4A		MMP12
	PLA2G7		MMP13
	PLA2R1		MMP16
	PLAUR		MMP17
	PLCL2		MMP19
	PLD1		MMP2
	PLD2		MMP3
	PLK2		MMP7
	PLK3		MMP8
	PLK4		MMP9
	PLN		MMRN1
	PLPP3		MMRN2
	PLTP		MMUT
	PMCH		MNDA
	PML		MORC3
	PNN		MPC2
	PNOC		MPI
	PNPLA3		MPL
	PODXL		MPP2
	POFUT1		MPPED2
	POMC		MPZL2
	PON1		MRAS
	POR		MRC1
	POSTN		MROH2A
	POU2F1		MRPS6
	POU2F3		MS4A1
	POU3F4		MSANTD3-TMEFF1
	PPARA		MSC
	PPARD		MSH2
	PPARG		MSH5
	PPARGC1A		MSN
	PPARGC1B		MSR1
	PPIA		MST1
	PPIF		MSTN

	PPIP5K1		MSX1
	PPL		MT1DP
	PPP1R10		MT1F
	PPRC1		MT2A
	PPY		MTAP
	PRDM2		MTCH2
	PRDX2		MT-CO1
	PRDX3		MT-CYB
	PRDX5		MTFMT
	PRH1		MTHFD1
	PRKAR2A		MTHFD1L
	PRKAR2B		MTHFD2
	PRKCA		MTHFR
	PRKCB		MTHFS
	PRKCD		MTMR3
	PRKD1		MT-ND1
	PRKG1		MT-ND2
	PRKN		MT-ND3
	PRMT3		MT-ND4
	PRMT5		MT-ND4L
	PRNP		MT-ND5
	PROCR		MT-ND6
	PROKR1		MTOR
	PRORP		MTR
	PRSS27		MT-RNR1
	PRTN3		MT-RNR2
	PSD		MTRNR2L8
	PSD4		MTRR
	PSMA6		MTSS1
	PSMA7		MTTP
	PSMG1		MTX1
	PSRC1		MUC1
	PTCRA		MUC16
	PTEN		MUC5AC
	PTGDR		MUTYH
	PTGER1		MVD
	PTGER2		MVK
	PTGES		MVP
	PTGIR		MXD1
	PTGIS		MYC
	PTGR1		MYD88
	PTGS1		MYH11
	PTGS2		MYH15
	PTH		MYH7B
	PTH1R		MYH9
	PTHLH		MYLIP
	PTK2		MYLK

	PTK2B		MYOC
	PTN		MYOCD
	PTPN1		MYOM2
	PTPN11		MYRF
	PTPN22		NAAA
	PTPRC		NAMPT
	PTPRD		NANOS1
	PTPRU		NANOS2
	PTS		NANOS3
	PXDN		NAP1L4
	PYCARD		NAPEPLD
	PZP		NAT2
	QDPR		NAT8
	QKI		NAV2
	QPCT		NAXE
	RACK1		NBEA
	RAD50		NBL1
	RAD51D		NBN
	RAF1		NBPF3
	RAG2		NCAM1
	RAMP1		NCAM2
	RAN		NCEH1
	RAPGEF1		NCF1
	RAPGEF3		NCOA6
	RAPGEF5		NCOR1
	RARRES2		NDC80
	RASA1		NDRG1
	RASA4		NDRG2
	RASGRF2		NDUFA1
	RASL12		NDUFA10
	RBM10		NDUFA11
	RBP1		NDUFA12
	RBP4		NDUFA13
	RBPJ		NDUFA2
	RCAN1		NDUFA3
	RCN1		NDUFA4
	RECQL5		NDUFA4L2
	REG1A		NDUFA5
	REM1		NDUFA6
	REN		NDUFA7
	RET		NDUFA8
	RETN		NDUFA9
	RFLNA		NDUFAB1
	RGN		NDUFAF1
	RGS1		NDUFAF2
	RGS5		NDUFAF3
	RGS7		NDUFAF4

	RHBDF2		NDUFB1
	RHCE		NDUFB10
	RHOJ		NDUFB11
	RIPK1		NDUFB2
	RIPK2		NDUFB3
	RIPK3		NDUFB4
	RIPOR2		NDUFB5
	RNASE1		NDUFB6
	RNASE2		NDUFB7
	RNASE3		NDUFB8
	RNF13		NDUFB9
	RNF182		NDUFC1
	RNH1		NDUFC2
	RNLS		NDUFS1
	ROCK1		NDUFS2
	ROCK2		NDUFS3
	ROS1		NDUFS4
	RPIA		NDUFS5
	RPL17		NDUFS6
	RRM1		NDUFS7
	RRS1		NDUFS8
	RSAD2		NDUFV1
	RUNX1		NDUFV2
	RXRA		NDUFV3
	RYR1		NECTIN1
	RYR2		NECTIN2
	RYR3		NEDD4
	S100A1		NEDD9
	S100A11		NEFL
	S100A12		NEGR1
	S100A4		NEIL3
	S100A6		NELFCD
	S100A8		NES
	S100A9		NEU1
	S100B		NEUROD4
	S100P		NEXN
	S1PR1		NEXN-AS1
	S1PR2		NF1
	S1PR3		NF2
	SARS		NFAT5
	SAT1		NFATC1
	SAYSD1		NFE2L2
	SCAF1		NFIA
	SCAI		NFKB1
	SCARB1		NFKB2
	SCD		NFKBIA
	SCG2		NGB

	SCN4A		NGF
	SCT		NGFR
	SCUBE1		NHEJ1
	SDC1		NID1
	SDC2		NIFK
	SDC4		NINJ2
	SDK1		NISCH
	SEC14L2		NKAPL
	SEC24D		NLRC4
	SELE		NLRP1
	SELENOP		NLRP11
	SELL		NLRP3
	SELP		NME1
	SELPLG		NNMT
	SEMA3A		NNT
	SEMA3F		NOC2L
	SENP1		NOD1
	SERHL		NOD2
	SERPINA1		NOP56
	SERPINA12		NOS1
	SERPINA3		NOS1AP
	SERPINA4		NOS2
	SERPINA5		NOS3
	SERPINA6		NOTCH1
	SERPINA7		NOTCH3
	SERPINB2		NOX1
	SERPINB6		NOX4
	SERPINC1		NOX5
	SERPIND1		NOXA1
	SERPINE1		NPC1
	SERPINF1		NPC1L1
	SERPINF2		NPHS1
	SERPING1		NPHS2
	SERPINH1		NPPA
	SESN2		NPPB
	SETD2		NPPC
	SF3A2		NPR1
	SFRP1		NPR2
	SFRP2		NPR3
	SFRP4		NPS
	SFRP5		NPTX1
	SFTPA1		NPVF
	SFTPB		NPY
	SFTPD		NQO1
	SGCA		NR0B1
	SGCD		NR0B2
	SGK1		NR1D2

	SGSM3		NR1H2
	SH2B1		NR1H3
	SH2B3		NR1H4
	SH3BP4		NR1I2
	SH3BP5		NR1I3
	SHBG		NR2C2
	SHC2		NR2F2
	SHD		NR3C1
	SHF		NR3C2
	SIK3		NR4A1
	SIRT1		NR4A2
	SIRT2		NR4A3
	SIRT3		NR5A2
	SIRT5		NR6A1
	SIRT6		NRDE2
	SKAP1		NRF1
	SKAP2		NRG1
	SKIL		NSF
	SKP1		NSMCE1
	SLAMF6		NT5C1A
	SLAMF7		NT5C2
	SLC11A1		NTF3
	SLC12A1		NTM
	SLC12A3		NTN1
	SLC12A8		NTRK1
	SLC17A5		NTRK2
	SLC19A1		NUBPL
	SLC1A7		NUCB2
	SLC22A3		NUP205
	SLC22A4		NUP43
	SLC22A5		NUS1
	SLC25A1		NUTM2F
	SLC25A10		NXPH4
	SLC25A16		OCM2
	SLC25A3		ODC1
	SLC25A4		OGA
	SLC28A1		OGG1
	SLC29A1		OGN
	SLC2A1		OGT
	SLC2A4		OIP5
	SLC2A4RG		OLR1
	SLC33A1		OMD
	SLC4A4		OMP
	SLC5A1		OPN1LW
	SLC5A2		OPN1SW
	SLC5A3		OPN4
	SLC5A5		OPRL1

	SLC5A6		OPTN
	SLC6A18		OR2D2
	SLC6A3		OR2M7
	SLC6A4		OR5H2
	SLC8A1		ORMDL3
	SLC9A1		OSBP
	SLCO1B1		OSBP2
	SLCO1C1		OSBPL10
	SLCO3A1		OSBPL1A
	SLCO6A1		OSBPL8
	SLN		OSCAR
	SLPI		OSGIN1
	SLURP1		OSM
	SMAD1		OSMR
	SMAD2		OVOL2
	SMAD3		OXR1
	SMAD4		P2RX1
	SMAD7		P2RX7
	SMAD9		P2RY1
	SMARCA4		P2RY12
	SMC2		P2RY13
	SMC3		P2RY2
	SMG6		P2RY4
	SMPD1		P2RY6
	SMPD2		P3H3
	SMPD4		P4HB
	SMS		PACS2
	SMTN		PADI4
	SNX17		PAEP
	SOAT1		PAGR1
	SOCS3		PAH
	SOD2		PAK1
	SORBS2		PALLD
	SORBS3		PAM
	SORD		PANX1
	SOX7		PAPPA
	SP140		PAQR8
	SP7		PARG
	SPAG8		PARK7
	SPARC		PARP1
	SPATA7		PARP14
	SPECC1L		PATE1
	SPEF2		PATZ1
	SPESP1		PAX2
	SPHK1		PBRM1
	SPINT2		PBX2P1
	SPN		PCBD1

	SPON1		PCDH8
	SPP1		PCDH9
	SPRR3		PCNA
	SQSTM1		PCOLCE2
	SRA1		PCSK1
	SRC		PCSK5
	SREBF1		PCSK6
	SREBF2		PCSK7
	SRF		PCSK9
	SRGN		PCTP
	SRI		PCYT1A
	SRY		PCYT1B
	SS18L1		PDC
	SSB		PDCD4
	SST		PDCD5
	SSTR2		PDE1A
	ST13		PDE1C
	ST3GAL4		PDE2A
	ST6GALNAC1		PDE3A
	ST8SIA4		PDE3B
	STAB1		PDE4D
	STAG2		PDE5A
	STAT1		PDE9A
	STAT3		PDGFA
	STAT4		PDGFB
	STC1		PDGFC
	STC2		PDGFD
	STH		PDGFRA
	STIM1		PDGFRB
	STK11		PDIA2
	STS		PDK4
	STXBP2		PDLIM1
	SULT1E1		PDLIM7
	SULT2A1		PDPN
	SULT4A1		PDX1
	SUMO1		PDYN
	SUV39H1		PDZD8
	SYNM		PDZK1
	SYT1		PEBP1
	TAB1		PEG10
	TACR1		PELI2
	TACR3		PELI3
	TALDO1		PEMT
	TAOK2		PES1
	TAPBP		PF4
	TAS2R50		PF4V1
	TBC1D1		PFN1

	TBC1D10C		PGF
	TBC1D2		PGLYRP1
	TBC1D4		PGP
	TBCA		PGR
	TBCD		PGRMC1
	TBP		PHACTR1
	TBPL1		PHEX
	TBX1		PHF14
	TBX21		PHF21A
	TBXA2R		PHGDH
	TBXAS1		PHLDA1
	TBXT		PHPT1
	TCF21		PI3
	TCF7L2		PIAS1
	TCFL5		PIAS3
	TCHP		PIAS4
	TCN1		PIK3CA
	TCP1		PIK3CD
	TEF		PIK3CG
	TENM1		PIK3R1
	TENM3		PIK3R3
	TERT		PINX1
	TES		PINX1
	TET1		PITRM1
	TF		PITX2
	TFAM		PKM
	TFAP2A		PKN2
	TFEB		PKNOX2
	TFF3		PLA1A
	TFG		PLA2G10
	TFPI		PLA2G15
	TGFB1		PLA2G1B
	TGFB3		PLA2G2A
	TGFBI		PLA2G2D
	TGFBR1		PLA2G2E
	THAP5		PLA2G2F
	THBD		PLA2G3
	THBS1		PLA2G4A
	THBS2		PLA2G7
	THBS4		PLAC1
	THPO		PLAUR
	THRA		PLCB2
	THRB		PLCB3
	TIGAR		PLCG2
	TIMM8A		PLD2
	TIMP1		PLEKHO1
	TIMP2		PLIN1

	TIMP4		PLIN2
	TKT		PLIN3
	TKTL1		PLK4
	TLE5		PLN
	TLN1		PLPP2
	TLR1		PLPP3
	TLR2		PLTP
	TLR3		PLXNB1
	TLR4		PLXNB2
	TLR7		PLXND1
	TLR9		PML
	TLX1NB		PMPCA
	TLX2		PNKD
	TM6SF2		PNLIP
	TMEFF1		PNN
	TMEFF2		PNOC
	TMEM123		PNPLA2
	TMEM38B		PNPLA3
	TMSB4X		PODN
	TNC		PODXL
	TNF		POLG
	TNFAIP6		POLK
	TNFRSF10B		POLR1D
	TNFRSF10C		POLR2A
	TNFRSF11B		POLR2I
	TNFRSF13C		POMC
	TNFRSF1A		PON1
	TNFRSF1B		POP1
	TNFRSF4		POR
	TNFRSF9		POSTN
	TNFSF10		POU2F1
	TNFSF11		POU2F3
	TNFSF13B		PPARA
	TNFSF18		PPARD
	TNFSF4		PPARG
	TNNC1		PPARGC1A
	TNNI3		PPARGC1B
	TNNI3K		PPBP
	TNNT1		PPCDC
	TNNT2		PPIA
	TNRC6B		PPL
	TNS1		PPM1A
	TNS3		PPM1B
	TNXB		PPM1D
	TOLLIP		PPP1R12A
	TOPBP1		PPP1R3B
	TP53		PPP2R2A

	TP53INP1		PPP2R2C
	TP53INP2		PPP4R2
	TPH1		PQBP1
	TPI1		PRB2
	TPM1		PRC1
	TPO		PRCP
	TPPP		PRDX1
	TPSG1		PRDX2
	TPT1		PRDX5
	TRAF3IP2		PRDX6
	TRAF6		PRELP
	TRAP1		PREX1
	TREM1		PRG4
	TRH		PRH1
	TRIB1		PRIM1
	TRIB3		PRKAA1
	TRIM39		PRKCA
	TRIM54		PRKCB
	TRIM63		PRKCH
	TRIM72		PRKD1
	TRIT1		PRKG1
	TRPA1		PRKG2
	TRPC3		PRLR
	TRPC4		PRMT1
	TRPC6		PRMT2
	TRPM2		PRMT3
	TRPM4		PRNP
	TRPV1		PROCR
	TRPV2		PROS1
	TSC22D3		PROX1
	TSN		PRRC2A
	TSPAN10		PRRX1
	TST		PRSS27
	TTC32		PRTN3
	TTL		PSD
	TTN		PSEN1
	TTPA		PSMA6
	TTR		PSMA7
	TUBB		PSMB8
	TUBB1		PSME3
	TUBB2A		PSMG1
	TUBB2B		PSRC1
	TUBB3		PSTPIP2
	TUBB4A		PTAFR
	TUBB4B		PTEN
	TUBB6		PTGDS
	TUBB8		PTGES

	TUSC1		PTGIR
	TXN		PTGIS
	TXNDC9		PTGS1
	TXNIP		PTGS2
	TXNL1		PTH
	TXNL4B		PTHLH
	TXNRD2		PTK2
	TYMP		PTK2B
	TYR		PTMA
	TYRO3		PTN
	UBD		PTOV1
	UBE2K		PTPN1
	UBE2Z		PTPN11
	UCHL1		PTPN2
	UCN		PTPN22
	UCN2		PTPN6
	UCN3		PTPRC
	UCP2		PTPRG
	UCP3		PTPRN2
	UGCG		PTPRU
	UGT1A1		PTS
	UMOD		PVR
	UNC5B		PXDN
	URI1		PYCARD
	UTP25		PYGB
	UTS2		PYY
	VAMP8		PZP
	VASP		QDPR
	VCAM1		QKI
	VCAN		QPCT
	VCL		QPCTL
	VDR		QSOX1
	VEGFA		RAB37
	VEGFB		RAC2
	VEGFC		RAD21
	VEGFD		RAF1
	VHL		RAG1
	VIM		RAG2
	VIP		RALBP1
	VKORC1		RAPGEF3
	VLDLR		RAPGEF5
	VNN3		RARA
	VPS51		RARB
	VWF		RARRES2
	WDR12		RASD1
	WDR64		RASEF
	WFDC1		RASGRP2

	WIF1		RB1
	WLS		RBFOX1
	WNT4		RBM10
	WT1		RBM38
	WTAP		RBM45
	XBP1		RBP4
	XCL1		RBPJ
	XIAP		RBPMS
	XPR1		RCAN1
	XRCC1		RCN2
	XRCC3		RDX
	YBX1		REEP5
	YTHDF3		REG1A
	ZBTB12		RELA
	ZC3HC1		RELB
	ZDHHC2		RELN
	ZEB2		REN
	ZFHX3		REST
	ZFP90		RETN
	ZGLP1		RETNLB
	ZGPAT		RFC3
	ZMYM2		RFTN1
	ZNF185		RFX5
	ZNF202		RGCC
	ZNF433		RGL1
	ZNF536		RGN
	ZNF627		RGS1
			RGS2
			RGS3
			RGS4
			RGS5
			RGS6
			RGS7
			RHBDF2
			RHD
			RHOA
			RHOB
			RHOF
			RILPL2
			RIMS1
			RIPK2
			RIPK3
			RNASE1
			RNASE2
			RNASE3
			RNASE6
			RNF112

			RNF2
			RNF213
			RNF5
			RNLS
			RNU7-62P
			ROCK1
			ROCK2
			ROR2
			RORA
			RORC
			RP9
			RPA2
			RPE
			RPGRIP1L
			RPIA
			RPL17
			RPL27A
			RPS3A
			RPS6KB1
			RPTOR
			RRS1
			RSAD2
			RSU1
			RTCA
			RTEL1
			RTL1
			RTN3
			RTN4
			RTRAF
			RUNX2
			RUNX3
			RXRA
			RXRG
			RYR2
			RYR3
			S100A1
			S100A10
			S100A11
			S100A12
			S100A4
			S100A6
			S100A7
			S100A8
			S100A9
			S100B
			S1PR1
			S1PR2

			S1PR3
			SAA4
			SAG
			SALL1
			SAMD1
			SAMD9
			SAMHD1
			SAMSN1
			SARS
			SASH1
			SAT1
			SCAF1
			SCAI
			SCARA5
			SCARB1
			SCARB2
			SCARF1
			SCD
			SCG3
			SCML4
			SCN5A
			SCP2
			SCT
			SCUBE1
			SCUBE2
			SDC1
			SDK2
			SEC14L2
			SECISBP2
			SELE
			SELENOK
			SELENOP
			SELENOS
			SELL
			SELP
			SELPLG
			SEMA3C
			SEMA3E
			SEMA4D
			SEMA7A
			SENP2
			SEPHS1
			SERGEF
			SERHL
			SERP2
			SERPINA1
			SERPINA10

			SERPINA12
			SERPINA3
			SERPINA4
			SERPINA5
			SERPINA6
			SERPINA7
			SERPINA9
			SERPINB2
			SERPINB9
			SERPINC1
			SERPIND1
			SERPINE1
			SERPINE2
			SERPINF1
			SERPINF2
			SERPING1
			SESN2
			SETBP1
			SETD2
			SETD7
			SF3B4
			SFRP4
			SFRP5
			SFTA3
			SFTPB
			SFTPD
			SGCA
			SGK1
			SGMS1
			SGMS2
			SGPL1
			SH2B3
			SH2D3C
			SH3BP4
			SHBG
			SHC1
			SHISA9
			SIGLEC1
			SIGLEC5
			SIGLEC9
			SIRPA
			SIRT1
			SIRT3
			SIRT5
			SIRT6
			SIRT7
			SKAP2

			SKIL
			SLAMF7
			SLC10A2
			SLC11A2
			SLC12A3
			SLC14A1
			SLC14A2
			SLC16A3
			SLC16A8
			SLC17A2
			SLC17A3
			SLC17A4
			SLC17A5
			SLC18A3
			SLC22A1
			SLC22A11
			SLC22A12
			SLC22A4
			SLC25A1
			SLC25A10
			SLC25A14
			SLC25A19
			SLC25A22
			SLC25A25
			SLC25A3
			SLC25A32
			SLC25A43
			SLC27A2
			SLC28A1
			SLC2A1
			SLC2A10
			SLC2A2
			SLC2A4
			SLC2A4RG
			SLC2A9
			SLC30A8
			SLC33A1
			SLC35A2
			SLC39A12
			SLC39A8
			SLC45A2
			SLC46A3
			SLC5A1
			SLC5A10
			SLC5A7
			SLC6A1
			SLC9A1

			SLCO2B1
			SLCO6A1
			SLPI
			SLTM
			SMAD1
			SMAD2
			SMAD3
			SMAD7
			SMC2
			SMG6
			SMOC2
			SMPD1
			SMPD2
			SMPD3
			SMPDL3A
			SMS
			SMTN
			SNCA
			SNF8
			SNRPG
			SNTB2
			SNX19
			SOAT1
			SOAT2
			SOCS1
			SOCS3
			SOCS6
			SOD1
			SOD2
			SORBS3
			SORCS1
			SORD
			SORL1
			SOS1
			SOST
			SOX18
			SOX6
			SP1
			SP2
			SPARC
			SPARCL1
			SPATA13
			SPDEF
			SPESP1
			SPHK1
			SPI1
			SPIN1

			SPN
			SPON1
			SPON2
			SPOPL
			SPP1
			SPRR3
			SPRTN
			SPRY1
			SPTLC1
			SPTLC3
			SPX
			SQLE
			SQSTM1
			SRD5A1
			SREBF1
			SREBF2
			SRF
			SRGN
			SRI
			SRM
			SRR
			SRSF1
			SRSF10
			SRSF2
			SRY
			SSB
			SSBP1
			SST
			SSTR2
			SSTR4
			SSTR5
			ST13
			ST14
			ST3GAL4
			ST6GAL1
			STAB1
			STAB2
			STAP2
			STARD3NL
			STAT1
			STAT2
			STAT3
			STAT4
			STAT6
			STC1
			STC2
			STEAP4

			STH
			STIM1
			STIM2
			STK11
			STK25
			STRIP2
			STS
			STXBP5L
			SUCNR1
			SULF2
			SULT1E1
			SUMO1
			SUMO4
			SYK
			SYNM
			SYNPO2
			SYT17
			TAB1
			TAB2
			TAC1
			TACR3
			TAF3
			TAGAP
			TAGLN
			TANC2
			TANK
			TAP1
			TARDBP
			TAS1R3
			TBC1D30
			TBCA
			TBP
			TBX18
			TBX20
			TBXA2R
			TBXAS1
			TCF21
			TCF3
			TCF7L2
			TCFL5
			TCHHL1
			TCHP
			TCIRG1
			TCP1
			TCTN1
			TEK
			TERF1

			TERT
			TES
			TET1
			TET2
			TF
			TFAM
			TFAP2A
			TFEB
			TFF1
			TFF3
			TFG
			TFPI
			TFPI2
			TGFB1
			TGFB1I1
			TGFB3
			TGFBI
			TGFBR1
			TGFBR2
			TGM5
			TGM7
			TH
			THADA
			THBD
			THBS1
			THBS2
			THBS4
			THEMIS
			THRA
			THSD7A
			TICAM1
			TIGIT
			TIMD4
			TIMM23
			TIMP1
			TIMP2
			TIMP4
			TJP1
			TLR1
			TLR10
			TLR2
			TLR3
			TLR4
			TLR5
			TLR6
			TLR7
			TLR8

			TLR9
			TLX1NB
			TLX2
			TMEFF1
			TMEFF2
			TMEM11
			TMEM134
			TMEM18
			TMEM50B
			TMEM98
			TMF1
			TMPRSS6
			TMSB10
			TMSB4X
			TMX1
			TNC
			TNF
			TNFAIP3
			TNFAIP6
			TNFAIP8L2
			TNFRSF10A
			TNFRSF10B
			TNFRSF11A
			TNFRSF11B
			TNFRSF12A
			TNFRSF13B
			TNFRSF13C
			TNFRSF14
			TNFRSF18
			TNFRSF1A
			TNFRSF1B
			TNFRSF25
			TNFRSF4
			TNFRSF6B
			TNFRSF8
			TNFRSF9
			TNFSF10
			TNFSF11
			TNFSF12
			TNFSF13
			TNFSF13B
			TNFSF14
			TNFSF15
			TNFSF18
			TNFSF4
			TNFSF8
			TNFSF9

			TNIK
			TNNI3
			TNNT1
			TNNT2
			TNNT3
			TNPO3
			TNRC6B
			TOLLIP
			TOR1A
			TP53
			TP53INP1
			TP53INP2
			TPM1
			TPM4
			TPO
			TPP2
			TPT1
			TRAF1
			TRAF5
			TRAF6
			TRAM2
			TREM1
			TRERF1
			TRIB3
			TRIM14
			TRIM21
			TRIM31
			TRIM38
			TRIM39
			TRIM5
			TRIM63
			TRIP12
			TRO
			TRPA1
			TRPC1
			TRPC3
			TRPC5
			TRPC6
			TRPM6
			TRPM7
			TRPM8
			TRPS1
			TRPV1
			TRPV2
			TRPV4
			TSBP1
			TSEN15

			TSHZ3
			TSLP
			TSPAN2
			TSPO
			TSPYL2
			TTC3
			TTC39B
			TTLL5
			TTLL7
			TTN
			TTPA
			TTR
			TUBA4A
			TUBA4B
			TUBB
			TUBB1
			TUBB2A
			TUBB2B
			TUBB3
			TUBB4A
			TUBB4B
			TUBB6
			TUBB8
			TWIST1
			TWSG1
			TXN
			TXNDC16
			TXNIP
			TXNL1
			TXNL4B
			TXNRD2
			TYMP
			TYR
			TYRO3
			TYROBP
			UBAC1
			UBAC2
			UBC
			UBE2D1
			UBE2K
			UBE2Z
			UBR5
			UCN
			UCN2
			UCN3
			UCP1
			UCP2

			UCP3
			UFM1
			UGCG
			UGT1A1
			UMOD
			UNC13C
			UNC5B
			UNK
			USF1
			USF2
			USP20
			USP48
			UTRN
			UTS2
			UTS2R
			VAMP3
			VAMP8
			VASP
			VAV1
			VCAM1
			VCL
			VCP
			VDAC1
			VDR
			VEGFA
			VEGFB
			VEGFC
			VEGFD
			VHL
			VIM
			VIP
			VIPR1
			VIT
			VKORC1
			VLDLR
			VNN1
			VPS51
			VSTM2B
			VWA8
			VWF
			WDR12
			WIPF1
			WNT5A
			WNT8B
			WRN
			WWOX
			XAB2

			XBP1
			XCL1
			XDH
			XPO7
			XPR1
			XRCC1
			XRCC4
			XRN2
			YAP1
			YBX1
			YBX3
			YME1L1
			YWHAQ
			YY1
			ZAP70
			ZBTB7A
			ZC3H12A
			ZC3HC1
			ZDHHC2
			ZEB1
			ZFHX3
			ZFHX4
			ZFP36
			ZFP64
			ZFYVE9
			ZG16B
			ZGLP1
			ZGPAT
			ZHX2
			ZMPSTE24
			ZMYM2
			ZNF202
			ZNF263
			ZNF330
			ZNF385D
			ZNF433
			ZNF462
			ZNF536
			ZNF609
			ZYG11B

Supplementary Table 4:

Lists of clustering coefficients of Ec_hepint, Ec_not hepint and Ec_hepint_random

	**Atherosclerosis**	**Myocarditis**	**Myocardial infarction**	**Myocardial Ischemia**
Ec_hepint	0.496	0.631	0.532	0.551
Ec_not hepint	0.330	0.524	0.349	0.431
Ec_hepint_random	0.348	0.662	0.310	0.514
